# Genomics of Heat Tolerance in Reproductive Performance Investigated in Four Independent Maternal Lines of Pigs

**DOI:** 10.3389/fgene.2020.00629

**Published:** 2020-06-30

**Authors:** Francesco Tiezzi, Luiz F. Brito, Jeremy Howard, Yi Jian Huang, Kent Gray, Clint Schwab, Justin Fix, Christian Maltecca

**Affiliations:** ^1^Department of Animal Science, North Carolina State University, Raleigh, NC, United States; ^2^Department of Animal Sciences, Purdue University, West Lafayette, IN, United States; ^3^Smithfield Premium Genetics, Rose Hill, NC, United States; ^4^The Maschhoffs LLC, Carlyle, IL, United States

**Keywords:** animal resilience, fertility, GxE, heat stress, heat sensitivity, relative humidity, reaction norm

## Abstract

Improving swine climatic resilience through genomic selection has the potential to minimize welfare issues and increase the industry profitability. The main objective of this study was to investigate the genetic and genomic determinism of tolerance to heat stress in four independent purebred populations of swine. Three female reproductive traits were investigated: total number of piglets born (TNB), number of piglets born alive (NBA) and average birth weight (ABW). More than 80,000 phenotypic and 12,000 genotyped individuals were included in this study. Genomic random-regression models were fitted regressing the phenotypes of interest on a set of 95 environmental covariates extracted from public weather station records. The models yielded estimates of (genomic) reactions norms for individual pigs, as indicator of heat tolerance. Heat tolerance is a heritable trait, although the heritabilities are larger under comfortable than heat-stress conditions (larger than 0.05 vs. 0.02 for TNB; 0.10 vs. 0.05 for NBA; larger than 0.20 vs. 0.10 for ABW). TNB showed the lowest genetic correlation (-38%) between divergent climatic conditions, being the trait with the strongest impact of genotype by environment interaction, while NBA and ABW showed values slightly negative or equal to zero reporting a milder impact of the genotype by environment interaction. After estimating genetic parameters, a genome-wide association study was performed based on the single-step GBLUP method. Heat tolerance was observed to be a highly polygenic trait. Multiple and non-overlapping genomic regions were identified for each trait based on the genomic breeding values for reproductive performance under comfortable or heat stress conditions. Relevant regions were found on chromosomes (SSC) 1, 3, 5, 6, 9, 11, and 12, although there were important regions across all autosomal chromosomes. The genomic region located on SSC9 appears to be of particular interest since it was identified for two traits (TNB and NBA) and in two independent populations. Heat tolerance based on reproductive performance indicators is a heritable trait and genetic progress for heat tolerance can be achieved through genetic or genomic selection. Various genomic regions and candidate genes with important biological functions were identified, which will be of great value for future functional genomic studies.

## Introduction

Heat stress (HS) is a major welfare issue in the swine industry, especially as global temperatures trend upwards. Over the past decades, genetic selection has played a major role in improving productive and reproductive performance in pigs (Merks, 2000; Hill, 2016; Zak et al., 2017). However, the enormous genetic progress achieved has been accompanied by a large increase in total metabolic heat production (Cabezón et al., 2016; Johnson, 2018; Johnson et al., 2019). Consequently, the animal's ability to cope with high ambient temperatures has been significantly reduced (Brown-Brandl et al., 2001; Renaudeau et al., 2011). In addition to creating welfare concerns, HS is responsible for significant economic losses to the swine industry (St-Pierre et al., 2003; Johnson and Baumgard, 2019).

Excessive heat and humidity are the main factor contributing to seasonal infertility, which has important economic implications as producers cannot maintain a constant flow of individuals throughout the farm during the whole year. Pigs have low capacity of dissipating body temperature under high ambient temperatures (Einarsson et al., 2008). HS leads to a reduction in the number of piglets born alive and weaned as well as litters produced per sow per year (Bertoldo et al., 2012). Furthermore, HS negatively impacts different stages of the sow reproductive life. For instance, Paterson et al. (1992) and Love et al. (1993) have shown how the attainment of puberty in gilts as well as early retained pregnancy losses could increase during the summer-autumn seasons. In addition, HS has been linked to reduced embryo establishment, resulting in a smaller number of piglets born per litter, particularly in gilts (Tummaruk et al., 2010).

There is evidence of genetic variability for heat tolerance (HT) in livestock species, including pigs (Carabaño et al., 2017; Misztal, 2017; Ansari-Mahyari et al., 2019; Zhang et al., 2019), particularly in the growing and finishing stages. In general, genetic analysis of HT in growing/finishing pigs are based on a heat-load function for live or carcass weight (Zumbach et al., 2008a,b; Fragomeni et al., 2016a). HT based on growth traits is moderately heritable (Fragomeni et al., 2016a,b), but there is a gap in knowledge on the genetic mechanisms of HS response in sows since only differences between maternal genetic lines have been reported (Peltoniemi et al., 1999; Bloemhof et al., 2008). Furthermore, the genetic correlations among reproductive traits expressed in different seasons of the year are lower than unity (Lewis and Bunter, 2011). This indicates that some genotypes that perform best in comfortable conditions might not be the top performer individuals under HS. The presence of genetic variability for HT enables selective breeding, which is a cost-effective approach for mitigating climatic HS in livestock (Renaudeau et al., 2012). For instance, Australian dairy cattle breeding programs are already reporting genomic breeding values for HT (Nguyen et al., 2016, 2017). Reaction-norm random-regression is an approach commonly used to study genotype by environment interactions (GxE, Rauw and Gomez-Raya, 2015) based on routinely measured traits such as reproductive records (Knap and Su, 2008; Silva et al., 2014). These models provide solutions for the across-environment genetic merit of individuals as well as their responsiveness to the environmental changes (i.e., the norm of their reaction; Rauw and Gomez-Raya, 2015).

Genotype by environment interactions have been largely neglected in the investigation of sow reproductive performance (Su et al., 2007; Putz et al., 2015) although genetic evaluations are routinely reported for several reproductive traits (Samorè and Fontanesi, 2016). Neglecting GxE could result in a deterioration of reproductive performance in pigs, especially in maternal lines. Several genomic regions have been identified to be associated with sows' reproductive performance in various breeds (Onteru et al., 2011; Schneider et al., 2012; Wang et al., 2018). Selective breeding based on genomic information is a promising alternative to reduce seasonality in swine reproductive performance as well as to improve HT. Therefore, the main objectives of this study were: (1) to investigate the impact of multiple environmental covariates on sow reproductive traits, (2) to estimate genetic variance components for HT, and, (3) to identify candidate genes and metabolic pathways that are associated with HS response in four independent swine populations.

## Materials and Methods

Animal welfare and ethics committee approval was not needed for this study as all the datasets used were provided by commercial breeding operations.

### Phenotypic Records

Datasets from four maternal-line pig populations were used for the current study. Nucleus-herd farrowing records were obtained from January 2008 to June 2016 for 11,163 Landrace (SPG_LR; 21,276 litters) and 12,184 Large White (SPG_LW; 27,794 litters) sows from the Smithfield Premium Genetics company (SPG) and from August 2011 to June 2016 for 11,537 Landrace (TML_LR; 24,934 litters) and 5,318 Yorkshire (TML_YS) sows (11,625 litters) from The Maschhoffs company (TML). Farms were located in North Carolina (*n* = 2), Texas (*n* = 1), Illinois (*n* = 6), Alabama (*n* = 1), Nebraska (*n* = 1) and Indiana (*n* = 1). The traits analyzed were: total number of piglets born (TNB), number of piglets born alive (NBA), and the average birth weight of the piglets (ABW, in kg). The phenotypic datasets were edited independently for each population by removing records deviating 3.5 SD from the mean.

### Weather Records

Weather records were obtained from the National Climatic Data Center Quality Controlled Local Climatological Data database at the National Oceanic and Atmospheric Administration (www.ncdc.noaa.gov/cdo-web/datatools/lcd?prior\=\N). Zip codes were obtained for each nucleus farm, converted to geological coordinates and matched to the closest weather station using the packages “zipcode” (Breen, 2012) and “geosphere” (Hijmans, 2019), available in the R software (R Core Team, 2016). In the present study, we assumed that the impact of HS on reproductive performance could be pinpointed to a specific time range from ovulation to farrowing (Bloemhof et al., 2012). Environmental covariates (ENV) used were obtained from the raw weather records. Litter-specific values were calculated for each week of the sows' reproductive cycle from 3 weeks prior to breeding through farrowing (19 week-intervals). The variables defined were: average of mean daily temperature (Mean.T), average of daily relative humidity (RH), average of maximum temperature (Max.T), average of minimum temperature (Min.T), and average temperature-humidity index (THI), calculated as Bohmanova et al. (2008). This generated a total of 95 (19 intervals by 5 variables) different ENV.

### Genomic Datasets

A total of 12,200 animals (10,899 sows and 1,301 boars) across the four populations were genotyped with either the PorcineSNP60K Bead Chip (Illumina Inc., San Diego, CA, USA; 61,566 SNPs) or the GGP Porcine HD v1 80K (GeneSeek Inc., Neogen Co., Lincoln, NE, USA; 68,529 SNPs). The missing genotypes were imputed using the FImpute v2.2 software (Sargolzaei et al., 2014) to the 60K Bead chip. Quality control of genotype data consisted of removing SNPs with minor allele frequency lower than 0.05, SNP call rate below 0.90 and extreme deviation from Hardy-Weinberg equilibrium (with a *p*-value smaller than 10^−6^). The total number of individuals with genotypes involved in further analyses is reported in Table 1. SPG populations had the largest number of individuals genotyped, with a total of 3,790 for SPG_LR and 5,271 for SPG_LR. TML populations had a lower number of individuals genotyped, with 1,587 for TML_LR and 1,552 for TML_YS. The final number of SNPs that passed the quality control was 48,911, 49,183, 44,824, and 44,875, for SPG_LR, SPG_LW, TML_LR, and TML_YS, respectively.

**Table 1:** Number of genotyped animals (sows and their sires) across the four populations used in this study.

**Population**	**Sows**			**Sires**		
	**60 K**	**80 K**	**Total**	**60K**	**80K**	**Total**
SPG_LR	2,085	1,086	3,171	406	213	619
SPG_LW	3,658	1,153	4,811	333	138	460
TML_LR	1,474	0	1,474	113	0	113
TML_YS	1,443	0	1,443	109	0	109

*Population: indicates the breeding company and breed, in which SPG, Smithfield Premium Genetics; TML, The Maschhoffs; LR, Landrace; LW, Large White; YS, Yorkshire (also known as Large White in other breeding companies)*.

### Statistical Analyses

Random regression models (RRM; Jamrozik and Schaeffer, 1997; Oliveira et al., 2019) coupled with the Single-step GBLUP approach (Aguilar et al., 2010) were used to regress the phenotypes (TNB, NBA, ABW) on the ENV to obtain reaction norms for the animal additive genetic effect. Legendre orthogonal polynomials (order = 1) were used to model the trajectory of phenotypic traits across environmental conditions, by running a model for each potential ENV. The random regression model is used as a reaction norm model, where each individual will be assigned two solutions for the genetic effect: a solution for the intercept term, expressing the performance where the environmental covariate is set to 0; and a solution for the slope term, which eliciting the change in performance given a unit change in the environmental covariate. The latter component can be considered as an indicator of plasticity. The model fitted for all the traits can be described as:

$$\begin{array}{l} y_{ijklmn}=\alpha 1+\beta \varphi_{1k}+CG_{i}+Par_{j}+a_{0l}1+a_{1l}\varphi_{1k}{+p}_{m}+e_{ijklmn} \end{array}$$

Where *y*_*ijklmn*_ is the nth phenotypic record for TNB, NBA or ABW, α is the intercept, *CG*_*i*_ is the fixed effect of the contemporary group (sow birth year), *Par*_*j*_ is the fixed effect of the parity of the sow (1, 2, 3, 4, 5 and higher), β is the fixed regression coefficient on the ENV, φ_1*k*_ is the ENV vector (standardized between −1 and 1) at the value *k*, *a*_*ol*_ and *a*_1*l*_ are the random regression coefficients for the intercept and slope of the additive genetic effect of individual *l*, *p*_*m*_ is the random permanent environmental effect for sow *m* and *e*_*ijklmn*_ is the random residual error for record *n*. The following assumptions with regards to the additive genetic effects were made: $\left[\begin{array}{l} a_{0}\\ a_{1} \end{array}\right]$ ~ *N* (0, **H** ⊗ **G**). *G* is a 2 × 2 (co)variance matrix for the intercept and slope effects: $\text{G}=\left[\begin{array}{cc} \sigma_{0}^{2} & \sigma_{01}\\ \sigma_{10} & \sigma_{1}^{2} \end{array}\right]$, where $\sigma_{0}^{2}$ is the additive genetic variance for the intercept term, $\sigma_{1}^{2}$ is the additive genetic variance for the slope term, σ_10_ (and σ_01_) is the covariance between the two aforementioned effects. The contemporary group effect was defined only based on sow birth year to account for genetic trend while avoiding collinearity with the ENV effect.

The **H** matrix was constructed using the preGSf90 software (Aguilar et al., 2014) by blending the pedigree-derived (traced back for 10 generations) relationship matrix (**A**) and a SNP-derived genomic relationship matrix (Legarra et al., 2014):

$$H^{-1}=A_{11}^{-1}+\left[\begin{array}{cc} 0 & 0\\ 0 & ZD{Z^{\prime }}^{-1}-A_{22}^{-1} \end{array}\right]$$

where *ZDZ*^′−1^ is the inverse of the SNP-derived genomic relationship matrix, calculated based on the second method described by VanRaden (2008), $A_{22}^{-1}$ and $A_{11}^{-1}$ are the inverse of the **A** matrix for the genotyped and non-genotyped animals, respectively. The sow permanent environmental effect was assumed as $p_{m}=N\left(0,\text{I}⊗\sigma_{pe}^{2}\right)$, while residuals were allocated to five classes of residual variance:

$$\begin{array}{l} \left[\begin{array}{c} e_{n1}\\ e_{n2}\\ \begin{array}{c} e_{n3}\\ e_{n4}\\ e_{n5} \end{array} \end{array}\right]N\left(0,\text{I}⊗\text{R}\right),\text{where},\text{R}=\left[\begin{array}{c} \begin{array}{cc} \begin{array}{cc} \sigma_{e1}^{2} & 0 \end{array} & \begin{array}{ccc} 0 & 0 & 0 \end{array} \end{array}\\ \begin{array}{cc} 0 & \begin{array}{ccc} \sigma_{e2}^{2} & 0 & \begin{array}{cc} 0 & 0 \end{array} \end{array} \end{array}\\ \begin{array}{c} \begin{array}{cc} 0 & \begin{array}{ccc} 0 & \sigma_{e3}^{2} & \begin{array}{cc} 0 & 0 \end{array} \end{array} \end{array}\\ \begin{array}{ccc} 0 & 0 & \begin{array}{ccc} 0 & \sigma_{e4}^{2} & 0 \end{array} \end{array}\\ \begin{array}{ccc} 0 & 0 & \begin{array}{ccc} 0 & 0 & \sigma_{e5}^{2} \end{array} \end{array} \end{array}\\ \; \end{array}\right], \end{array}$$

And $\sigma_{et}^{2}$ is the residual variance for the *t*^*th*^ class. Phenotypic records were allocated to one of the five classes using the first four quintiles as discriminants, in order to have a balanced number of observations in each class. Variance components were estimated using the gibbs3f90 package from the BLUPF90 family programs (Misztal et al., 2018). Posterior means for the variance components and the genetic parameters were stored at each iteration. The variance component estimation was performed in two steps. First, all possible “trait x population x ENV” combinations were run, in order to find the ENV yielding the strongest GxE estimate as measured by the parameter $\sigma_{1}^{2}$. In this step, 30,000 iterations were run, and only genotypes of sires were included. While not providing an accurate estimate of variance components for GxE, we assumed this strategy to be effective in detecting the presence of GxE while saving computation time. The omission of sows' genotypes made the sampling process considerably faster. The second step consisted of analyzing the selected “trait x population x ENV” combinations, running 120,000 iterations with 20,000 iterations of burn-in and thinning every 20 rounds. All genotyped individuals were included in the analyses. The convergence was checked by visual inspection of trace plots and through the Geweke's test. After obtaining the random-regression variance components, an additive genetic (co)variance matrix among values of ENV **Γ** was calculated as:

$$\begin{array}{l} \Gamma =\Phi^{\text{′}}\text{G}\Phi \end{array}$$

Where **G** is the estimated (co)variance matrix between the intercept and slope terms (described above), Φ is a matrix of number of rows equal to the number of unique values of the ENV and two columns (a vector of “1” and the ENV). Heritability of each single value *k* of ENV ($h_{k}^{2}$) was calculated as follows:

$$\begin{array}{l} {\text{h}}_{\text{k}}^{2}=\frac{\Gamma_{\text{kk}}}{\Gamma_{\text{kk}}+\sigma_{\text{pe}}^{2}+\;\sigma_{\text{et}}^{2}}, \end{array}$$

Where Γ_*kk*_ is the *k*^*th*^ value of the diagonal of **Γ**, $\sigma_{pe}^{2}$is the estimated sow permanent environmental variance, and $\sigma_{et}^{2}$ is the estimate of residual variance for the respective ENV class (1 to 5) of each k^th^ value. Additive GxE predicted values at the population and individual level (i.e., reaction norms), were calculated as *r*_*k*_ = α1+βφ_1*k*_ and $r_{kl}={\left(\alpha +a_{0l}\right)}^{*}1+{\left(\beta {+a}_{1l}\right)}^{*}\varphi_{1k},$ where *r*_*k*_ is the predicted value *k* at the population level, and *r*_*kl*_ is the predicted value *k* for the individual *l*, and all other parameters are as described before.

### Genome-Wide Association Study and Functional Annotation

A GWAS was carried out for each of the three reproductive traits, performed on the population that showed the largest estimate of $\sigma_{1}^{2}$ in the second step of the variance component estimation. This approach was used in order to reduce the amount of results presented still delivering the most valuable findings. Marker effects were obtained by back-solving the gEBV (Wang et al., 2012). In brief, gEBV were calculated using the blupf90iod2 package (Misztal et al., 2018). Subsequently, the marker effects were obtained for both intercept and slope terms of the random regression model, using the postGSf90 software (Aguilar et al., 2014). Similarly to Wang et al. (2014) and Bergamaschi et al. (2020), direct genomic values for 10-SNP overlapping windows were calculated, their variance was divided by the whole-genome direct genomic values' variance and subsequently used as measure of relative contribution of that genomic region to the total genomic variance. Miami plots of the significant regions were generated using the ggplot2 package (Wickham, 2016) available in the R software. Genomic windows were selected if falling within the top 1% for contribution to genomic variance for the intercept and slope terms, independently. Genomic windows were then tested using a bootstrapping method following Howard et al. (2015) and Bergamaschi et al. (2020).

The genomic windows that were selected and passed the bootstrapping test were used for the subsequent functional genomic analyses. Firstly, the biomaRt package (Durinck et al., 2005, 2009) was used to retrieve candidate genes overlapping with the genomic windows identified, based on the current gene annotations from the ENSEMBL Genes platform (Version 99; www.ensembl.org/index.html). Subsequently, biological processes, metabolic pathways, and enrichment analyses were performed using the DAVID 6.8 (Huang et al., 2009a,b), the Kyoto Encyclopedia of Genes and Genomes—KEGG (Kanehisa et al., 2014) bioinformatic tools and the PANTHER Classification System (Thomas et al., 2003). Furthermore, the genes identified were compared to previously-published QTL regions using the Pig QTL database (www.animalgenome.org/cgi-bin/QTLdb/SS/index).

## Results

### Descriptive Statistics

Table 2 shows the descriptive statistics for the three reproductive traits in the four populations studied. The population with the largest number of records was SPG_LW, followed by SPG_LR and TML_LR (depending on the trait) and TML_YS. All populations had an average TNB and NBA above 10 and 9 piglets, respectively. AWB was equal or larger than 1.5 kg in all populations. Standard deviation values were similar among populations, although TML populations showed more variability.

**Table 2:** Descriptive statistics for each trait by population combination.

**Trait**	**Population**	**N**	**N sows**	**N sires**	**Mean**	**SD**
Total number of piglets born (TNB)	SPG_LR	21,104	11,113	886	11.94	3.53
	SPG_LW	27,616	12,155	739	12.48	3.94
	TML_LR	23,848	11,173	447	10.84	3.79
	TML_YS	11,230	5,179	249	11.09	3.92
Number of piglets born alive (NBA)	SPG_LR	21,104	11,113	886	10.86	3.40
	SPG_LW	27,631	12,138	739	11.26	3.83
	TML_LR	23,848	11,173	447	9.78	4.06
	TML_YS	11,229	5,178	249	9.81	4.29
Average birth weight (ABW)	SPG_LR	18,942	10,673	883	1.60	0.26
	SPG_LW	24,399	11,587	707	1.52	0.25
	TML_LR	14,521	8,262	401	1.56	0.29
	TML_YS	7,369	4,142	229	1.50	0.28

*Population: indicates the breeding company and breed, in which SPG, Smithfield Premium Genetics; TML, The Maschhoffs; LR, Landrace; LW, Large White; and YS, Yorkshire (also known as Large White in other breeding companies)*.

### Selection of Environmental Covariates

Table S1 shows a summary for the ENV tested in this study across the four populations studied. Table 3 presents the descriptive statistics for the ENV selected for each trait by population combination together with the variance components estimated for the additive genetic terms (intercept and slope) in the RRM. RH was the ENV selected for seven out of the 12 “ENV x trait x population” combinations. RH recorded from 14 to 7 days before conception was selected for both TNB and NBA in SPG_LR, while RH measured in the week before conception was selected for TNB and ABW in SPG_LW.

**Table 3:** Variance components estimates for the intercept and slope terms of the additive genetic effect.

**Trait**	**Pop**.	**Covariate**				**Variance components estimates**		
		**Variable and time of recording**	**Intercept**	**Min**	**Max**	**$\sigma_{0}^{2}$**	**σ_01_**	**$\sigma_{1}^{2}$**
TNB	**SPG_LR**	**Average Rel. Humidity 14 to 7 days before conception**	**52.50**	**11.74**	**93.24**	**8.93** ^**(7.51;10.26)**^	**0.61** ^**(−0.36;1.76)**^	**6.57** ^**(5.21;8.26)**^
	SPG_LW	Average Rel. Humidity 7 days to conception	52.50	11.74	93.24	9.99 ^(8.60;11.40)^	0.22 ^(−0.59;0.96)^	3.68 ^(2.76;4.61)^
	TML_LR	Maximum Temp. 28 to 35 days int pregnancy	15.07	−8.54	38.68	13.70 ^(11.90;15.66)^	−2.46 ^(−3.57;−1.38)^	4.69 ^(3.49;5.87)^
	TML_YS	Maximum Temperature 14 to 21 days into pregnancy	16.81	-6.46	40.14	10.34 ^(8.49;12.18)^	−2.01 ^(−3.03;−1.01)^	3.71 ^(2.83;4.57)^
NBA	SPG_LR	Average Rel. Humidity 14 to 7 days before conception	52.50	11.74	93.24	9.35 ^(7.97;10.74)^	0.72 ^(−0.20;1.72)^	4.93 ^(3.86;6.20)^
	SPG_LW	Average Rel. Humidity 100 to 107 days into pregnancy	51.78	11.74	91.82	10.73 ^(9.38;12.23)^	−0.81 ^(−1.70;0.04)^	4.04 ^(3.13;5.03)^
	**TML_LR**	**Average THI 86 to 93 days into pregnancy**	**46.43**	**12.18**	**80.7**	**14.63** ^**(12.58;16.65)**^	**-2.99** ^**(−4.35;−1.58)**^	**7.44** ^**(5.80;9.23)**^
	TML_YS	Minimum Temp. 72 to 79 days into pregnancy	2.22	−18.47	22.92	10.62 ^(8.78;12.48)^	−2.70 ^(−3.91;−1.41)^	6.33 ^(4.91;7.79)^
ABW	SPG_LR	Max Temp. 21 to 14 days before conception	19.58	−0.69	39.86	26.09 ^(23.71;28.51)^	−0.46 ^(−1.65;0.70)^	3.46 ^(2.73;4.24)^
	SPG_LW	Average Rel. Humidity 7 days to conception	52.50	11.74	93.24	23.11 ^(21.09;25.29)^	0.01 ^(−1.40;1.30)^	4.80 ^(3.71;5.92)^
	TML_LR	Average Rel. Humidity 14 to 21 days into pregnancy	59.09	27.65	90.55	30.90 ^(28.05;33.98)^	−3.25 ^(−5.17;−1.31)^	8.25 ^(6.32;10.22)^
	**TML_YS**	**Average Rel. Humidity 107 to 114 days into pregnancy**	**59.09**	**27.65**	**90.55**	**28.60** ^**(24.98;32.32)**^	**−2.33** ^**(−4.76;−0.18)**^	**9.40** ^**(7.01;11.78)**^

*Results are reported for each trait by population combination, with one case representing the largest estimate of the slope term variance. Cases highlighted in bold show the strongest estimate of the slope term variance, per trait*.

*Pop., indicates the breeding company and breed, in which SPG, Smithfield Premium Genetics; TML, The Maschhoffs; LR, Landrace; LW, Large White; and YS, Yorkshire (also known as Large White in other breeding companies). Covariate: indicates the environmental covariate selected for the population-trait combination, mean and SD are reported. Intercept, indicates the value that was set to 0 (intercept) in the random regression model*.

RH in similar gestation periods (100 to 107 days, and 107 to 114 days into pregnancy, for NBA in SPG_LW and ABW in TML_YS, respectively) was selected. Also, RH in the third week of gestation was selected for ABW in TML_LR. In addition, Max.T was selected for TNB (in the fifth and third week of gestation in TML_LR and TML_YS, respectively) and ABW in SPG_LR (from 21 to 14 days before conception). For NBA, average THI recorded from 86 to 93 days into pregnancy was selected in TML_LR while Min.T recorded from 72 to 79 days into pregnancy was selected in TML_YS. The variance components estimates were partially consistent within trait and across populations. The proportion of variance explained by the intercept term ($\sigma_{0}^{2}$) was smaller for TNB (8.93 to 10.34) and NBA (9.35 to 14.63) and greater for ABW (23.11 to 30.90), and consistently larger for TML populations (with TML_LR showing the largest values). The proportion of variance explained by the slope term ($\sigma_{1}^{2}$) was significantly smaller than the intercept term (3.46 to 9.40 vs. 8.93 to 30.90). The covariance term σ_01_ (off-diagonal element of covariance matrix **G**) did not include the value 0 in the confidence intervals only for the TML populations. In these populations the estimates ranged from −3.25 to −2.01, with larger values for TML_LR and TML_YS. Further results will be reported only for the scenarios with the largest estimate of $\sigma_{1}^{2}$: (1) SPG_LR for TNB regressed on RH measured 14 to 7 days before conception, (2) TML_LR for NBA regressed on THI measures 89 to 93 days into pregnancy, and, (3) TML_YS for ABW regressed on RH measured 107 to 114 days into pregnancy.

### Heritability Estimates

**Figure 1:**
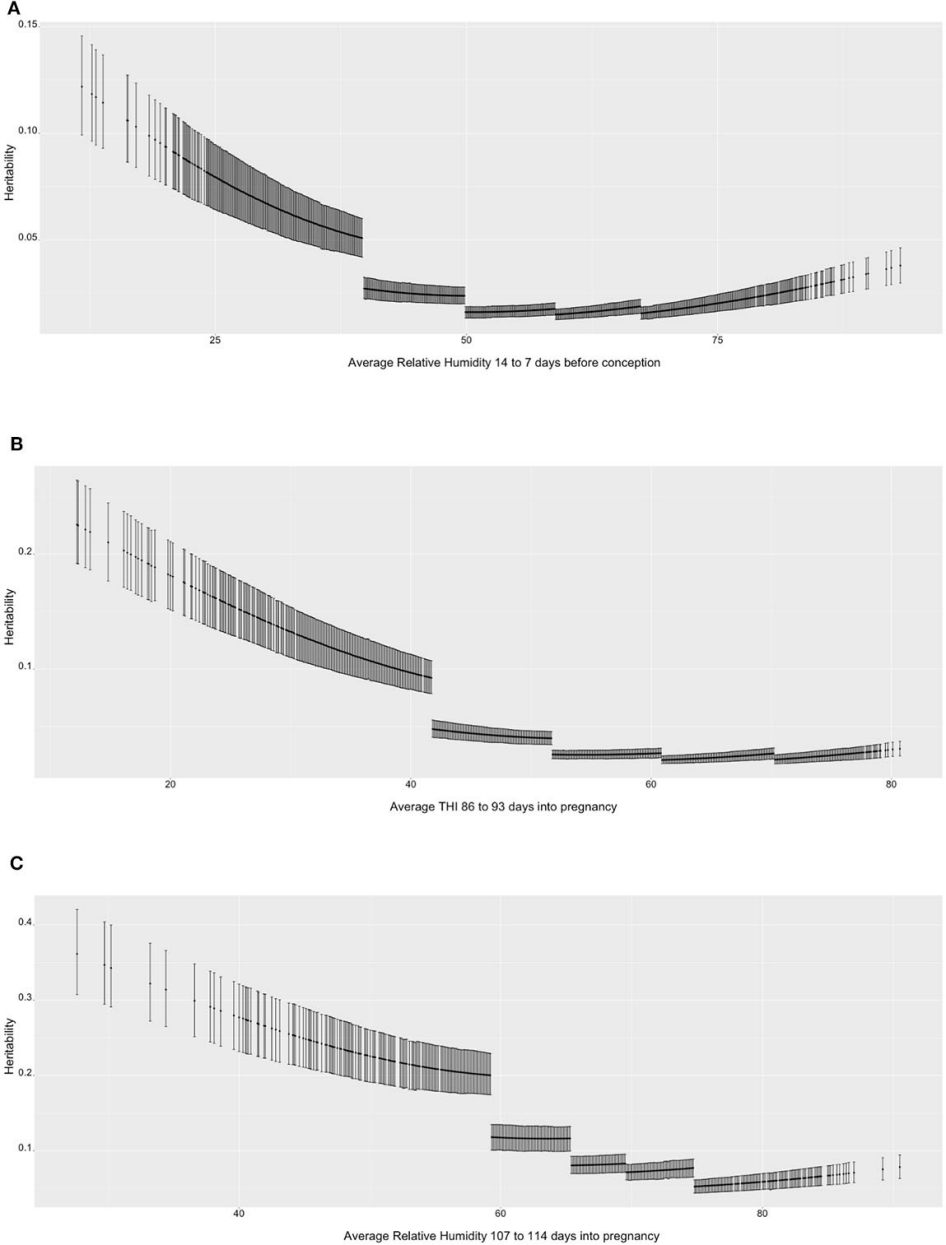
**(A)** Heritability estimates (posterior means with 95% empirical confidence intervals) for total number of piglets born (TNB) in the Smithfield Premium Genetics Landrace population (SPG_LR) over the range of Average Relative Humidity 14 to 7 days before conception. **(B)** Heritability estimates (posterior means with 95% empirical confidence intervals) for number of piglets born alive (NBA) in the Landrace population of the Maschhoffs breeding company (TML_LR) over the range of average THI 86 to 93 days into pregnancy. **(C)** heritability estimates (posterior means with 95% empirical confidence intervals) for average birth weight (ABW) in the Yorkshire population of the Maschhoffs breeding company (TML_YS) over the range of average relative humidity 107 to 114 days into pregnancy.

Figure 1 shows the heritability estimates across the range of ENV for the three selected scenarios. Figure 1A presents the heritability estimates for TNB over value of RH. In general, there is a decrease in heritability as RH increases and the environmental conditions become more uncomfortable to the sows. The decrease in heritability is mostly due to the increase in residual variance for the high-RH classes, as it can be seen that within-class the trend is stable, with 0.05 to 0.12 values in the high-RH class and a 0.02 to 0.04 values in the low-RH class. The heritability estimates ranged from 0.05 to 0.10 for low-RH conditions and was stable below 0.05 under the other environmental conditions. Similar results were observed for NBA (Figure 1B). The heritability estimates are larger (between 0.1 and 0.2) in the low range of THI, but lower (~0.05) and stable in all other THI classes. For ABW (Figure 1C), the heritability estimates are larger compared to the TNB and NBA but follow a similar trend (highest values in the most comfortable environmental gradients and descending values as RH surpasses 60%).

### Reaction Norms and Re-ranking Across Environments

**Figure 2:**
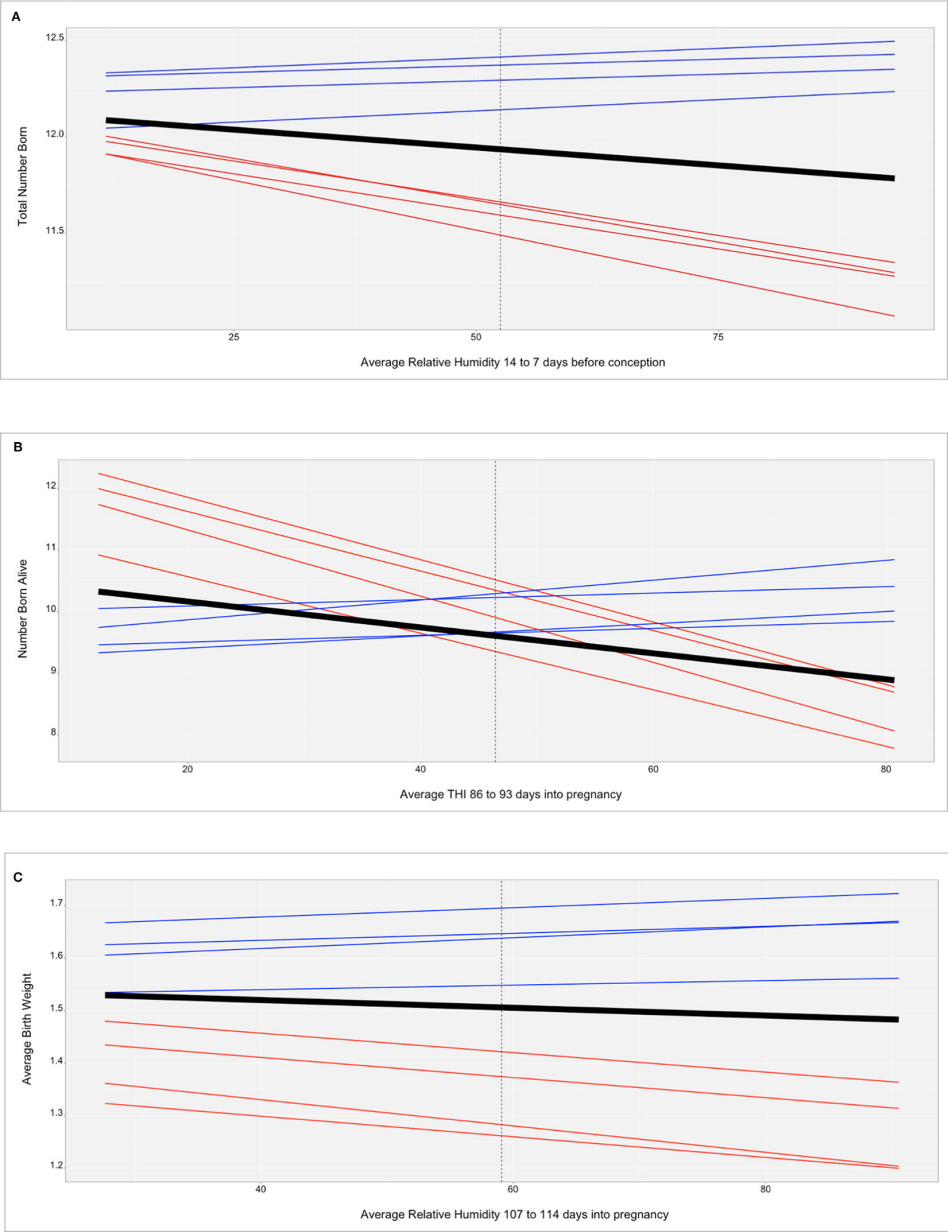
**(A)** Reaction norms for total number of piglets born (TNB) in the Smithfield Premium Genetics Landrace population (SPG_LR) as regressed on Average Relative Humidity 14 to 7 days before conception. Black line indicates the population trend, blue lines indicate the most tolerant sires, red line indicates the most susceptible sires. **(B)** Reaction norms for number of piglets born alive (NBA) in the Landrace population of the Maschhoffs breeding company (TML_LR) as regressed on average THI 86 to 93 days into pregnancy. Black line indicates the population trend, blue lines indicate the most tolerant sires, red line indicates the most susceptible sires. **(C)** Reaction norms for average birth weight (ABW) in the Yorkshire population of the Maschhoffs breeding company (TML_YS) as regressed on average relative humidity 107 to 114 days into pregnancy. Black line indicates the population trend, blue lines indicate the most tolerant sires, red line indicates the most susceptible sires.

**Figure 3:**
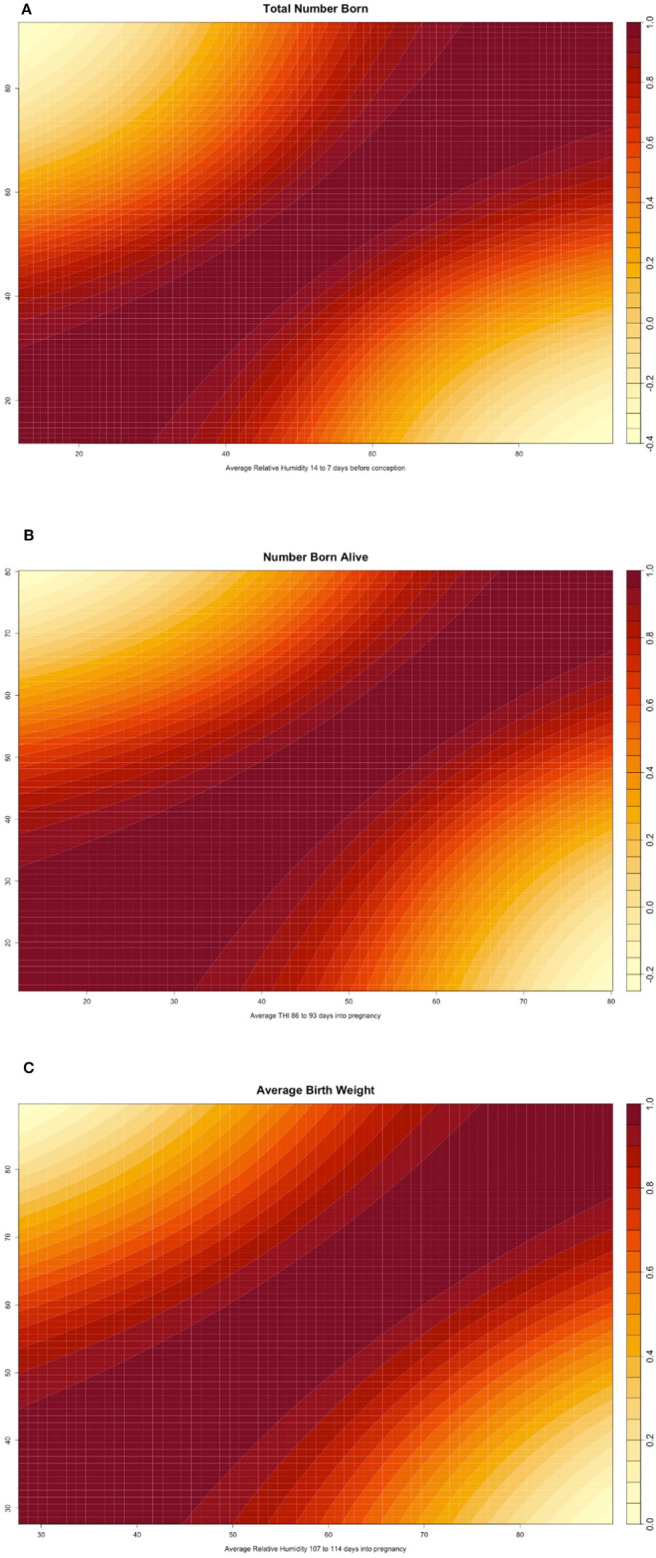
**(A)** Heatmap reporting the genetic correlation estimates (posterior mean) across values of the environmental covariate for total number of piglets born (TNB) in the Smithfield Premium Genetics Landrace population (SPG_LR) as regressed on Average Relative Humidity 14 to 7 days before conception. **(B)** Heatmap reporting the genetic correlation estimates (posterior mean) across values of the environmental covariate for number of piglets born alive (NBA) in the Landrace population of the Maschhoffs breeding company (TML_LR) as regressed on average THI 86 to 93 days into pregnancy. **(C)** Heatmap reporting the genetic correlation estimates (posterior mean) across values of the environmental covariate for average birth weight (ABW) in the Yorkshire population of the Maschhoffs breeding company (TML_YS) as regressed on average relative humidity 107 to 114 days into pregnancy.

Figure 2 shows the genomic reaction norms for each population average (black tick lines) and top and bottom HT boars (sires), for the three selected scenarios. Sires were selected to have at least 50 daughters so that the phenotypes were distributed under the largest range in ENV (i.e., daughters' records could not be concentrated under the comfortable environmental gradients). Under the three studied cases, this resulted in 28 sires for SPG_LR, 54 sires for TML_LR and 18 sires for TML_YS. However, only reaction norms for the four most HT and the four most heat susceptible sires are reported, in blue and red, respectively. In the case of TNB (Figure 2A), there is an unfavorable effect of HS across the ENV range, with a difference of 0.25 piglets from the driest to the most humid conditions (from 12 to 11.75 piglets). While there are not relevant differences between the groups of sires in the driest environment, heat susceptible sires show a stronger reduction in reproductive performance of approximately 1 piglet across the whole ENV range. HT sires had no (or small) reduction in reproductive performance. For TNB, off-diagonal values of the genetic correlation matrix **Γ** showed a median value of 78.7% and a minimum value of −37.8% (Figure 3A), supporting a remarkable GxE effect. For NBA (Figure 2B), there were also clear negative effects in reproductive performance [almost 1.5 less piglets born alive from low THI values (around 10 points) to the highest values (80 points)]. Larger variability was also seen among the sires' reaction norms, with sensitive sires generally showing higher performance under comfortable environmental conditions. Some HT sires appeared to have an increased performance in high-THI compared to low-THI conditions, but the differences were small. However, HT sires in the uncomfortable environments do not outperform the sensitive sires in the comfortable environmental gradients. For NBA, the genetic correlations in **Γ** indicated a more moderate GxE effect, with a median value of 83.4% and a minimum value of −21.7% (Figure 3B). Figure 2C presents the reaction norms for ABW. While the population average reduction in ABW across the range of RH is negligible, the sires' reaction norms show a group of high HT sires that also have the best performance in the low-RH conditions, with a small reduction in ABW (~0.1 kg). For ABW, the genetic correlations in **Γ** showed the most moderate GxE effect, with a median value of 87.6% and minimum value of 0% (Figure 3C).

### Genome-Wide Association Study and Functional Annotation

**Figure 4:**
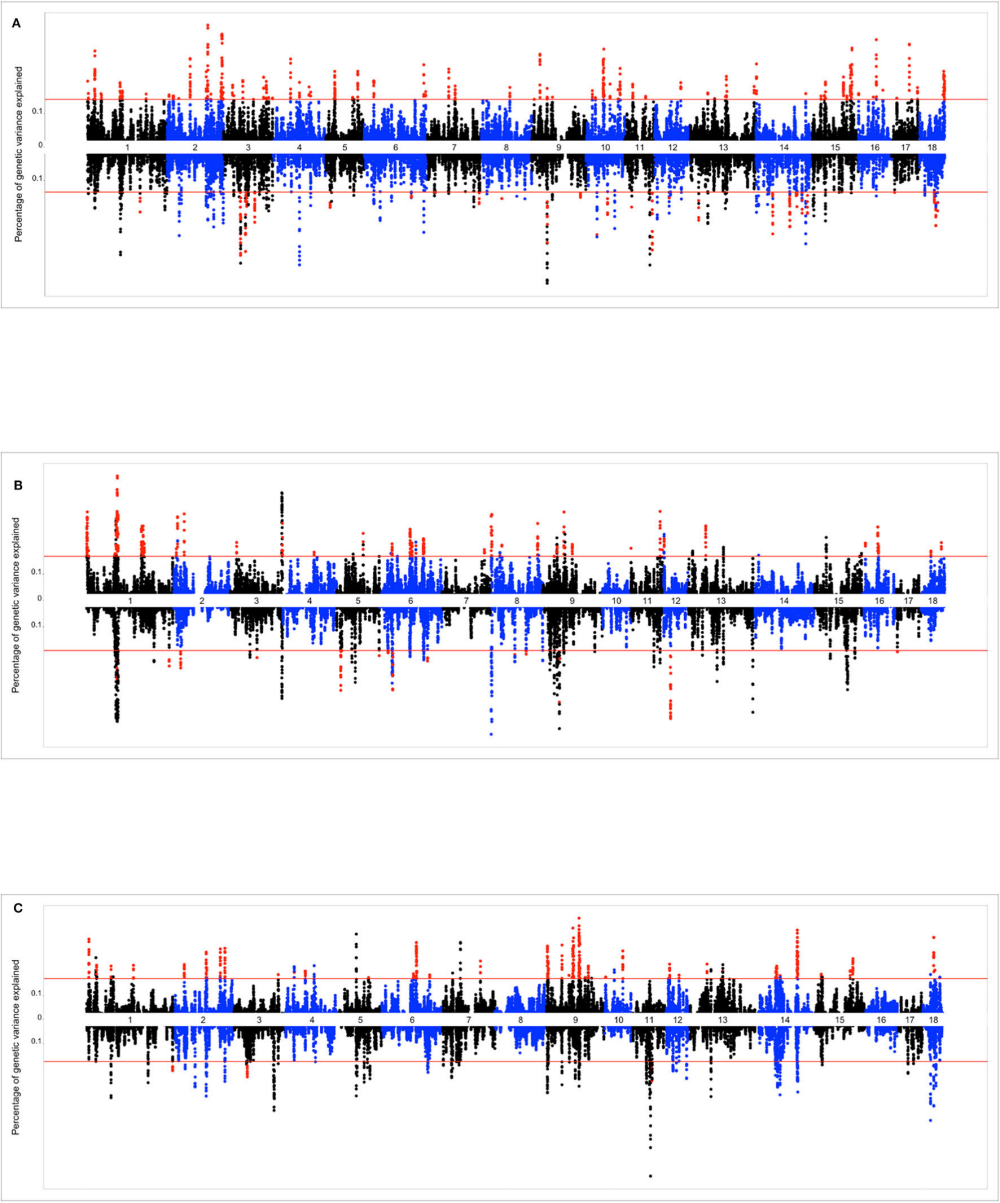
**(A)** Miami plot (proportion of genomic variance explained by each 10-SNP window) for total number of piglets born (TNB) in the Smithfield Premium Genetics Landrace population (SPG_LR) as regressed on Average Relative Humidity 14 to 7 days before conception. Graph above the line reports the variance absorbed for the intercept term of the random regression model (across-environment performance), graph below the line reports variance absorbed for the slope term (reaction norms). **(B)** Miami plot (proportion of genomic variance explained by each 10-SNP window) for number of piglets born alive (NBA) in the Landrace population of the Maschhoffs breeding company (TML_LR) as regressed on average THI 86 to 93 days into pregnancy. Graph above the line reports the variance absorbed for the intercept term of the random regression model (across-environment performance), graph below the line reports variance absorbed for the slope term (reaction norms). **(C)** Miami plot (proportion of genomic variance explained by each 10-SNP window) for average birth weight (ABW) in the Yorkshire population of the Maschhoffs breeding company (TML_YS) as regressed on average relative humidity 107 to 114 days into pregnancy. Graph above the line reports the variance absorbed for the intercept term of the random regression model (across-environment performance), graph below the line reports variance absorbed for the slope term (reaction norms).

The Miami plots presented in Figure 4 show the percentage of additive genetic variance explained by each 10-SNP overlapping genomic window for the intercept and slope terms of the genomic reaction norms based on TNB, NBA, and ABW. The suggestive red line highlights the genomic windows that were tested via bootstrapping, while red dots indicate the genomic windows that were considered as significant. The number (and percentage) of selected genomic windows for bootstrapping were 891 (1.84%), 700 (1.68%), and 702 (1.72%) for TNB, NBA, and ABW, respectively.

The top significant genomic windows for the slope term on TNB as regressed on average RH from 14 to 7 days prior to conception were located at SSC3, SSC9, and SSC11 (Table 4). The most significant genomic windows for the intercept term (shown in Figure 4A) were located on SSC2 (from 133,626,412 to 134,446,288 bp as well as from 157,492,614 to 158,466,521 bp), SSC16 (from 47,916,509 to 48,994,238 bp) and SSC17 (from 48,537,476 to 49,274,453 bp). Furthermore, 116 additional genomic windows explained a minimum of 0.13% of the total additive genetic variance for the intercept and slope terms on TNB (Tables S2a, S2b, respectively).

**Table 4:** The top significant genomic windows for the slope term of the reaction norm on total number of piglets born (TNB) as regressed on the Average Relative Humidity 14 to 7 days before conception in the SPG_LR population.

**Location**	**Var(%)**	**N SNP**	**Positional genes**	**QTL traits**
SSC3: 35,273,575–36,463,191	0.32	23	*ENSSSCG00000007917* (*RBFOX1*), *ENSSSCG00000051042*, *ENSSSCG00000046506*, *ENSSSCG00000020433*, *ENSSSCG00000051435*	CD8-negative leukocyte percentage, CD8-positive leukocyte percentage, CD3-negative
SSC3: 60,835,887–62,535,813	0.31	18	*ENSSSCG00000035540*	Feed conversion ratio, back fat thickness between 3rd and 4th rib, ham weight, CD8-positive leukocyte percentage, CD3-negative, CD8-negative leukocyte percentage, average daily gain, Japanese color scale, meat color L
SSC11: 80,629,582–81,387,249	0.31	19	-	Conductivity 45 min *post-mortem*, hind leg conformation
SSC9: 28,682,617–29,350,733	0.28	17	*ENSSSCG00000039906*, *ENSSSCG00000020347*	Litter weight, adrenal gland weight, hind-leg conformation, gestation length

*Location, indicates the chromosome number (SSC1 to SSC18), followed by the starting and end position of the genomic window(s) in base pairs; Var(%), the percental variance explained by the most relevant 10-SNP window within the region of interest; N SNP, number of SNP marker included in the genomic region identified; Positional genes, Ensembl gene stable ID (with official gene symbol when known) mapped to the genomic regions identified; QTL traits, indicates which traits have known QTLs mapped within the genomic region of interest, based on QTLs reported in the Pig QTL database (https://www.animalgenome.org/cgi-bin/QTLdb/SS/index)*.

The top significant genomic windows for the slope term on NBA as regressed on average THI from 86 to 93 days into pregnancy were located in SSC5, SSC6, SSC9, and SSC12 (Table 5). The most significant genomic windows for the intercept term (shown in Figure 4B) are located at SSC1 (from 266,158 to 2,322,507 bp and from 67,951,385 to 71,151,381 bp), SSC11 (from 79,931,895 to 80,427,841 bp) and SSC9 (from 43,064,592 to 43,794,001 bp). The genomic window located on SSC9 (28,988,041 to 29,535,913 bp) with strong impact on NBA partially overlaps with another genomic window located on SSC9 (28,682,617 to 29,350,733 bp), with moderate effect on the slope term for TNB, despite of the fact that the association study was performed on different populations. Furthermore, 54 additional genomic windows explaining more than 0.17% of the total additive genetic variance were identified for the intercept and slope term on NBA (Tables S3a, S3b). These regions were located on all chromosomes, except SSC10 and SSC14.

**Table 5:** Top four significant windows for the slope term on NBA as regressed on average THI 86 to 93 days into pregnancy in the TML_LR population.

**Location**	**Var(%)**	**N SNP**	**Positional genes**	**QTL traits**
SSC12: 15,953,277–17,487,606	0.45	32	*ENSSSCG00000017299* (*MARCH10*), *ENSSSCG00000017300* (*MRC2*), *ENSSSCG00000017301* (*TLK2*), *ENSSSCG00000033421*, *ENSSSCG00000017304* (*EFCAB3*), *ENSSSCG00000051573*, *ENSSSCG00000045518*, *ENSSSCG00000047329*, *ENSSSCG00000049121*, *ENSSSCG00000017305*, *ENSSSCG00000017306*, *ENSSSCG00000017307* (*MYL4*), *ENSSSCG00000017308* (*CDC27*), *ENSSSCG00000017310* (*KANSL1*), *ENSSSCG00000017311* (*MAPT*), *ENSSSCG00000043989*, *ENSSSCG00000017314* (*SPPL2C*), *ENSSSCG00000017313*, *ENSSSCG00000031893*, *ENSSSCG00000017316* (*NSF*), *ENSSSCG00000031561*	Red blood cell count, hematocrit, loin muscle area, loin pH 24 h *post-mortem*
SSC9: 28,988,041–29,535,913	0.39	10	*ENSSSCG00000039906*, *ENSSSCG00000020347*, *ENSSSCG00000048478*	Litter weight, hind-leg conformation
SSC5: 5,425,963–5,866,100	0.34	21	*ENSSSCG00000038685* (*MPPED1*), *ENSSSCG00000051106*, *ENSSSCG00000000029* (*SCUBE1*), *ENSSSCG00000000034* (*TTLL12*), *ENSSSCG00000000033*, *ENSSSCG00000000031* (*MCAT*), *ENSSSCG00000042788*, *ENSSSCG00000046130*, *ENSSSCG00000000035* (*TTLL1*), *ENSSSCG00000048701*, *ENSSSCG00000000036* (*PACSIN2*)	Hind-feet conformation, corpus luteum number
SSC6: 23,525,525–23,900,710	0.34	16	*ENSSSCG00000047100*, *ENSSSCG00000018823*, *ENSSSCG00000048963*, *ENSSSCG00000050597*, *ENSSSCG00000047287*	Obesity index, gestation length, eicosadienoic acid content, 60-day body weight

*Location, indicates the chromosome number (SSC1 to SSC18), followed by the starting and end position of the genomic window(s) in base pairs; Var(%), the percental variance explained by the most relevant 10-SNP window within the region of interest; N SNP, number of SNP marker included in the genomic region identified; Positional genes, Ensembl gene stable ID (with official gene symbol when known) mapped to the genomic regions identified; QTL traits, indicates which traits have known QTLs mapped within the genomic region of interest, based on QTLs reported in the Pig QTL database (https://www.animalgenome.org/cgi-bin/QTLdb/SS/index)*.

The top significant genomic windows for the slope term on ABW as regressed on average RH from 107 to 114 days into pregnancy were located on SSC1, SSC3, and SSC11 (Table 6). The most significant genomic windows for the intercept term of ABW (Figure 4C) are located in SSC9 (from 75,823,479 to 81,214,756 and from 55,824,884 to 58,228,279 bp), SSC14 (from 114,610,574 to 117,846,180 bp) and SSC18 (from 31,560,363 to 32,946,579 bp). Furthermore, 44 additional genomic windows explaining more than 0.18% of the total additive genetic variance for the intercept term on ABW were identified (Table S4a).

**Table 6:** Significant windows for the slope term on ABW as regressed on average relative humidity 107 to 114 days into pregnancy in the TML_YS population.

**Location**	**Var(%)**	**N SNP**	**Positional genes**	**QTL traits**
SSC11: 55,037,726–55,844,999	0.26	14	*ENSSSCG00000035248*, *ENSSSCG00000042294*, *ENSSSCG00000047575*, *ENSSSCG00000044970*, *ENSSSCG00000042996*	Age at puberty
SSC3: 25,794,656–26,553,194	0.24	15	*ENSSSCG00000007864* (*GPRC5B*), *ENSSSCG00000034720* (*IQCK*), *ENSSSCG00000025203* (*KNOP1*), *ENSSSCG00000027324* (*VPS35L*), *ENSSSCG00000047672*, *ENSSSCG00000023829* (*CCP110*), *ENSSSCG00000029212* (*GDE1*), *ENSSSCG00000007868* (*TMC5*), *ENSSSCG00000018432*, *ENSSSCG00000007866* (*TMC7*), *ENSSSCG00000034655* (*COQ7*), *ENSSSCG00000048910*, *ENSSSCG00000039337* (*ITPRIPL2*), *ENSSSCG00000042386*, *ENSSSCG00000045906*, *ENSSSCG00000022200* (*SYT17*), *ENSSSCG00000047938* (*CLEC19A*)	CD8-negative leukocyte percentage, cortisol level, maternal infanticide, loin pH 45 min *post-mortem*
SSC1: 309,786,519–310,467,666	0.21	13	-	-

*Location, indicates the chromosome number (SSC1 to SSC18), followed by the starting and end position of the genomic window(s) in base pairs; Var(%), the percental variance explained by the most relevant 10-SNP window within the region of interest; N SNP, number of SNP marker included in the genomic region identified; Positional genes, Ensembl gene stable ID (with official gene symbol when known) mapped to the genomic regions identified; QTL traits, indicates which traits have known QTLs mapped within the genomic region of interest, based on QTLs reported in the Pig QTL database (https://www.animalgenome.org/cgi-bin/QTLdb/SS/index)*.

The positional candidate genes identified are presented in Tables 4–6, Tables S2a, S2b, S3a, S3b, S4a. The genes and genomic regions identified to be associated with heat tolerance (slope term) have been previously linked to a variety of trait groups: body temperature, blood-related traits (e.g., hemoglobin content, white blood cell number, leukocyte percentage), coping behavior, organ weight and size (adrenal gland, liver, head, kidney, heart, small intestine), melanoma susceptibility, maternal infanticide, growth rate, meat quality, carcass fatness, and fatty acid metabolism, fertility, thoracic vertebra number, water holding capacity (and drip loss), and *Salmonella* shedding status (Tables 4–6, Tables S2b, S3b). The most significant (*p*-value < 0.05) KEGG pathway identified was: “ssc00061:Fatty acid biosynthesis.” The main biological processes identified (*p*-value < 0.05 after multiple testing correction) were: cardiac conduction system development, ventricular cardiac muscle tissue development, genitalia development, epithelium development, embryonic forelimb morphogenesis, tube development, and somatic diversification of immune receptors via germline recombination within a single locus. The significant (*p*-value < 0.05 after multiple testing correction) cellular components associated with the candidate genes identified are: intermediate filament cytoskeleton, supramolecular fiber, cytoskeleton, non-membrane-bounded organelle, and intracellular part. The significant (*p*-value < 0.05 after multiple testing correction) metabolic functions identified are structural molecular activity and core promoter binding. Furthermore, Figures S3–S5 present a schematic representation of the functional analyses of the intercept term using the PANTHER Classification scheme.

The genes and genomic regions identified to be associated with the intercept term have been previously linked to a variety of trait groups, including: spinal curvature, growth traits, meat fatty acid composition, meat quality, gestation length, NBA, testicular width and length, semen pH, number of mummified piglets, glucose level, age at puberty, gait score, corpus luteum number, litter size, number of non-viable fetuses, skin thickness, time spent lying, maternal infanticide, body length, chest circumference, birth weight variability, cortisol level, piglet mortality, teat number, and scrotal hernia (Tables S2a, S3a, S4a). The most significant (*p*-value < 0.05) KEGG pathways identified were: “Oocyte meiosis,” “Insulin resistance,” “Neutrophin signaling pathway,” “Insulin signaling pathway,” “Basal transcription factors,” and “Acute myeloid leukemia.” The biological processes identified (*p*-value < 0.05 after multiple testing correction) were: regulation of cellular response to stress, response to starvation, regulation of cell proliferation, cellular response to nutrient levels, hemoglobin metabolic process, mitotic cell cycle response, macromolecule modification, regulation of catabolic process, regulation of protein metabolic process, regulation of kinase activity, apoptotic signaling pathway, regulation of phosphate metabolic process, DNA methylation or demethylation, cellular response to extracellular stimulus, regulation of immune system process, and animal organ development. Figures S6–S8 present a schematic representation of the functional analyses for the slope term using the PANTHER Classification System. Despite of the use of different databases, similar pathways and biological processes were identified based on DAVID or PANTHER.

## Discussion

In the present study we performed a series of comprehensive analyses to reveal the genomic background of heat tolerance based on routinely-measured phenotypic records and publicly recorded weather variables, and provide the basic knowledge needed for implementation of genomic selection for heat tolerance in maternal-line swine breeds. We focused on four independent lines of sows to test the discoveries over genetically-different populations that were raised under different management systems, therefore also testing the portability of the methods employed in the study. We finally explored the genetic basis of heat tolerance for the three traits in selected populations. The use of four independent pig populations greatly validates the results identified and conclusions drawn in this study.

### Descriptive Statistics

The reproductive performance observed in the four populations (Table 2) are within the ranges reported in other studies (Bloemhof et al., 2008; Williams et al., 2013). LR sows farrowed relatively less piglets compared to the other breeds, but the ABW (per piglet born) was slightly higher. The overall reproductive performance of the TML populations is similar to SPG, but YS had greater ABW with lower prolificacy compared to LR. When compared to other studies investigating HT based on reproductive traits, TML and SPG had lower TNB and NBA values (Su et al., 2007; Wegner et al., 2014). However, similar prolificacy was reported in YS and LW sow populations raised in Spain (Bloemhof et al., 2008, 2013) and in purebred LR and LR x LW crossbred animals raised in the US (Williams et al., 2013). Therefore, the four populations used in the present study are representative of North-American and European swine breeding populations based on their prolificacy level.

### Selection of Environmental Covariates

In the present study, climate records from the National Climatic Data Center weather stations were used to describe the environmental conditions experienced by the sows at different stages of ovulation and pregnancy. While these climate records are usually publicly available and have been successfully employed in other worldwide studies focusing on sow HT (Tummaruk et al., 2010; Wegner et al., 2014), the collection of within-barn environmental measurements is an alternative to further improve the quality of these estimates for such studies. It would be advisable for breeders to record indoor environmental conditions for a more accurate assessment of heat stress.

The sow's thermoneutral zone has been estimated to be between 18 and 20 Celsius degree (Peltoniemi et al., 1999) and the distribution of values recorded by the weather stations by far exceeded those boundaries (Table 2). This suggests that, unless the farms employed very efficient methods to control the environmental conditions within the barns, HS was expected to have a large impact on the welfare and performance of the populations included in this study.

### Time of Recording of the Climate Variable to Define the Environmental Covariates

In this study we assumed that the impact of HS on reproductive performance can be reconduced to a specific time range from ovulation to farrowing. We believe this assumption has two advantages: (1) the possibility to discover a critical time in which the sows appear to show different tolerance to HS for at least one trait and (2) the ability to leverage on a larger variation in ENV, since averaging the environmental conditions found in a 114-d span could reduce the observed variability. As for point (1), in our data we found that the autocorrelation for ENV in consecutive weeks is relatively large (larger or equal to 0.9 for temperature and THI variables, larger than 0.65 for humidity) so that such step would be unnecessary in future analyses.

In general, HS occurring before conception or early pregnancy had a remarkable impact on prolificacy. For the SPG populations on both TNB and ABW, and for SPG_LR on NBA, the ENV covariates reported during ovulation and conception were selected to be the best environmental stressors to assess heat tolerance. The negative effect of pre-conception HS has been reported in other populations as well. For instance, Iida and Koketsu (2014) found a significant detrimental effect of summer conditions at conception on TNB. Tani et al. (2016) also reported that high temperature and humidity recorded on a 2-day window around farrowing decreased farrowing rate and increased number of stillborn piglets. Sevillano et al. (2016) showed that HS recorded 21 days before the sows' first insemination was detrimental to a successful farrowing. Early-pregnancy (first month) HS was also an important factor in the present study. On TML populations, TNB was affected by Max.T and ABW was affected by RH in the third week of pregnancy. HS during both the pre-conception and early pregnancy periods had a detrimental impact on TNB in a study conducted by Bloemhof et al. (2013), where the whole pre-conception to farrowing period was evaluated. In general, these results indicate that HS can alter the hypothalamic-hypophysial-ovarian axis, follicular and oocyte development, embryo implantation, and survival (Hansen et al., 2001; Bertoldo et al., 2012; De Rensis et al., 2017). Interestingly, the candidate genes and biological pathways identified for both the intercept and slope of the genomic reaction norms are related to these processes. In addition, examples of later pregnancy HS on ABW was observed for TML_YS, as well for NBA on three populations. While our results are not in total agreement with the strong impact of HS during ovulation and early pregnancy, it should be considered that these results do not deny the impact of HS in other time periods. In addition, it should be noted that late-pregnancy HS could have a detrimental impact on sow feed intake, with a compromising effect on fetus survival (and consequently, NBA) and fetus growth (and thus, ABW). In this context, Wegner et al. (2014) reported that NBA was affected by high temperatures and THI before farrowing.

Seven of the 12 ENV selected were built upon RH as climate variable, one was built upon THI and only four used temperature (with Min.T in one case and Max.T in three other scenarios). The preponderance of RH, rather than temperature, in challenging sows was also found by Tummaruk et al. (2010). This might indicate that the barn cooling systems can mitigate the impact of temperature better than RH.

### Variance Components Estimates

#### Heritability of Heat Tolerance

We used genomic random-regression models for the estimation of genetic parameters, including additive genetic variance for the trait performance in a standard environment ($\sigma_{0}^{2}$, intercept term) as well as the additive genetic variance for the tolerance to a given environmental stressor ($\sigma_{1}^{2},s$lope term). Variance components are expressed as percentage of total phenotypic variance for ease of comprehension (Table 3). The additive genetic variance estimate for the intercept term represents the genetic variance at the value of environmental covariates that is set to 0 in the random regression model and is also marked as a vertical line in Figures 2A–C. Additive genetic variance estimates are in agreement with the literature (e.g., Putz et al., 2015) and seem to be mostly determined by the specificities and phenotypic scale of each trait. In particular, the heritability for ABW is larger than the estimates for TNB and NBA. This is likely due to the ABW calculation, which takes the average performance over a full-sib, common environment group of animals, which likely led to the underestimation of the residual variance. On the other hand, the additive genetic variance for the slope term (HT indicator) does not show a clear pattern across traits and populations. First and foremost, this could be partially due to the use of different ENV for each analysis, though it is be expected that different populations (breeds) can have a different variability in terms of HT, i.e., the potential of selection for HT could be larger in one rather than another population (Bloemhof et al., 2013; Usui and Koketsu, 2015). In general, the variability observed indicates that genetic progress for improved HT can be achieved through selective breeding and its efficiency will depend on the accuracy of the phenotypes used. The identification of novel indicators of HT that better represent the physiological responses to HS will be paramount on improving the accuracies of genomic predictions for HT in swine.

#### Heritability Change Across the Range of Environmental Conditions

The parameters estimated using RRM can be used to provide further estimates of heritability across the observed values of ENV. Figure 1 shows the heritability estimates for the three selected trait scenarios. While the heritability estimates were larger for ABW than TNB and NBA, in general larger heritability estimates were observed at the low (comfortable) levels of the ENV. The changes in heritability derived more from different estimates of residual variance of the five classes of ENV, as shown by the broken line between class 1 (low values) and class 2 (medium-low values). No particular changes in heritability were observed between the other ENV classes. These results are in partial agreement with Lewis and Bunter (2011), who did not observe relevant differences in heritability estimates on TNB, NBA and ABW expressed under different year seasons. As for the higher heritability in comfortable conditions, Silva et al. (2014) reported higher heritability estimates for TNB in contemporary groups with better performance and Fragomeni et al. (2016b) found a similar trend when assessing HT based on body weight of purebred Duroc individuals.

#### GxE and Genetic Correlations Across Environments

GxE interaction can be confirmed, among other factors, by a genetic correlation lower than 0.70 across the range of ENV (Mulder and Bijma, 2007). In this study, genetic correlation for the three scenarios investigated reached median values above such threshold, but the minimum values were lower than the 0.70 threshold. Therefore, there is moderate GxE based on sow reproductive traits under different environmental conditions (presence or absence of HS). Similar values of genetic correlations across ENV were found by Lewis and Bunter (2011) on TNB, where the genetic correlation between spring and autumn performance was below 0.70 and by Fragomeni et al. (2016b) on crossbred growth performance, where the minimum genetic correlation was close to zero. De Rensis et al. (2017) suggested that the impact of HS on the traits could be determined by the presence and intensity of factors responsible for seasonal infertility. For this study, we used climate data recorded by public weather stations. This may have affected the estimation of the magnitude of GxE since the conditions measured by the weather stations may differ from the indoor farm conditions. The use of public weather data is common in studies of this kind, but further studies should consider using indoor-recorded conditions.

### Estimations of Reaction Norms and Identification of HS Tolerant Genetic Material

In a breeding scheme where HT is considered, an alternative could be selecting the best performing individuals under HS, provided that they are not under-performing in standard (comfortable) environmental conditions. The identification of such genetic resources (breeding animals) can be easily performed graphically as shown in Figure 2 (the reaction norms for the most and least heat tolerant sires are reported for the three cases studies on TNB, NBA, and ABW, in sections A, B and C, respectively). For TNB and ABW the most HT sires (blue lines) appear also to be the best performing in the low-ENV conditions. For NBA, the opposite relationship was observed, in which the better performing sires are also the least HT, with a stronger decline in performance due to HS than the population average.

### Genome-Wide Association Study and Functional Annotation

In this study we used single-step approach to obtain marker effect estimates. This choice was dictated by the fact that not all phenotyped individuals were genotyped and *vice versa*. A comparison of the number of genotyped individuals (Table 1) and the number of phenotyped individuals (Table 2) shows the data structure. The proportion of sires genotyped ranged from 28 to 70%, while the number of sows genotyped ranged from 18 to 42%. The use of single-step GBLUP allowed the efficient inclusion of all available information while a reduced sample size and loss of sire genomic information would have occurred if only individuals with both genotype and phenotypes were used.

A large number of significant genomic windows distributed across all swine autosomal chromosomes were identified to be associated with HT based on reproductive traits. In general, each of these multiple genomic regions explained a small proportion (<0.5%) of the total additive genetic variance, indicating that HT is a largely polygenic trait. However, the functional analyses performed confirm that the indicator traits analyzed are involved in important biological mechanisms of HS response. For instance, the genomic regions identified were previously reported to be associated with traits such as body temperature, hemoglobin content, coping behavior, cortisol levels and heart size. Various pathways associated with fatty acid biosynthesis were also identified.

As expected, the genomic regions associated with the intercept (average performance at the comfort environmental gradient) were previously linked with reproductive traits such as litter size, number of mummified piglets, corpus luteum number, number of non-viable fetuses, maternal infanticide, testicular width and length, and semen pH. The fact that the present study identified genomic regions previously reported to be associated with related variables validates the indicator traits used in this study.

Various candidate genes identified in this study to be associated with HT (slope of the reaction norms) were previously reported in the literature as well. For instance, *ALDH1A3* and *LRRK1* genes have been highly associated with multiple reproductive traits (e.g., numbers of litter per sow per year, piglets weaned per litter, NBA, weaning to conception interval) in LW pigs (Suwannasing et al., 2018). The aldehyde dehydrogenase (*ALDH*) genes family plays an important role in embryo formation and development, cell proliferation and differentiation (Duan et al., 2016). The genes *ARHGAP21* and *LSMEM2* have been reported to be associated with immune responses in LR, LW and Songliao Black Pig piglets based on hematological traits after being immunized with classical swine fever vaccine (Wang et al., 2013). Important genomic regions within the *ESR2* gene (Estrogen receptor) were also identified. *ESR2* has been previously reported to be associated with reproductive traits (e.g., litter size, maternal infanticide, TNB, NBA, semen volume, semen concentration) in various pig breeds (Chen et al., 2011; Gunawan et al., 2012; Laliotis et al., 2017). Interestingly, the gene *LIMK2* has been previously associated with melanoma susceptibility in Duroc pigs (Bourneuf et al., 2018). Genes associated with body characteristics have also been reported. This includes *PLOD1* and *NRXN3*, which have been associated with traits such as diaphragm weight, carcass length, spinal curvature and belly weight (Lindholm-Perry et al., 2010; Li et al., 2011; Sato et al., 2016) and various other candidate genes (e.g., *AASS, AP3D1, CPT1A, DOCK1, FASN, LRRK2, PDE1C, PPA2, PSMD1, SCUBE1, SLC27A6, SNAI2, TFAP2B, TMPRSS4, UBAP2, WDR47*) previously linked to carcass fatness, fatty acid biosynthesis, average daily gain, and body weight in various pig breeds, including LW and LR (Edwards et al., 2008; Li et al., 2011; Fontanesi et al., 2012, 2014; Do et al., 2013; Fowler et al., 2013; Nonneman et al., 2013; Choi et al., 2016; Sato et al., 2016; Reyer et al., 2017; Lee et al., 2019; Zappaterra et al., 2019). Some candidate genes, such as *CADPS2*, were also associated with feeding behavior, e.g., number of daily visits to the feeder in Duroc pigs (Do et al., 2013). The pathways, biological processes and cellular components identified in alternative functional analyses were similar, which indicates a large agreement across the results presented. Future studies should focus on validating the current findings on crossbred animals and in other independent populations (e.g., breeds). The use of better measures of climatic conditions (e.g., indoor barn temperature, humidity) and closer-to-biology phenotypes (with higher heritability) will result in more accurate breeding values for HT as well as facilitate the identification of genomic regions and candidate genes associated with HS response. Investigating the genetic relationship between HT indicators and other economically important traits is of great importance when designing breeding programs and will be the objective of future studies.

## Conclusions

In the present study, we used four independent populations to explore the genetic and genomic background of heat tolerance in maternal-line pigs, based on three reproductive traits and multiple environmental covariates. Our results indicate that heat tolerance based on reproductive traits is heritable and therefore genetic progress for HT can be achieve through genetic or genomic selection. Higher heritability estimates were observed in comfortable environments, indicating that selection for improved heat tolerance based on reproductive traits is feasible, although genetic progress will be slow. Therefore, considering that there is substantial additive genetic variance for heat tolerance, there is a need to identify novel indicators that better capture the biological mechanisms of HS response. Various genomic regions distributed across all autosomal chromosomes and explaining a small proportion of the total additive genetic variance were identified, indicating that heat tolerance is a highly polygenic trait. Results validated across the populations seem to point out to a genomic region located on SSC9 showing a sizable impact on heat tolerance measured both on TNB and NBA. This region will require future validation and refinement in future studies. In summary, we have shown that selection for improved heat tolerance in swine based on reproductive performance and public weather station data is possible. Selection for heat tolerance is expected to alter important biological mechanisms underlying several traits. Future research should focus on elucidating these mechanisms on a wide array of economically important traits before heat tolerance can be included among the breeding goals.

## Data Availability Statement

Phenotypic and genomic data used in this study are property of the industry partners that contributed to the study. The data were given as in kind for conducting the study being published. The industry partners retain exclusive access to the data since it includes information about animals' performance and genetic background. The industry partners require that all data is deleted once the study is conducted. Requests to access these datasets may be granted under a negotiated research agreement and should be directed to Justin Fix, justin.fix@pigsrus.net; Kent Gray, kgray@smithfield.com.

## Ethics Statement

Ethical review and approval was not required for the animal study because The data used in this study came from pre-collected commercial datasets. Written informed consent for participation was not obtained from the owners because Representatives of owner companies contributed to the study and are listed as co-authors.

## Author Contributions

FT and CM conceived and designed this study. FT carried out the analyses. FT, CM, and LB interpreted and discussed the results and wrote the manuscript. JH, YH, KG, JF, and CS supervised the data collection and provided inputs for the analyses of the data. All the authors reviewed and approved the final manuscript.

## Conflict of Interest

The study used at that were provided as in kind by companies Smithfield Premium Genetics and The Maschhoffs LLC. JH, YH, and KG were employed by the company Smithfield Premium Genetics. CS and JF were employed by The Maschhoffs LLC. At the time of submission. The results are commercially of interest to the above mentioned companies but this interest did not influence the results presented in this manuscript in any matter. The remaining authors declare that the research was conducted in the absence of any commercial or financial relationships that could be construed as a potential conflict of interest.

## Supplementary Material

Figure S1a Additive genetic variance estimates (posterior means with 95% empirical confidence intervals) for total number of piglets born (TNB) in the Smithfield Premium Genetics Landrace population (SPG_LR) over the range of Average Relative Humidity 14 to 7 days before conception.

Figure S1b Additive genetic variance estimates (posterior means with 95% empirical confidence intervals) for number of piglets born alive (NBA) in the Landrace population of the Maschhoffs breeding company (TML_LR) over the range of average THI 86 to 93 days into pregnancy.

Figure S1c Additive genetic variance estimates (posterior means with 95% empirical confidence intervals) for average birth weight (ABW) in the Yorkshire population of the Maschhoffs breeding company (TML_YS) over the range of average relative humidity 107 to 114 days into pregnancy.

Figure S2a Residual variance estimates (posterior means with 95% empirical confidence intervals) for total number of piglets born (TNB) in the Smithfield Premium Genetics Landrace population (SPG_LR) over the range of Average Relative Humidity 14 to 7 days before conception.

Figure S2b Residual variance estimates (posterior means with 95% empirical confidence intervals) for number of piglets born alive (NBA) in the Landrace population of the Maschhoffs breeding company (TML_LR) over the range of average THI 86 to 93 days into pregnancy.

Figure S2c Residual variance estimates (posterior means with 95% empirical confidence intervals) for average birth weight (ABW) in the Yorkshire population of the Maschhoffs breeding company (TML_YS) over the range of average relative humidity 107 to 114 days into pregnancy.

Figure S3 Schematic representation of the metabolic functions (PANTHER Classification System) and the proportion of candidate genes identified in the genome-wide association analyses for the intercept of the genomic reaction norms based on reproductive traits in four maternal-line pig populations.

Figure S4 Schematic representation of the biological processes (PANTHER Classification System) and the proportion of candidate genes identified in the genome-wide association analyses for the intercept of the genomic reaction norms based on reproductive traits in four maternal-line pig populations.

Figure S5 Schematic representation of the cellular components (PANTHER Classification System) and the proportion of candidate genes identified in the genome-wide association analyses for the intercept of the genomic reaction norms based on reproductive traits in four maternal-line pig populations.

Figure S6 Schematic representation of the molecular functions (PANTHER Classification System) and the proportion of candidate genes identified in the genome-wide association analyses for the slope of the genomic reaction norms based on reproductive traits in four maternal-line pig populations.

Figure S7 Schematic representation of the biological processes (PANTHER Classification System) and the proportion of candidate genes identified in the genome-wide association analyses for the slope of the genomic reaction norms based on reproductive traits in four maternal-line pig populations.

Figure S8 Schematic representation of the cellular components (PANTHER Classification System) and the proportion of candidate genes identified in the genome-wide association analyses for the slope of the genomic reaction norms based on reproductive traits in four maternal-line pig populations.

Table S1 Summary of the environmental covariates tested in this study. Each row in the table reports a summary of the 19 weekly values for the population.

Table S2a Significant genomic windows for the intercept term of the reaction norm on total number of piglets born (TNB) as regressed on the Average Relative Humidity 14 to 7 days before conception in the Smithfield Premium Genetics (SPG) Landrace population (SPG_LR).

Table S2b Significant genomic windows for the slope term of the reaction norm on total number of piglets born (TNB) as regressed on the Average Relative Humidity 14 to 7 days before conception in the Smithfield Premium Genetics (SPG) Landrace population (SPG_LR).

Table S3a Significant windows for intercept term on number of piglets born alive (NBA) as regressed on average THI 86 to 93 days into pregnancy in the Landrace population of the Maschhoffs breeding company (TML_LR).

Table S3b Significant windows for slope term on number of piglets born alive (NBA) as regressed on average THI 86 to 93 days into pregnancy in the Landrace population of the Maschhoffs breeding company (TML_LR).

Table S4a Significant windows for the intercept term on average birth weight (ABW) as regressed on average relative humidity 107 to 114 days into pregnancy in the Yorshire population of the Maschhoffs breeding company (TML_YS).
