# Supplementary material for: Genomics of Heat Tolerance in Reproductive Performance Investigated in Four Independent Maternal Lines of Pigs
Source: Front Genet. 2020 Jun 30;11:629. doi: 10.3389/fgene.2020.00629 (PMC7338773; doi:10.3389/fgene.2020.00629)
Supplement: Figure S1a — Additive genetic variance estimates (posterior means with 95% empirical confidence intervals) for total number of piglets born (TNB) in the Smithfield Premium Genetics Landrace population (SPG_LR) over the range of Average Relative Humidity 14 to 7 days before conception. [file Data_Sheet_1.zip › Supplementary files/SupplementaryTableS4a.pdf]

Table S4a: Significant windows for the intercept term on average birth weight (ABW) as regressed on average relative humidity 107 to 114 days into pregnancy in the Yorshire population of the Maschhoffs breeding company (TML_YS).

Location	Var(%) and N SNPs in brackets	Positional genes	QTL traits
SSC9: 75,823,479-81,214,756	0.42(55)	*ENSSSCG00000015336 (SLC25A13), ENSSSCG00000051379, ENSSSCG00000047863, ENSSSCG00000015337, ENSSSCG00000051115, ENSSSCG00000047552, ENSSSCG00000048105, ENSSSCG00000049944, ENSSSCG00000051124, ENSSSCG00000033314 (DLX6), ENSSSCG00000038838, ENSSSCG00000023850, ENSSSCG00000023592 (TAC1), ENSSSCG00000045298, ENSSSCG00000015340, ENSSSCG00000050392, ENSSSCG00000038187, ENSSSCG00000049770, ENSSSCG00000015342 (COL28A1), ENSSSCG00000015343 (MIOS), ENSSSCG00000023886 (RPA3), ENSSSCG00000036169 (UMAD1), ENSSSCG00000015345 (GLCCI1), ENSSSCG00000015346 (ICA1), ENSSSCG00000046846, ENSSSCG00000037207 (NXPH1), ENSSSCG00000045901, ENSSSCG00000046900, ENSSSCG00000042925, ENSSSCG00000051507, ENSSSCG00000051543, ENSSSCG00000024313, ENSSSCG00000027591, ENSSSCG00000021544, ENSSSCG00000026722 (PHF14), ENSSSCG00000047616, ENSSSCG00000015350 (THSD7A)*	45 min-24 hours pH decline, dressing percentage, oiliness, PRRS viral load, piglet mortality, average daily gain, immunocrit, number of non-viable fetuses, body length, hip structure, front leg conformation, hind leg conformation, front feet conformation, gait score (overall), rib shape, semen pH, anal atresia, spleen weight
SSC9: 55,824,884-58,228,279	0.38(28)	*ENSSSCG00000021573, ENSSSCG00000015238 (ARHGAP32), ENSSSCG00000043746, ENSSSCG00000036157 (BARX2), ENSSSCG00000041877, ENSSSCG00000050593, ENSSSCG00000041798, ENSSSCG00000045608, ENSSSCG00000050270, ENSSSCG00000040319, ENSSSCG00000015241 (TMEM45B), ENSSSCG00000015242 (NFRKB), ENSSSCG00000015243 (PRDM10), ENSSSCG00000015244 (APLP2), ENSSSCG00000015246 (ST14), ENSSSCG00000015247 (ZBTB44), ENSSSCG00000046782, ENSSSCG00000015249 (ADAMTS8), ENSSSCG00000015250 (ADAMTS15), ENSSSCG00000042049, ENSSSCG00000015252 (SNX19), ENSSSCG00000048987, ENSSSCG00000020352, ENSSSCG00000048504, ENSSSCG00000045830, ENSSSCG00000015253 (NTM)*	Hind leg conformation, hind feet conformation, fat percentage in carcass, lean meat percentage, meat to fat ratio, carcass length, backfat at rump

SSC14: 114,610,574-117,846,180	0.37(51)	*ENSSSCG00000010604 (SH3PXD2A), ENSSSCG00000034344, ENSSSCG00000010605 (STN1), ENSSSCG00000010606 (SLK), ENSSSCG00000010607 (COL17A1), ENSSSCG00000049538, ENSSSCG00000010608 (SFR1), ENSSSCG00000010609 (CFAP43), ENSSSCG00000010610, ENSSSCG00000022351, ENSSSCG00000030271 (GSTO2), ENSSSCG00000010613 (ITPRIP), ENSSSCG00000010614 (CFAP58), ENSSSCG00000010617 (SORCS3), ENSSSCG00000033470, ENSSSCG00000018956, ENSSSCG00000020604, ENSSSCG00000047988, ENSSSCG00000030026 (SORCS1)*	Dressing percentage, Palmitoleic acid content, Stearic acid content, Monounsaturated fatty acid content, loin pH 24 hours *post-mortem*, hematocrit, red blood cell count, litter size, gestation length, Palmitic acid content, Oleic acid content, Saturated fatty acid content
SSC18: 31,560,363-32,946,579	0.34(19)	*ENSSSCG00000016639 (FOXP2), ENSSSCG00000045847, ENSSSCG00000016640 (PPP1R3A), ENSSSCG00000051047, ENSSSCG00000048496, ENSSSCG00000042801, ENSSSCG00000048245, ENSSSCG00000016642 (GPR85), ENSSSCG00000021466 (BMT2), ENSSSCG00000016643 (TMEM168)*	Chronic pleuritis, *Salmonella* count in spleen, hematocrit, cooking loss, loin pH 24 hours *post-mortem*, birth weight variability, average daily gain, hip structure
SSC9: 58,398,881-60,527,068	0.34(25)	*ENSSSCG00000015253 (NTM), ENSSSCG00000018246, ENSSSCG00000049894, ENSSSCG00000037534 (OPCML), ENSSSCG00000048884, ENSSSCG00000045081, ENSSSCG00000025612, ENSSSCG00000015255 (IGSF9B)*	Body weight, chest circumference
SSC1: 4,058,074-4,894,265	0.34(20)	*ENSSSCG00000050396, ENSSSCG00000046688, ENSSSCG00000042992, ENSSSCG00000004029 (QKI)*	.
SSC6: 82,457,398-84,047,659	0.32(35)	*ENSSSCG00000038994 (CLIC4), ENSSSCG00000041070, ENSSSCG00000032686 (RUNX3), ENSSSCG00000037664, ENSSSCG00000032169, ENSSSCG00000037481 (SYF2), ENSSSCG00000034449 (RSRP1), ENSSSCG00000024267 (TMEM50A), ENSSSCG00000026791, ENSSSCG00000030682, ENSSSCG00000003549 (LDLRAP1), ENSSSCG00000050714, ENSSSCG00000023235 (MAN1C1),*	Meat color a*, thoracic vertebra number, melanoma susceptibility, birth weight

		ENSSSCG00000003554 (SELENON), ENSSSCG00000003553 (MTFR1L), ENSSSCG00000003552 (AUNIP), ENSSSCG00000003551 (PAQR7), ENSSSCG00000026257 (STMN1), ENSSSCG00000043402, ENSSSCG00000038619 (PAFAH2), ENSSSCG00000029558 (EXTL1), ENSSSCG00000026386, ENSSSCG00000027684, ENSSSCG00000041335, ENSSSCG00000030660 (PDIK1L), ENSSSCG00000031074 (FAM110D), ENSSSCG00000041671 (C1orf232), ENSSSCG00000045776, ENSSSCG00000028387 (CNKSR1), ENSSSCG00000026526 (CATSPER4), ENSSSCG00000045528, ENSSSCG00000026731 (CEP85), ENSSSCG00000033546 (UBXN11), ENSSSCG00000034937 (SH3BGRL3), ENSSSCG00000033539 (CRYBG2), ENSSSCG00000040579 (ZNF683), ENSSSCG00000003557, ENSSSCG00000033516, ENSSSCG00000003555, ENSSSCG00000049882, ENSSSCG00000048981, ENSSSCG00000003558 (RPS6KA1), ENSSSCG00000003559 (ARID1A)	
SSC9: 28,237,601-28,671,488	0.31(16)	*ENSSSCG00000034879 (MAML2), ENSSSCG00000014972 (CCDC82), ENSSSCG00000034924 (JRKL)*	Adrenal gland weight, muscle protein percentage, head weight
SSC9: 934,647-2,952,003	0.31(27)	*ENSSSCG00000030206 (STK33), ENSSSCG00000023057 (LMO1), ENSSSCG00000014580 (RIC3), ENSSSCG00000014581 (TUB), ENSSSCG00000039647 (EIF3F), ENSSSCG00000042620, ENSSSCG00000014587, ENSSSCG00000031202, ENSSSCG00000036687, ENSSSCG00000050659, ENSSSCG00000032130, ENSSSCG00000034067, ENSSSCG00000044445, ENSSSCG00000036240, ENSSSCG00000035773, ENSSSCG00000047231, ENSSSCG00000031845, ENSSSCG00000050809, ENSSSCG00000036606, ENSSSCG00000036929, ENSSSCG00000050053, ENSSSCG00000039848, ENSSSCG00000049272, ENSSSCG00000046240, ENSSSCG00000044470, ENSSSCG00000021619 (OVCH2), ENSSSCG00000049432, ENSSSCG00000048244, ENSSSCG00000047740, ENSSSCG00000014598 (PPFIBP2), ENSSSCG00000038540, ENSSSCG00000014599 (SYT9), ENSSSCG00000045371, ENSSSCG00000042696, ENSSSCG00000047897, ENSSSCG00000014600, ENSSSCG00000038122, ENSSSCG00000031270 (OR2D2), ENSSSCG00000014602 (OR10A4), ENSSSCG00000039077, ENSSSCG00000014617 (OR6A2), ENSSSCG00000049448,*	Hematocrit, red blood cell count, red cell distribution width, coping behavior, litter weight, plateletcrit, number of mummified piglets

		ENSSSCG00000044240, ENSSSCG00000014608, ENSSSCG00000014605, ENSSSCG00000045220, ENSSSCG00000043559, ENSSSCG00000044796, ENSSSCG00000033089, ENSSSCG00000031610, ENSSSCG00000020258	
SSC2: 145,257,971-145,963,826	0.30(19)	*ENSSSCG00000041721, ENSSSCG00000048807, ENSSSCG00000043421, ENSSSCG00000039309 (YIPF5), ENSSSCG00000032826 (KCTD16), ENSSSCG00000028579*	.
SSC2: 138,960,347-139,629,298	0.29(18)	*ENSSSCG00000032423 (SPOCK1)*	Intramuscular fat content
SSC10: 50,346,134-51,261,717	0.29(19)	*ENSSSCG00000028135 (PRTFDC1), ENSSSCG00000037690, ENSSSCG00000011074 (ARHGAP21), ENSSSCG00000011075 (KIAA1217), ENSSSCG00000050240, ENSSSCG00000050564*	Anal atresia, scrotal/inguinal hernia, teat number, 3-24 hours pH decline, age at puberty
SSC2: 106,921,556-107,759,802	0.28(21)	*ENSSSCG00000045323, ENSSSCG00000046316, ENSSSCG00000033364*	Shear force, tenderness score, average instron (star probe) force, pH 24 hour post mortem (ham), juiciness score, chew score, drip loss, backfat at first rib, flavor score, cooking loss, carcass fatness, marbling, shear force at first peak, meat color b*, meat color L*, muscle fat content, muscle protein percentage, muscle moisture percentage, spinal curvature
SSC15: 137,470,306-138,275,968	0.25(21)	*ENSSSCG00000016332 (RBM44), ENSSSCG00000016331, ENSSSCG00000027723, ENSSSCG00000041251, ENSSSCG00000051289, ENSSSCG00000016334 (SCLY), ENSSSCG00000016335 (ESPNL), ENSSSCG00000026600 (KLHL30), ENSSSCG00000016337 (ERFE),*	Maternal infanticide, number of mummified piglets

		ENSSSCG00000016339 (ILKAP), ENSSSCG00000048787, ENSSSCG00000016342 (HES6), ENSSSCG00000016338 (PER2), ENSSSCG00000051024, ENSSSCG00000025228 (TRAF3IP1), ENSSSCG00000016340 (ASB1), ENSSSCG00000042914, ENSSSCG00000016343	
SSC7: 102,377,016-102,949,951	0.24(13)	*ENSSSCG00000028942 (NRXN3)*	Lymphocyte number, white blood cell number, carcass length, cholesterol level, thoracic vertebra number, obesity index, teat number
SSC9: 3,053,106-3,924,348	0.24(17)	*ENSSSCG00000014600, ENSSSCG00000014618, ENSSSCG00000024693, ENSSSCG00000029189 (DCHS1), ENSSSCG00000023080 (TPP1), ENSSSCG00000029630 (TAF10), ENSSSCG00000023272 (ILK), ENSSSCG00000023880 (RRP8), ENSSSCG00000039799, ENSSSCG00000045625, ENSSSCG00000037299, ENSSSCG00000014625 (TRIM3), ENSSSCG00000014626 (HPX), ENSSSCG00000014628 (APBB1), ENSSSCG00000014627 (SMPD1), ENSSSCG00000014633 (CAVIN3), ENSSSCG00000033806, ENSSSCG00000014630 (CNGA4), ENSSSCG00000014632 (FAM160A2), ENSSSCG00000014634 (C11orf42), ENSSSCG00000046332, ENSSSCG00000035041 (OR52W1), ENSSSCG00000045056, ENSSSCG00000050079, ENSSSCG00000044868, ENSSSCG00000040404 (OR56B4), ENSSSCG00000014639 (OR56A1), ENSSSCG00000049205, ENSSSCG00000040084, ENSSSCG00000047710, ENSSSCG00000048497 (OR52E4), ENSSSCG00000041574, ENSSSCG00000043308, ENSSSCG00000041779, ENSSSCG00000051537, ENSSSCG00000049377, ENSSSCG00000049604, ENSSSCG00000049817, ENSSSCG00000031842, ENSSSCG00000038324, ENSSSCG00000050910, ENSSSCG00000046786, ENSSSCG00000048561, ENSSSCG00000043101, ENSSSCG00000036692, ENSSSCG00000045340, ENSSSCG00000049791, ENSSSCG00000041301, ENSSSCG00000047213, ENSSSCG00000042301, ENSSSCG00000040477, ENSSSCG00000031864, ENSSSCG00000014643, ENSSSCG00000050291, ENSSSCG00000043499 (OR52E5), ENSSSCG00000044027, ENSSSCG00000048134*	Hemoglobin, body height

SSC13: 32,238,776-32,792,383	0.23(12)	*ENSSSCG00000011387 (RNF123), ENSSSCG00000011389 (AMIGO3), ENSSSCG00000011390 (IP6K1), ENSSSCG00000011391 (CDHR4), ENSSSCG00000011393 (UBA7), ENSSSCG00000049797, ENSSSCG00000026977 (TRAIP), ENSSSCG00000025416 (CAMKV), ENSSSCG00000027992 (MST1R), ENSSSCG00000025791, ENSSSCG00000011394 (RBM6), ENSSSCG00000037003, ENSSSCG00000011395 (RBM5), ENSSSCG00000011398 (SEMA3F), ENSSSCG00000042718 (GNAT1), ENSSSCG00000011397 (SLC38A3), ENSSSCG00000011399 (GNAI2), ENSSSCG00000011400 (SEMA3B), ENSSSCG00000011401 (LSMEM2), ENSSSCG00000011402 (IFRD2), ENSSSCG00000041673, ENSSSCG00000024363 (HYAL3), ENSSSCG00000011404*	.
SSC12: 9,655,798-10,354,335	0.23(15)	*ENSSSCG00000048915, ENSSSCG00000043713, ENSSSCG00000042724, ENSSSCG00000042576, ENSSSCG00000039947 (KCNJ2)*	Birth weight, immunocrit, meat color b*, small intestine length, hind leg conformation
SSC2: 21,028,892-22,252,988	0.23(19)	*ENSSSCG00000013286 (LRRC4C), ENSSSCG00000049735, ENSSSCG00000047030, ENSSSCG00000043947*	Conductivity 45 minutes post-mortem, coping behavior, sperm per ejaculate
SSC1: 15,731,768-16,179,177	0.23(15)	*ENSSSCG00000040966, ENSSSCG00000004093 (IYD), ENSSSCG00000004094 (PPP1R14C), ENSSSCG00000035574, ENSSSCG00000032525, ENSSSCG00000033578, ENSSSCG00000004096, ENSSSCG00000041378, ENSSSCG00000026042, ENSSSCG00000004101 (LRP11)*	Corpus luteum number, obesity index
SSC1: 138,927,010-140,260,943	0.23(14)	*ENSSSCG00000038452 (ADAMTS17), ENSSSCG00000004819 (CERS3), ENSSSCG00000004820 (LINS1), ENSSSCG00000004821 (ASB7), ENSSSCG00000047618, ENSSSCG00000038358, ENSSSCG00000004822 (ALDH1A3), ENSSSCG00000004823 (LRRK1), ENSSSCG00000019106, ENSSSCG00000048312, ENSSSCG00000045683, ENSSSCG00000004825, ENSSSCG00000044912, ENSSSCG00000004826, ENSSSCG00000035584 (GABRG3), ENSSSCG00000019280, ENSSSCG00000042260*	Number of mummified piglets, Linoleic acid content, Monounsaturated fatty acid to saturated fatty acid ratio, drip loss
SSC9: 117,742,788-118,481,227	0.22(15)	*ENSSSCG00000015508 (TNR), ENSSSCG00000047811, ENSSSCG00000015509 (COP1), ENSSSCG00000034100 (SCARNA3), ENSSSCG00000046238, ENSSSCG00000044431, ENSSSCG00000049556, ENSSSCG00000015512 (PAPPA2)*	Daily feed intake

SSC14: 47,696,012-48,356,407	0.22(18)	*ENSSSCG00000010015 (OSBP2), ENSSSCG00000010016 (MORC2), ENSSSCG00000040413, ENSSSCG00000010017, ENSSSCG00000029781, ENSSSCG00000030345 (INPP5J), ENSSSCG00000010023 (PLA2G3), ENSSSCG00000010025 (LIMK2), ENSSSCG00000010026 (PIK3IP1), ENSSSCG00000010027 (PATZ1), ENSSSCG00000010029 (DRG1), ENSSSCG00000010030 (EIF4ENIF1), ENSSSCG00000035290, ENSSSCG00000010031, ENSSSCG00000010032 (PISD), ENSSSCG00000010033 (PRR14L)*	.
SSC1: 52,520,680-53,604,351	0.22(12)	*ENSSSCG00000004281 (KCNQ5), ENSSSCG00000046325, ENSSSCG00000049177, ENSSSCG00000050380, ENSSSCG00000004283, ENSSSCG00000004284, ENSSSCG00000004285, ENSSSCG00000004287, ENSSSCG00000022994 (CYB5R4), ENSSSCG00000026943 (MRAP2), ENSSSCG00000044858, ENSSSCG00000004289 (CEP162)*	Age at puberty
SSC2: 108,425,985-109,218,177	0.22(13)	*ENSSSCG00000014184 (PAM), ENSSSCG00000025287 (GIN1), ENSSSCG00000024999 (PPIP5K2), ENSSSCG00000049232, ENSSSCG00000043322, ENSSSCG00000045132, ENSSSCG00000042485, ENSSSCG00000038779 (C5orf30), ENSSSCG00000048901, ENSSSCG00000049923, ENSSSCG00000014186, ENSSSCG00000014187 (NUDT12), ENSSSCG00000047052, ENSSSCG00000043606*	Number of mummified piglets
SSC14: 46,614,469-47,196,197	0.21(16)	*ENSSSCG00000009988 (THOC5), ENSSSCG00000051359 (NIPSNAP1), ENSSSCG00000028707 (NF2), ENSSSCG00000025869 (CABP7), ENSSSCG00000024279 (ZMAT5), ENSSSCG00000024550, ENSSSCG00000025186 (ASCC2), ENSSSCG00000009994 (MTMR3), ENSSSCG00000043717, ENSSSCG00000046492, ENSSSCG00000009995 (HORMAD2), ENSSSCG00000045744*	Intramuscular fat content
SSC18: 35,927,576-36,434,158	0.21(11)	*ENSSSCG00000044944, ENSSSCG00000043837, ENSSSCG00000048016, ENSSSCG00000049426*	.
SSC15: 133,730,356-134,172,205	0.20(12)	*ENSSSCG00000016301 (SAG), ENSSSCG00000009432 (DGKD), ENSSSCG00000042816, ENSSSCG00000016306 (USP40), ENSSSCG00000036274, ENSSSCG00000049095, ENSSSCG00000028217, ENSSSCG00000016305 (MROH2A), ENSSSCG00000016313 (HJURP)*	Loin pH 24 hours *post-mortem*, drip loss, cooking loss, ham pH 24 hours *post-mortem*, meat color a*

SSC4: 43,833,235-44,642,552	0.20(14)	*ENSSSCG00000035526, ENSSSCG00000046234, ENSSSCG00000050430, ENSSSCG00000006115 (RUNX1T1)*	Drip loss, teat number
SSC13: 95,329,431-97,080,352	0.20(10)	*ENSSSCG00000026718 (PLCH1), ENSSSCG00000048315, ENSSSCG00000025316 (SLC33A1), ENSSSCG00000028855 (GMPS), ENSSSCG00000019096, ENSSSCG00000011724, ENSSSCG00000025810 (KCNAB1), ENSSSCG00000026410, ENSSSCG00000047110, ENSSSCG00000030567 (SSR3), ENSSSCG00000046794, ENSSSCG00000048772, ENSSSCG00000027646 (TIPARP), ENSSSCG00000045498, ENSSSCG00000026579, ENSSSCG00000047487, ENSSSCG00000041932, ENSSSCG00000048257, ENSSSCG00000029352 (CCNL1), ENSSSCG00000011726 (VEPH1)*	Total number of piglets born alive, spleen weight
SSC4: 12,527,995-13,008,743	0.20(11)	*ENSSSCG00000048613, ENSSSCG00000042693, ENSSSCG00000050281, ENSSSCG00000045505, ENSSSCG00000049874*	Intramuscular fat content, teat number, fat-cuts percentage
SSC14: 46,043,439-46,408,884	0.20(11)	*ENSSSCG00000009971 (ZNRF3), ENSSSCG00000025404 (C22orf31), ENSSSCG00000009972 (KREMEN1), ENSSSCG00000009975 (EMID1), ENSSSCG00000009973 (RHBDD3), ENSSSCG00000009974 (EWSR1)*	Body weight (birth)
SSC6: 75,839,337-76,292,118	0.19(12)	*ENSSSCG00000003481 (PADI1), ENSSSCG00000003482 (PADI3), ENSSSCG00000003483 (PADI4), ENSSSCG00000003484 (PADI6), ENSSSCG00000003485 (RCC2), ENSSSCG00000003486 (ARHGEF10L), ENSSSCG00000046919*	Oleic acid content, Monounsaturated fatty acid content, Monounsaturated fatty acid to saturated fatty acid ratio, Palmitoleic acid content, Linoleic acid content
SSC15: 16,171,215-16,718,985	0.19(12)	*ENSSSCG00000015690 (UBXN4), ENSSSCG00000015692 (R3HDM1), ENSSSCG00000019503, ENSSSCG00000015694 (ZRANB3), ENSSSCG00000018804, ENSSSCG00000039294, ENSSSCG00000045925*	Coping behavior, platelet count
SSC3: 128,201,541-128,497,956	0.19(10)	*ENSSSCG00000046300, ENSSSCG00000043310*	.

SSC6: 133,060,727-133,334,031	0.19(12)	.	.
SSC12: 35,139,128-36,028,648	0.19(11)	*ENSSSCG00000038932 (PPM1E), ENSSSCG00000023727 (TRIM37), ENSSSCG00000034217 (SKA2), ENSSSCG00000018920 (MIR454), ENSSSCG00000028212 (MIR301A), ENSSSCG00000024145 (PRR11), ENSSSCG00000024411, ENSSSCG00000030262, ENSSSCG00000035400 (YPEL2), ENSSSCG00000043506, ENSSSCG00000017665 (DHX40), ENSSSCG00000022659 (CLTC), ENSSSCG00000017667 (PTRH2), ENSSSCG00000017668 (VMP1)*	Backfat between 3rd and 4th last ribs
SSC9: 47,166,781-47,399,155	0.18(10)	*ENSSSCG00000015126 (TRIM29), ENSSSCG00000044597, ENSSSCG00000036307, ENSSSCG00000028237 (POU2F3)*	Dressing percentage, backfat between 3rd and 4th last ribs
SSC14: 59,610,682-60,318,934	0.18(10)	*ENSSSCG00000010182 (CAPN9), ENSSSCG00000010184 (AGT), ENSSSCG00000010183 (COG2), ENSSSCG00000048229, ENSSSCG00000021027 (PGBD5), ENSSSCG00000010185 (GALNT2), ENSSSCG00000046435, ENSSSCG00000010187 (TAF5L), ENSSSCG00000010186 (URB2), ENSSSCG00000010188 (ABCB10)*	.
SSC6: 81,775,011-82,207,445	0.18(10)	*ENSSSCG00000024834, ENSSSCG00000036534 (MYOM3), ENSSSCG00000038848 (IL22RA1), ENSSSCG00000032860 (IFNLR1), ENSSSCG00000003539 (GRHL3), ENSSSCG00000003541 (STPG1), ENSSSCG00000003543 (NIPAL3), ENSSSCG00000024818 (RCAN3), ENSSSCG00000003547 (NCMAP)*	Litter weight
SSC15: 18,365,207-18,972,285	0.18(10)	*ENSSSCG00000041827, ENSSSCG00000043458, ENSSSCG00000048606, ENSSSCG00000046592, ENSSSCG00000042339, ENSSSCG00000043451, ENSSSCG00000046308, ENSSSCG00000043328, ENSSSCG00000023479, ENSSSCG00000051377*	Front feet conformation
SSC10: 2,344,592-2,801,475	0.18(11)	*ENSSSCG00000050890, ENSSSCG00000043005, ENSSSCG00000042899*	LDL cholesterol
SSC9: 53,428,993-53,975,532	0.18(10)	*ENSSSCG00000015231 (DCPS), ENSSSCG00000015232 (ST3GAL4), ENSSSCG00000015234 (KIRREL3)*	.

SSC9: 103,696,209-105,612,360	0.18(10)	*ENSSSCG00000015426 (RELN), ENSSSCG00000015427 (ORC5), ENSSSCG00000035554 (LHFPL3), ENSSSCG00000029082 (KMT2E), ENSSSCG00000026110 (SRPK2), ENSSSCG00000015428 (PUS7), ENSSSCG00000046436, ENSSSCG00000015430 (RINT1), ENSSSCG00000015432 (ATXN7L1)*	.
SSC5: 80,795,197-81,281,908	0.18(10)	*ENSSSCG00000000854, ENSSSCG00000034578, ENSSSCG00000043357, ENSSSCG00000000856 (PAH)*	Coping behavior
