# Supplementary material for: Genomics of Heat Tolerance in Reproductive Performance Investigated in Four Independent Maternal Lines of Pigs
Source: Front Genet. 2020 Jun 30;11:629. doi: 10.3389/fgene.2020.00629 (PMC7338773; doi:10.3389/fgene.2020.00629)
Supplement: Figure S1a — Additive genetic variance estimates (posterior means with 95% empirical confidence intervals) for total number of piglets born (TNB) in the Smithfield Premium Genetics Landrace population (SPG_LR) over the range of Average Relative Humidity 14 to 7 days before conception. [file Data_Sheet_1.zip › Supplementary files/Suplementary_Figures_S6_S7_s8.pdf]

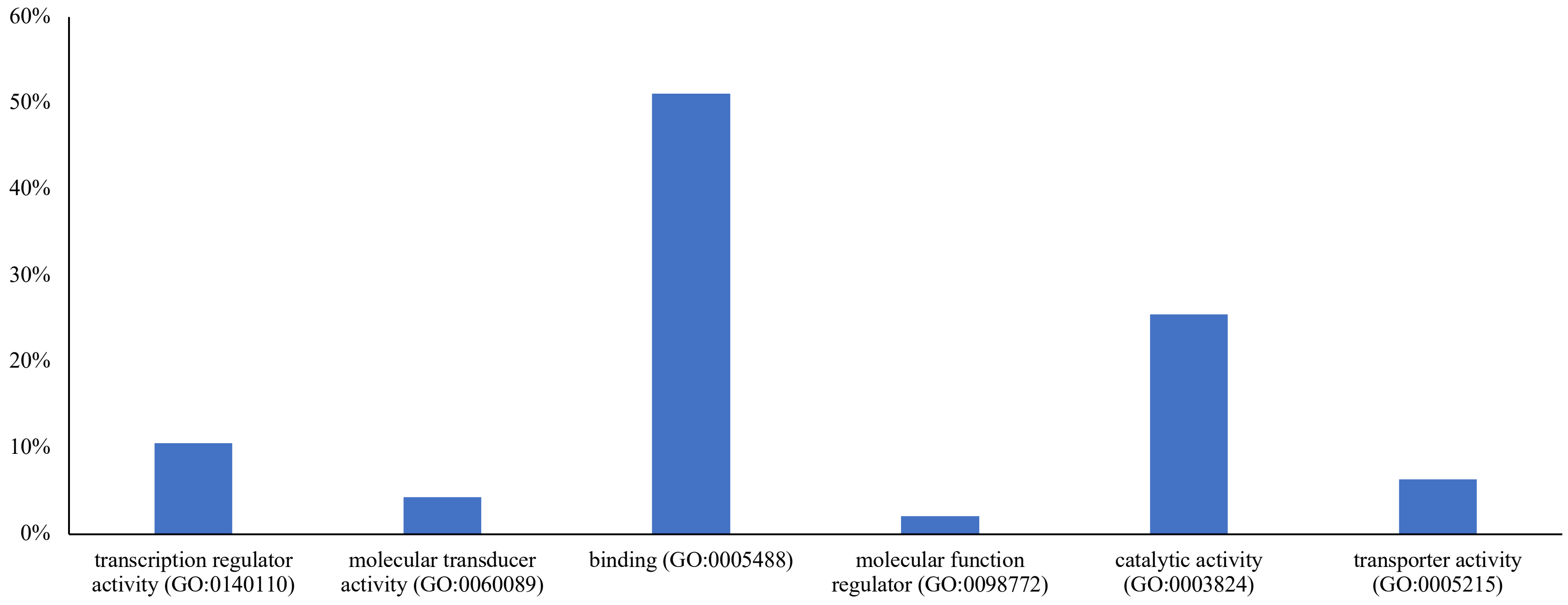

Figure S6. Schematic representation of the molecular functions (PANTHER Classification System) and the proportion of candidate genes identified in the genome-wide association analyses for the slope of the genomic reaction norms based on reproductive traits in four maternal-line pig populations.

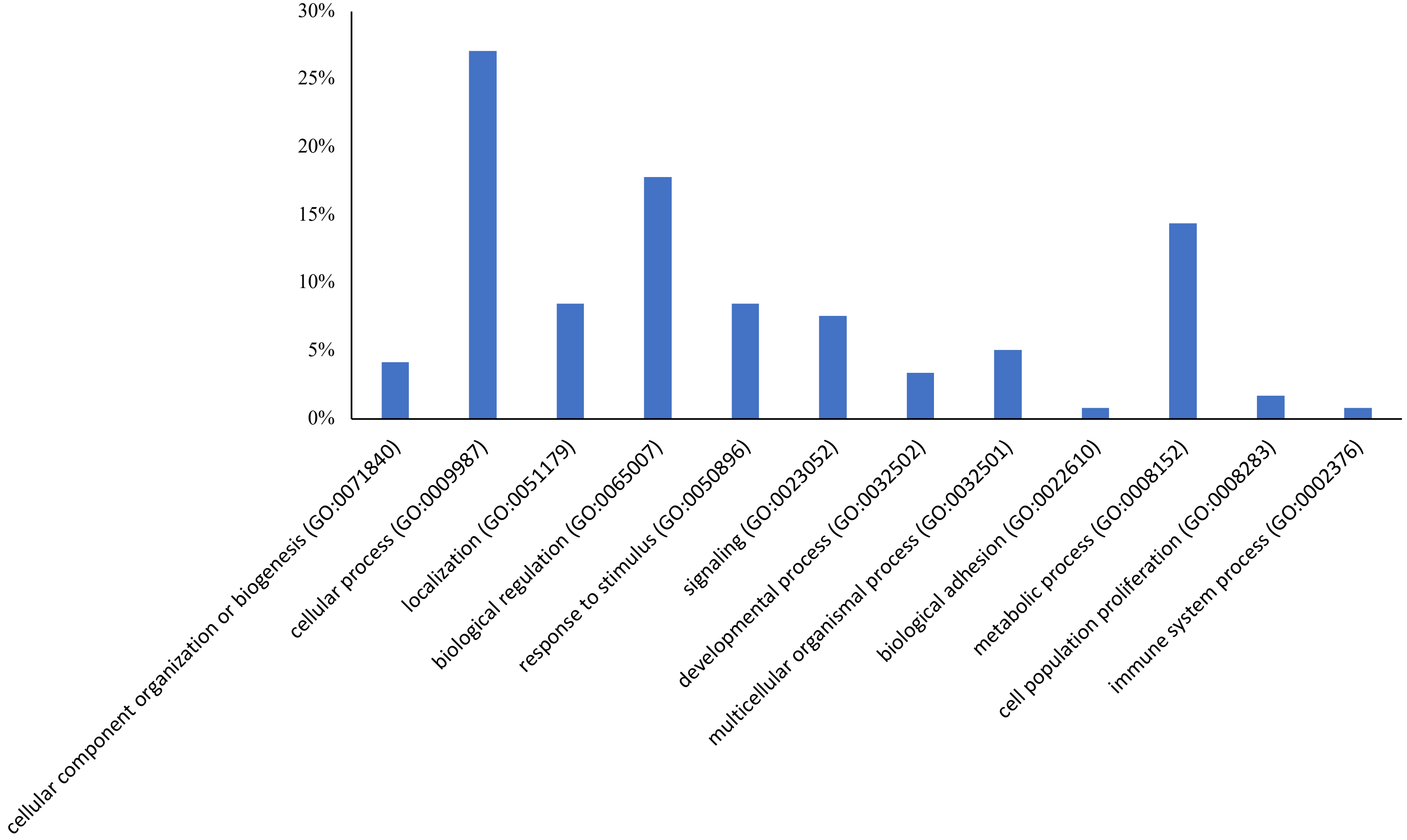

Figure S7. Schematic representation of the biological processes (PANTHER Classification System) and the proportion of candidate genes identified in the genome-wide association analyses for the slope of the genomic reaction norms based on reproductive traits in four maternal-line pig populations.

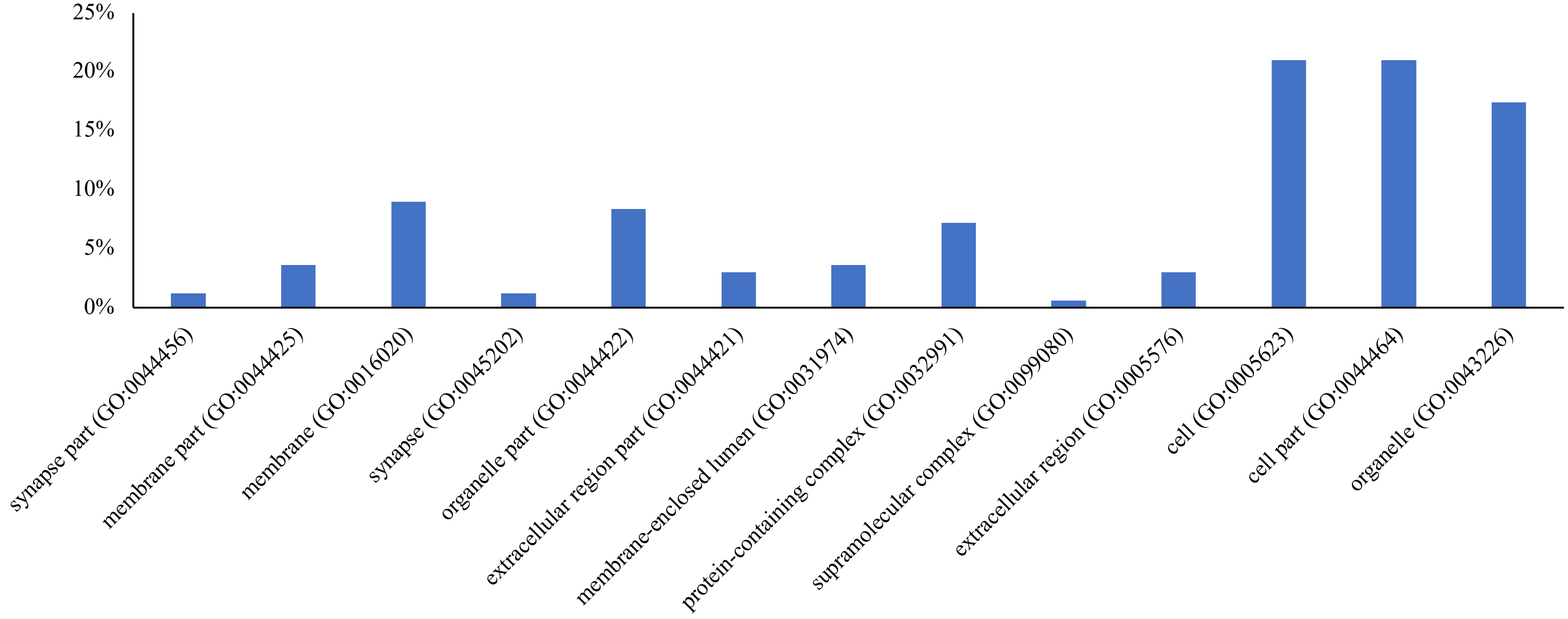

Figure S8. Schematic representation of the cellular components (PANTHER Classification System) and the proportion of candidate genes identified in the genome-wide association analyses for the slope of the genomic reaction norms based on reproductive traits in four maternal-line pig populations.
