# Supplementary material for: Genomics of Heat Tolerance in Reproductive Performance Investigated in Four Independent Maternal Lines of Pigs
Source: Front Genet. 2020 Jun 30;11:629. doi: 10.3389/fgene.2020.00629 (PMC7338773; doi:10.3389/fgene.2020.00629)
Supplement: Figure S1a — Additive genetic variance estimates (posterior means with 95% empirical confidence intervals) for total number of piglets born (TNB) in the Smithfield Premium Genetics Landrace population (SPG_LR) over the range of Average Relative Humidity 14 to 7 days before conception. [file Data_Sheet_1.zip › Supplementary files/SupplementaryTableS3b.pdf]

Table S3b: Significant windows for slope term on number of piglets born alive (NBA) as regressed on average THI 86 to 93 days into pregnancy in the Landrace population of the Maschhoffs breeding company (TML_LR).

Location	Var(%) and N SNPs in brackets	Positional genes	QTL traits
SSC12: 15,953,277-17,487,606	0.45(32)	*ENSSSCG00000017299 (MARCH10), ENSSSCG00000017300 (MRC2), ENSSSCG00000017301 (TLK2), ENSSSCG00000033421, ENSSSCG00000017304 (EFCAB3), ENSSSCG00000051573, ENSSSCG00000045518, ENSSSCG00000047329, ENSSSCG00000049121, ENSSSCG00000017305, ENSSSCG00000017306, ENSSSCG00000017307 (MYL4), ENSSSCG00000017308 (CDC27), ENSSSCG00000017310 (KANSL1), ENSSSCG00000017311 (MAPT), ENSSSCG00000043989, ENSSSCG00000017314 (SPPL2C), ENSSSCG00000017313, ENSSSCG00000031893, ENSSSCG00000017316 (NSF), ENSSSCG00000031561*	Red blood cell count, hematocrit, loin muscle area, loin pH 24 hours *post-mortem*
SSC9: 28,988,041-29,535,913	0.39(10)	*ENSSSCG00000039906, ENSSSCG00000020347, ENSSSCG00000048478*	Litter weight, hind leg conformation
SSC5: 5,425,963-5,866,100	0.34(21)	*ENSSSCG00000038685 (MPPED1), ENSSSCG00000051106, ENSSSCG00000000029 (SCUBE1), ENSSSCG00000000034 (TTLL12), ENSSSCG00000000033, ENSSSCG00000000031 (MCAT), ENSSSCG00000042788, ENSSSCG00000046130, ENSSSCG00000000035 (TTLL1), ENSSSCG00000048701, ENSSSCG00000000036 (PACSIN2)*	Hind feet conformation, corpus luteum number
SSC6: 23,525,525-23,900,710	0.34(16)	*ENSSSCG00000047100, ENSSSCG00000018823, ENSSSCG00000048963, ENSSSCG00000050597, ENSSSCG00000047287*	Obesity index, gestation length, Eicosadienoic acid content, body weight (60 days)
SSC1: 67,946,336-69,552,297	0.30(14)	*ENSSSCG00000004361 (GRIK2)*	Obesity index, backfat between 3rd and 4th last ribs
SSC2: 12,620,747-12,983,721	0.26(16)	*ENSSSCG00000046156, ENSSSCG00000034228, ENSSSCG00000050477, ENSSSCG00000039851, ENSSSCG00000031496, ENSSSCG00000048530 (OR5B3), ENSSSCG00000041440, ENSSSCG00000039026, ENSSSCG00000041067, ENSSSCG00000040949, ENSSSCG00000041818, ENSSSCG00000013162,*	Obesity index, loin pH 24 hours *post-mortem*, water holding capacity, drip loss, loin muscle

		ENSSSCG00000037988, ENSSSCG00000040200, ENSSSCG00000037952, ENSSSCG00000046773, ENSSSCG00000044981 (OR9Q2), ENSSSCG00000041862 (OR9Q1), ENSSSCG00000035278 (OR9I1)	depth, loin muscle area, pH 45 minutes *post-mortem*
SSC1: 302,821,402-303,265,377	0.25(13)	.	Feed conversion ratio, front feet conformation
SSC6: 131,951,039-132,437,869	0.23(12)	*ENSSSCG00000003761 (ADGRL2)*	.
SSC9: 29,872,932-30,447,236	0.22(10)	*ENSSSCG00000043295*	.
SSC3: 73,996,122-74,404,095	0.22(10)	*ENSSSCG00000008348 (PLEK), ENSSSCG00000036115, ENSSSCG00000008352 (PNO1), ENSSSCG00000008353 (WDR92), ENSSSCG00000039331, ENSSSCG00000019420, ENSSSCG00000047894, ENSSSCG00000047644*	*Salmonella* shedding status, CD8-positive leukocyte percentage, CD8-negative leukocyte percentage, CD3-negative
SSC5: 109,793,126-110,108,812	0.21(10)	.	.
SSC8: 72,814,981-73,286,056	0.21(21)	*ENSSSCG00000046008, ENSSSCG00000036445, ENSSSCG00000049617, ENSSSCG00000008989 (CNOT6L)*	Front leg conformation, mean corpuscular volume
SSC8: 116,174,773-116,913,617	0.20(11)	*ENSSSCG00000021764 (ARHGEF38), ENSSSCG00000022788 (PPA2), ENSSSCG00000009157 (TET2), ENSSSCG00000044596*	.
SSC6: 14,710,455-15,125,392	0.20(14)	*ENSSSCG00000002739 (PHLPP2), ENSSSCG00000002740 (AP1G1), ENSSSCG00000019395 (SNORD71), ENSSSCG00000035474, ENSSSCG00000038917 (ATXN1L), ENSSSCG00000002743 (IST1), ENSSSCG00000002741 (ZNF821), ENSSSCG00000002746 (DHODH), ENSSSCG00000002749, ENSSSCG00000050689, ENSSSCG00000025218 (TXNL4B), ENSSSCG00000002748 (DHX38), ENSSSCG00000002747 (PMFBP1)*	Unsaturated fatty acid content

SSC17: 3,329,329-3,907,409	0.19(11)	*ENSSSCG00000021988 (TUSC3), ENSSSCG00000041353, ENSSSCG00000038686, ENSSSCG00000006979 (MSR1), ENSSSCG00000044505*	.
SSC5: 6,513,590-6,784,210	0.19(10)	*ENSSSCG00000032171 (TCF20), ENSSSCG00000000046, ENSSSCG00000035064, ENSSSCG00000045281, ENSSSCG00000043669, ENSSSCG00000000045 (NDUFA6), ENSSSCG00000020945 (SMDT1), ENSSSCG00000000050, ENSSSCG00000038492 (PHETA2), ENSSSCG00000000040 (SEPTIN3), ENSSSCG00000038658, ENSSSCG00000035991 (CENPM), ENSSSCG00000039074 (TNFRSF13C), ENSSSCG00000035755 (SHISA8), ENSSSCG00000026092 (SREBF2), ENSSSCG00000035565 (CCDC134)*	Intramuscular fat content
