# Supplementary material for: Genomics of Heat Tolerance in Reproductive Performance Investigated in Four Independent Maternal Lines of Pigs
Source: Front Genet. 2020 Jun 30;11:629. doi: 10.3389/fgene.2020.00629 (PMC7338773; doi:10.3389/fgene.2020.00629)
Supplement: Figure S1a — Additive genetic variance estimates (posterior means with 95% empirical confidence intervals) for total number of piglets born (TNB) in the Smithfield Premium Genetics Landrace population (SPG_LR) over the range of Average Relative Humidity 14 to 7 days before conception. [file Data_Sheet_1.zip › Supplementary files/SupplementaryTableS3a.pdf]

Table S3a: Significant windows for intercept term on number of piglets born alive (NBA) as regressed on average THI 86 to 93 days into pregnancy in the Landrace population of the Maschhoffs breeding company (TML_LR).

Location	Var(%) and N SNPs in brackets	Positional genes	QTL traits
SSC1: 67,951,385-71,151,381	0.47(37)	*ENSSSCG00000004361 (GRIK2), ENSSSCG00000046266, ENSSSCG00000047827, ENSSSCG00000041890, ENSSSCG00000004364 (HACE1)*	Obesity index, backfat between 3rd and 4th last ribs, CD4-positive
SSC11: 79,931,895-80,427,841	0.34(17)	.	Hind leg conformation, platelet count
SSC1: 266,158-2,322,507	0.34(38)	*ENSSSCG00000043002, ENSSSCG00000050193, ENSSSCG00000046031, ENSSSCG00000042397, ENSSSCG00000035944, ENSSSCG00000047845, ENSSSCG00000004010 (ERMARD), ENSSSCG00000004009 (TCTE3), ENSSSCG00000030155 (PHF10), ENSSSCG00000038931 (C6orf120), ENSSSCG00000004008, ENSSSCG00000045488, ENSSSCG00000048735, ENSSSCG00000049501, ENSSSCG00000004012 (THBS2), ENSSSCG00000035661, ENSSSCG00000032192, ENSSSCG00000044353, ENSSSCG00000041157, ENSSSCG00000050693, ENSSSCG00000042085, ENSSSCG00000045916, ENSSSCG00000004013 (SMOC2), ENSSSCG00000043714, ENSSSCG00000031703 (DACT2), ENSSSCG00000044594, ENSSSCG00000048580, ENSSSCG00000004017, ENSSSCG00000049642, ENSSSCG00000004016, ENSSSCG00000004018 (AFDN), ENSSSCG00000038097, ENSSSCG00000047676, ENSSSCG00000047753, ENSSSCG00000051018, ENSSSCG00000049134, ENSSSCG00000045280, ENSSSCG00000043317, ENSSSCG00000051690, ENSSSCG00000027404 (UNC93A), ENSSSCG00000051474, ENSSSCG00000022580,*	Arachidonic acid to dihomo-gamma-linolenic acid ratio, Arachidonic acid to eicosadienoic acid ratio, backfat at rump, drip loss

		ENSSSCG00000032403, ENSSSCG00000037137, ENSSSCG00000024562 (CCR6), ENSSSCG00000034196, ENSSSCG00000004023 (FGFR1OP), ENSSSCG00000043814, ENSSSCG00000004024, ENSSSCG00000048164, ENSSSCG00000043922, ENSSSCG00000004022 (RPS6KA2)	
SSC9: 43,064,592-43,794,001	0.34(17)	*ENSSSCG00000051653, ENSSSCG00000049753, ENSSSCG00000041196*	.
SSC2: 19,873,884-20,805,003	0.33(16)	*ENSSSCG00000047796, ENSSSCG00000047142, ENSSSCG00000045282, ENSSSCG00000042424*	Body depth, rib shape, coping behavior
SSC8: 871,763-1,716,574	0.33(20)	*ENSSSCG00000030827 (FGFR3), ENSSSCG00000008675 (LETM1), ENSSSCG00000051354, ENSSSCG00000049380, ENSSSCG00000008682 (NSD2), ENSSSCG00000020173 (SCARNA22), ENSSSCG00000008681 (NELFA), ENSSSCG00000008686, ENSSSCG00000033541 (NAT8L), ENSSSCG00000008684 (POLN), ENSSSCG00000027718 (HAUS3), ENSSSCG00000008687 (MXD4), ENSSSCG00000008689 (ZFYVE28), ENSSSCG00000034633 (CFAP99), ENSSSCG00000008690 (RNF4), ENSSSCG00000029745 (FAM193A), ENSSSCG00000008691 (TNIP2), ENSSSCG00000022004 (SH3BP2), ENSSSCG00000044393, ENSSSCG00000008692 (ADD1), ENSSSCG00000008695 (MFSD10), ENSSSCG00000008693 (NOP14)*	Days to 115 kg
SSC1: 71,950,726-73,330,274	0.32(22)	*ENSSSCG00000042384, ENSSSCG00000045922, ENSSSCG00000034393, ENSSSCG00000049688, ENSSSCG00000043520, ENSSSCG00000004370 (ATG5), ENSSSCG00000004369 (PRDM1), ENSSSCG00000004371 (CRYBG1), ENSSSCG00000035339, ENSSSCG00000046528, ENSSSCG00000041504, ENSSSCG00000004373 (RTN4IP1), ENSSSCG00000004374 (QRSL1), ENSSSCG00000049906, ENSSSCG00000043597, ENSSSCG00000031023, ENSSSCG00000037071, ENSSSCG00000037985 (MTRES1), ENSSSCG00000031159, ENSSSCG00000036168 (PDSS2)*	CD4-positive, time spent lying

SSC8: 4,941-465,122	0.32(13)	*ENSSSCG00000047999, ENSSSCG00000039991 (PIGG), ENSSSCG00000043358 (TMEM271), ENSSSCG00000036645 (PDE6B), ENSSSCG00000023903, ENSSSCG00000027502 (MYL5), ENSSSCG00000026084 (SLC49A3), ENSSSCG00000019544, ENSSSCG00000029983 (PCGF3), ENSSSCG00000037527 (CPLX1), ENSSSCG00000046122, ENSSSCG00000021289 (GAK), ENSSSCG00000038234 (TMEM175), ENSSSCG00000027286 (DGKQ), ENSSSCG00000032262 (IDUA), ENSSSCG00000035739 (SLC26A1), ENSSSCG00000036512 (FGFRL1), ENSSSCG00000045703, ENSSSCG00000032808, ENSSSCG00000039488 (SPON2)*	.
SSC2: 7,190,662-7,921,973	0.32(16)	*ENSSSCG00000023178 (BATF2), ENSSSCG00000040674, ENSSSCG00000013015 (GPHA2), ENSSSCG00000013016 (PPP2R5B), ENSSSCG00000023890 (ATG2A), ENSSSCG00000045555, ENSSSCG00000019906, ENSSSCG00000046741, ENSSSCG00000018349, ENSSSCG00000013017 (EHD1), ENSSSCG00000013018 (CDC42BPG), ENSSSCG00000013019 (MEN1), ENSSSCG00000013020 (MAP4K2), ENSSSCG00000013022 (PYGM), ENSSSCG00000013023, ENSSSCG00000033229 (NRXN2), ENSSSCG00000051519, ENSSSCG00000013024, ENSSSCG00000013025 (SLC22A12), ENSSSCG00000020831 (SLC22A11), ENSSSCG00000043801, ENSSSCG00000050721, ENSSSCG00000044804, ENSSSCG00000028806 (RPS6KA4), ENSSSCG00000013042, ENSSSCG00000013030, ENSSSCG00000013029 (TRMT112), ENSSSCG00000013028 (ESRRA), ENSSSCG00000013027, ENSSSCG00000013031 (KCNK4), ENSSSCG00000013032 (GPR137), ENSSSCG00000013033 (BAD), ENSSSCG00000013034 (PLCB3), ENSSSCG00000037999, ENSSSCG00000033757, ENSSSCG00000013038, ENSSSCG00000013039 (NUDT22), ENSSSCG00000013040 (TRPT1), ENSSSCG00000013041 (FERMT3), ENSSSCG00000021620 (STIP1)*	Average daily gain, loin and neck weight, backfat at rump
SSC1: 65,183,502-66,336,428	0.31(21)	*ENSSSCG00000042619, ENSSSCG00000051730, ENSSSCG00000047707, ENSSSCG00000044402, ENSSSCG00000047909, ENSSSCG00000049009,*	Conductivity 45 minutes post-mortem, mummified

		ENSSSCG00000018969, ENSSSCG00000044799, ENSSSCG00000046887, ENSSSCG00000047992, ENSSSCG00000043225, ENSSSCG00000041445, ENSSSCG00000049318, ENSSSCG00000004347 (FBXL4), ENSSSCG00000004349 (FAXC), ENSSSCG00000037594 (PNISR)	pigs, maternal infanticide, Hemoglobin
SSC3: 141,963,034-142,766,769	0.29(16)	.	.
SSC8: 140,234,308-140,885,162	0.29(22)	.	Body weight (birth), front leg conformation, front feet conformation
SSC13: 32,626,695-33,219,455	0.28(19)	*ENSSSCG00000011398 (SEMA3F), ENSSSCG00000042718 (GNAT1), ENSSSCG00000011397 (SLC38A3), ENSSSCG00000011399 (GNAI2), ENSSSCG00000011400 (SEMA3B), ENSSSCG00000011401 (LSMEM2), ENSSSCG00000011402 (IFRD2), ENSSSCG00000041673, ENSSSCG00000024363 (HYAL3), ENSSSCG00000011404, ENSSSCG00000011405 (HYAL2), ENSSSCG00000011407 (RASSF1), ENSSSCG00000011408 (ZMYND10), ENSSSCG00000011410 (CYB561D2), ENSSSCG00000043195 (TMEM115), ENSSSCG00000011412 (CACNA2D2), ENSSSCG00000037433 (C3orf18), ENSSSCG00000025272 (HEMK1), ENSSSCG00000048288, ENSSSCG00000050226, ENSSSCG00000040581, ENSSSCG00000011415 (MAPKAPK3), ENSSSCG00000011416 (DOCK3)*	Firmness, hip structure, front leg conformation, hind leg conformation
SSC1: 191,857,174-193,873,995	0.28(17)	*ENSSSCG00000005106 (NTRK3), ENSSSCG00000044241, ENSSSCG00000050818, ENSSSCG00000041527, ENSSSCG00000043508, ENSSSCG00000045893, ENSSSCG00000036551, ENSSSCG00000051349, ENSSSCG00000048252, ENSSSCG00000045398, ENSSSCG00000042933, ENSSSCG00000044158, ENSSSCG00000005109 (ESR2)*	Feed conversion ratio, residual feed intake, intramuscular fat content, number of stillborn

SSC1: 185,048,329-185,734,190	0.28(17)	*ENSSSCG00000005060 (KTN1), ENSSSCG00000033800 (PELI2), ENSSSCG00000050996*	Obesity index
SSC16: 37,733,559-39,019,226	0.28(18)	*ENSSSCG00000043299, ENSSSCG00000016929 (PDE4D), ENSSSCG00000046737, ENSSSCG00000018875*	Days to 100 kg, feed conversion ratio, Arachidic acid to stearic acid ratio, loin muscle area, Arachidic acid content, Eicosenoic acid to eicosanoic acid ratio, coping behavior
SSC1: 182,684,682-183,880,998	0.27(17)	*ENSSSCG00000005043 (DDHD1), ENSSSCG00000051102, ENSSSCG00000041305, ENSSSCG00000005045 (BMP4), ENSSSCG00000005047 (CDKN3), ENSSSCG00000027256 (CNIH1), ENSSSCG00000026294 (GMFB), ENSSSCG00000021451 (CGRRF1)*	Obesity index
SSC6: 71,960,401-72,949,935	0.27(24)	*ENSSSCG00000028965, ENSSSCG00000003432 (KIAA2013), ENSSSCG00000003433 (PLOD1), ENSSSCG00000003434 (MFN2), ENSSSCG00000003435 (MIIP), ENSSSCG00000022208 (TNFRSF1B), ENSSSCG00000003438 (VPS13D), ENSSSCG00000020322, ENSSSCG00000041182 (SNORA59A), ENSSSCG00000048520, ENSSSCG00000003439 (DHRS3), ENSSSCG00000049829, ENSSSCG00000003440 (AADACL4), ENSSSCG00000003441 (AADACL3), ENSSSCG00000045525, ENSSSCG00000003442 (C1orf158), ENSSSCG00000003444, ENSSSCG00000033480, ENSSSCG00000033129, ENSSSCG00000033821, ENSSSCG00000037747, ENSSSCG00000031836, ENSSSCG00000044717, ENSSSCG00000032991, ENSSSCG00000039417, ENSSSCG00000040680, ENSSSCG00000037632, ENSSSCG00000003449 (LRRC38)*	Age at puberty, CD3-negative, Stearic acid content, litter size, drip loss, impedance 24 hours post-mortem, pH 45 minutes *post-mortem*
SSC5: 71,774,499-72,424,166	0.25(14)	*ENSSSCG00000045210, ENSSSCG00000044708, ENSSSCG00000000784, ENSSSCG00000047044, ENSSSCG00000041614, ENSSSCG00000000789 (CNTN1)*	Dressing percentage

SSC6: 119,172,911-121,684,388	0.23(38)	*ENSSSCG00000030018 (ZNF396), ENSSSCG00000038859 (INO80C), ENSSSCG00000003743 (GALNT1), ENSSSCG00000030002 (C18orf21), ENSSSCG00000027700 (RPRD1A), ENSSSCG00000035019, ENSSSCG00000027455 (SLC39A6), ENSSSCG00000020898 (ELP2), ENSSSCG00000003744 (MOCOS), ENSSSCG00000027935 (FHOD3), ENSSSCG00000043137, ENSSSCG00000003746, ENSSSCG00000026953 (TPGS2), ENSSSCG00000042283, ENSSSCG00000034886, ENSSSCG00000003747 (KIAA1328), ENSSSCG00000047288, ENSSSCG00000045019, ENSSSCG00000003748 (CELF4), ENSSSCG00000042833, ENSSSCG00000045034*	Intramuscular fat content, litter size, backfat at 10th rib, pH 45 minutes *post-mortem*, cervical vertebra length, Docosapentaenoic acid content
SSC12: 879,362-1,516,612	0.23(15)	*ENSSSCG00000029553, ENSSSCG00000029944, ENSSSCG00000040352 (DUS1L), ENSSSCG00000033689 (GPS1), ENSSSCG00000040385 (RFNG), ENSSSCG00000031851, ENSSSCG00000039873 (DCXR), ENSSSCG00000036973 (RAC3), ENSSSCG00000040503 (LRRC45), ENSSSCG00000049515, ENSSSCG00000040727 (ASPSCR1), ENSSSCG00000039013 (NOTUM), ENSSSCG00000044793, ENSSSCG00000032974 (MYADML2), ENSSSCG00000034723 (PYCR1), ENSSSCG00000033148 (MRPL12), ENSSSCG00000035253 (MAFG), ENSSSCG00000034695 (SIRT7), ENSSSCG00000039650 (PCYT2), ENSSSCG00000032655 (ALYREF), ENSSSCG00000035837, ENSSSCG00000035728, ENSSSCG00000034058 (ARHGDIA), ENSSSCG00000039148 (P4HB), ENSSSCG00000037039 (PPP1R27), ENSSSCG00000039424 (MCRIP1), ENSSSCG00000034891, ENSSSCG00000028355 (ACTG1), ENSSSCG00000033620, ENSSSCG00000033082 (HGS), ENSSSCG00000034716 (CCDC137), ENSSSCG00000033788 (OXLD1), ENSSSCG00000045526, ENSSSCG00000031531 (TSPAN10), ENSSSCG00000024235 (NPLOC4), ENSSSCG00000029616 (FAAP100), ENSSSCG00000026155 (FSCN2), ENSSSCG00000051223, ENSSSCG00000023045, ENSSSCG00000048652,*	Palmitic acid to myristic acid ratio, Myristic acid content, Oleic acid to palmitoleic acid ratio

		ENSSSCG00000022317 (SLC38A10), ENSSSCG00000039183 (NDUFAF8)	
SSC3: 11,346,527-11,643,257	0.22(13)	*ENSSSCG00000025966, ENSSSCG00000027487 (LAT2), ENSSSCG00000028504 (RFC2), ENSSSCG00000007719 (CLIP2), ENSSSCG00000007720 (GTF2IRD1)*	.
SSC18: 52,862,142-53,233,305	0.22(12)	*ENSSSCG00000050225, ENSSSCG00000044824, ENSSSCG00000035077, ENSSSCG00000034662, ENSSSCG00000045227*	.
SSC6: 75,839,337-76,941,199	0.22(22)	*ENSSSCG00000003481 (PADI1), ENSSSCG00000003482 (PADI3), ENSSSCG00000003483 (PADI4), ENSSSCG00000003484 (PADI6), ENSSSCG00000003485 (RCC2), ENSSSCG00000003486 (ARHGEF10L), ENSSSCG00000046919, ENSSSCG00000024062 (ACTL8), ENSSSCG00000043698, ENSSSCG00000051351, ENSSSCG00000020992 (IGSF21)*	Oleic acid content, Monounsaturated fatty acid content, Monounsaturated fatty acid to saturated fatty acid ratio, Palmitoleic acid content, Linoleic acid content, age at puberty, intramuscular fat content, backfat at last rib, cis-Vaccenic acid content
SSC16: 4,863,778-5,304,243	0.22(13)	*ENSSSCG00000028239 (FBXL7)*	Myristic acid content, hind leg conformation
SSC1: 199,936,889-201,064,583	0.21(14)	*ENSSSCG00000050021, ENSSSCG00000005131 (DMRTA1), ENSSSCG00000050165, ENSSSCG00000034120 (CDKN2B), ENSSSCG00000005134, ENSSSCG00000005135 (MTAP), ENSSSCG00000041931, ENSSSCG00000045977*	Conductivity 45 minutes *post-mortem*, external fat on ham, adipocyte diameter
SSC6: 21,846,594-22,591,224	0.21(16)	*ENSSSCG00000043338, ENSSSCG00000047750, ENSSSCG00000045327*	Body length, drip loss, loin pH 24 hours *post-mortem*, average daily gain
SSC9: 64,410,416-65,286,501	0.21(16)	*ENSSSCG00000015273 (ATP2B4), ENSSSCG00000033768 (LAX1), ENSSSCG00000028035, ENSSSCG00000023562, ENSSSCG00000015277 (SOX13), ENSSSCG00000015276 (ETNK2), ENSSSCG00000031235 (REN), ENSSSCG00000038350 (KISS1), ENSSSCG00000015281 (PLEKHA6), ENSSSCG00000020589,*	Coping behavior

		ENSSSCG00000048468, ENSSSCG00000015282 (PPP1R15B), ENSSSCG00000015283 (PIK3C2B), ENSSSCG00000015284 (MDM4), ENSSSCG00000034803, ENSSSCG00000015285 (LRRN2)	
SSC8: 16,971,073-17,295,697	0.21(11)	*ENSSSCG00000008754 (GBA3)*	Litter size, birth weight variability
SSC6: 89,485,894-90,353,325	0.21(11)	*ENSSSCG00000050185, ENSSSCG00000032078 (ZNF362), ENSSSCG00000032224 (A3GALT2), ENSSSCG00000025344 (PHC2), ENSSSCG00000003621 (ZSCAN20), ENSSSCG00000042252, ENSSSCG00000003622, ENSSSCG00000047402, ENSSSCG00000049113, ENSSSCG00000021247 (HMGB4)*	Backfat at rump, backfat at mid-back
SSC16: 40,635,936-41,985,204	0.21(14)	*ENSSSCG00000046595, ENSSSCG00000030186 (KIF2A), ENSSSCG00000031990 (DIMT1), ENSSSCG00000016938 (IPO11), ENSSSCG00000020338, ENSSSCG00000037608 (LRRC70), ENSSSCG00000048063, ENSSSCG00000020281, ENSSSCG00000046520, ENSSSCG00000043802, ENSSSCG00000049006*	Arachidic acid to stearic acid ratio, Cis-11-Eicosenoic acid to oleic acid ratio, Eicosenoic acid to eicosanoic acid ratio, Loin muscle area, Loin muscle depth, Arachidic acid content
SSC9: 24,642,230-25,414,898	0.20(13)	*ENSSSCG00000014935*	Intramuscular fat content, Hind leg conformation
SSC9: 45,356,730-45,626,011	0.20(11)	*ENSSSCG00000015085 (IL10RA), ENSSSCG00000050318 (SMIM35), ENSSSCG00000015086 (TMPRSS4), ENSSSCG00000029199, ENSSSCG00000021651 (SCN2B), ENSSSCG00000015089 (JAML), ENSSSCG00000015090 (MPZL3), ENSSSCG00000015091, ENSSSCG00000040140*	Liver weight
SSC11: 244-307,678	0.20(10)	*ENSSSCG00000047805, ENSSSCG00000022638 (ATP12A), ENSSSCG00000049484, ENSSSCG00000009572 (RNF17), ENSSSCG00000029393 (CENPJ)*	Total number of piglets born alive
SSC2: 5,034,464-5,479,542	0.20(12)	*ENSSSCG00000012903 (PITPNM1), ENSSSCG00000012904 (AIP), ENSSSCG00000044979, ENSSSCG00000012905 (TMEM134), ENSSSCG00000012906 (CABP4), ENSSSCG00000033777 (GPR152),*	Average backfat thickness, backfat at last lumbar,

		ENSSSCG00000012920 (CORO1B), ENSSSCG00000012909 (PTPRCAP), ENSSSCG00000012910 (RPS6KB2), ENSSSCG00000012911 (CARNS1), ENSSSCG00000012912 (TBC1D10C), ENSSSCG00000012913, ENSSSCG00000012914 (RAD9A), ENSSSCG00000043645, ENSSSCG00000012915, ENSSSCG00000012916, ENSSSCG00000048979, ENSSSCG00000027956 (SSH3), ENSSSCG00000012917 (ANKRD13D), ENSSSCG00000012918 (GRK2), ENSSSCG00000029637 (KDM2A), ENSSSCG00000022726 (RHOD), ENSSSCG00000024837 (SYT12), ENSSSCG00000040729 (C11orf86), ENSSSCG00000049464	backfat at tenth rib, loin pH 24 hours *post-mortem*
SSC7: 118,891,334-119,330,129	0.19(14)	*ENSSSCG00000041330, ENSSSCG00000046605, ENSSSCG00000042232*	Cortisol level
SSC11: 83,965,860-84,319,272	0.19(10)	.	.
SSC1: 184,125,931-184,917,301	0.19(11)	*ENSSSCG00000023408 (SAMD4A), ENSSSCG00000042101, ENSSSCG00000049819, ENSSSCG00000035715 (GCH1), ENSSSCG00000005052 (WDHD1), ENSSSCG00000032332 (SOCS4), ENSSSCG00000005054 (MAPK1IP1L), ENSSSCG00000005055 (LGALS3), ENSSSCG00000005056 (DLGAP5), ENSSSCG00000005057 (FBXO34), ENSSSCG00000047403, ENSSSCG00000005058 (ATG14), ENSSSCG00000005059 (TBPL2), ENSSSCG00000019668, ENSSSCG00000005060 (KTN1)*	Obesity index
SSC3: 140,363,332-141,033,116	0.19(12)	.	.
SSC1: 3,560,483-4,058,074	0.19(11)	*ENSSSCG00000044274, ENSSSCG00000049643*	.
SSC18: 24,758,016-25,209,980	0.19(12)	*ENSSSCG00000016611 (CADPS2), ENSSSCG00000031221 (FEZF1), ENSSSCG00000016613 (AASS), ENSSSCG00000016614 (PTPRZ1)*	Front feet conformation

SSC4: 95,686,177-96,114,909	0.18(11)	*ENSSSCG00000038125 (NUP210L), ENSSSCG00000031838 (RPS27), ENSSSCG00000006564, ENSSSCG00000006559, ENSSSCG00000006560 (CREB3L4), ENSSSCG00000006561 (SLC39A1), ENSSSCG00000006567 (CRTC2), ENSSSCG00000006568 (DENND4B), ENSSSCG00000006562 (GATAD2B), ENSSSCG00000050567, ENSSSCG00000050118, ENSSSCG00000006571 (INTS3), ENSSSCG00000006569 (SLC27A3), ENSSSCG00000043560, ENSSSCG00000031753, ENSSSCG00000006572 (NPR1), ENSSSCG00000028553 (ILF2), ENSSSCG00000034159 (SNAPIN), ENSSSCG00000031053 (S100A1), ENSSSCG00000022107 (CHTOP), ENSSSCG00000038991, ENSSSCG00000006582, ENSSSCG00000006581, ENSSSCG00000006580 (S100A2), ENSSSCG00000006579 (S100A3), ENSSSCG00000006578 (S100A4), ENSSSCG00000006577 (S100A5), ENSSSCG00000021084 (S100A6)*	Litter size, total number of piglets born alive
SSC9: 24,155,728-24,432,100	0.18(10)	*ENSSSCG00000041757, ENSSSCG00000041340*	.
SSC1: 63,025,137-63,236,510	0.18(10)	.	Palmitic acid content, saturated fatty acid content, unsaturated fatty acid content
SSC1: 61,534,580-62,058,511	0.17(10)	*ENSSSCG00000050706*	Dressing percentage, fat androstenone level, mummified pigs
SSC8: 14,413,402-14,705,207	0.17(10)	.	.
SSC15: 150,448,862-150,829,050	0.17(10)	.	.

SSC9: 19,155,863-19,459,321	0.17(10)	*ENSSSCG00000014904 (DLG2), ENSSSCG00000050112, ENSSSCG00000049439*	Litter size

Location: indicates the chromosome number (SSC1 to SSC18), followed by the starting and end position of the genomic window(s) in base pairs; **Var(%)**: the percental variance explained by the most relevant 10-SNP window within the region of interest; **N SNP:** number of SNP marker included in the genomic region identified; **Positional genes:** Ensembl gene stable ID (with official gene symbol when known) mapped to the genomic regions identified; **QTL traits**: indicates which traits have known QTLs mapped within the genomic region of interest, based on QTLs reported in the Pig QTL database (https://www.animalgenome.org/cgi-bin/QTLdb/SS/index).
