# Supplementary material for: Genomics of Heat Tolerance in Reproductive Performance Investigated in Four Independent Maternal Lines of Pigs
Source: Front Genet. 2020 Jun 30;11:629. doi: 10.3389/fgene.2020.00629 (PMC7338773; doi:10.3389/fgene.2020.00629)
Supplement: Figure S1a — Additive genetic variance estimates (posterior means with 95% empirical confidence intervals) for total number of piglets born (TNB) in the Smithfield Premium Genetics Landrace population (SPG_LR) over the range of Average Relative Humidity 14 to 7 days before conception. [file Data_Sheet_1.zip › Supplementary files/SupplementaryTableS1.pdf]

Table S1: Summary of the environmental covariates tested in this study. Each row in the table reports a summary of the 19 weekly values for the population. **Population:** indicates the breeding company and breed, in which **SPG**: Smithfield Premium Genetics, **TML**: The Maschhoffs, **LR**: Landrace, **LW**: Large White, and **YS**: Yorkshire (also known as Large White in other breeding companies). **Average Mean:** the mean of the environmental covariate was averaged. **Average SD:** the standard deviation of the environmental covariate was averaged. **Autocorrelation:** correlation between the environmental covariate values at week *t* and week *t+1*.

Population	Variable	Average Mean	Average SD	Autocorrelation
SPG_LR	Average Temperature	16.10	8.84	0.92
SPG_LR	Maximum temperature	22.79	8.94	0.89
SPG_LR	Minimum Temperature	9.14	8.91	0.93
SPG_LR	Relative Humidity	53.58	14.39	0.75
SPG_LR	THI	60.23	11.92	0.92
SPG_LW	Average Temperature	16.05	8.90	0.92
SPG_LW	Maximum temperature	22.79	8.95	0.90
SPG_LW	Minimum Temperature	9.03	9.03	0.93
SPG_LW	Relative Humidity	51.44	14.40	0.73
SPG_LW	THI	60.07	11.87	0.91
TML_LR	Average Temperature	12.80	9.73	0.92
TML_LR	Maximum temperature	18.80	10.17	0.90
TML_LR	Minimum Temperature	6.53	9.52	0.92
TML_LR	Relative Humidity	67.17	9.60	0.66
TML_LR	THI	55.64	14.53	0.92
TML_YS	Average Temperature	12.01	9.75	0.92
TML_YS	Maximum temperature	17.95	10.24	0.90
TML_YS	Minimum Temperature	5.79	9.50	0.92
TML_YS	Relative Humidity	67.41	9.52	0.66
TML_YS	THI	54.44	14.63	0.92
