# Supplementary material for: Genomics of Heat Tolerance in Reproductive Performance Investigated in Four Independent Maternal Lines of Pigs
Source: Front Genet. 2020 Jun 30;11:629. doi: 10.3389/fgene.2020.00629 (PMC7338773; doi:10.3389/fgene.2020.00629)
Supplement: Figure S1a — Additive genetic variance estimates (posterior means with 95% empirical confidence intervals) for total number of piglets born (TNB) in the Smithfield Premium Genetics Landrace population (SPG_LR) over the range of Average Relative Humidity 14 to 7 days before conception. [file Data_Sheet_1.zip › Supplementary files/SupplementaryTableS2a.pdf]

Table S2a: Significant genomic windows for the intercept term of the reaction norm on total number of piglets born (TNB) as regressed on the Average Relative Humidity 14 to 7 days before conception in the Smithfield Premium Genetics (SPG) Landrace population (SPG_LR).

Location	Var(%) and N SNPs in brackets	Positional genes	QTL traits
SSC2: 133,626,412-134,446,288	0.36(28)	*ENSSSCG00000033213 (LYRM7), ENSSSCG00000036686 (CDC42SE2), ENSSSCG00000014267, ENSSSCG00000014269 (FNIP1), ENSSSCG00000020274, ENSSSCG00000014271 (MEIKIN), ENSSSCG00000014272, ENSSSCG00000023737, ENSSSCG00000036626*	.
SSC2: 157,492,614-158,466,521	0.34(29)	.	Spinal curvature, average daily gain, pH 24 hours *post-mortem* (loin and ham), conductivity 24 hours *post-mortem*, flavor score
SSC16: 47,916,509-48,994,238	0.32(21)	*ENSSSCG00000016971 (MCCC2), ENSSSCG00000047824, ENSSSCG00000016972, ENSSSCG00000022219, ENSSSCG00000016974 (MAP1B), ENSSSCG00000023818 (MRPS27), ENSSSCG00000046085, ENSSSCG00000016975 (PTCD2), ENSSSCG00000039883, ENSSSCG00000048660, ENSSSCG00000044917, ENSSSCG00000046046, ENSSSCG00000016976 (ZNF366), ENSSSCG00000047095, ENSSSCG00000048350*	Arachidic acid to stearic acid ratio, Eicosenoic acid to eicosanoic acid ratio, average backfat thickness
SSC17: 48,537,476-49,274,453	0.31(18)	*ENSSSCG00000023306 (ELMO2), ENSSSCG00000051122, ENSSSCG00000042586, ENSSSCG00000041922, ENSSSCG00000051117, ENSSSCG00000032191, ENSSSCG00000007448 (OCSTAMP), ENSSSCG00000035906 (SLC13A3), ENSSSCG00000007452 (TP53RK), ENSSSCG00000007451 (SLC2A10), ENSSSCG00000041724, ENSSSCG00000033678, ENSSSCG00000039038, ENSSSCG00000007453 (EYA2), ENSSSCG00000039785, ENSSSCG00000007454 (ZMYND8)*	Gestation length, hind leg conformation
SSC15: 143,339,720-143,835,976	0.30(17)	.	.

SSC10: 31,882,839-32,846,771	0.29(17)	*ENSSSCG00000040707, ENSSSCG00000038162, ENSSSCG00000038502, ENSSSCG00000010966, ENSSSCG00000034888, ENSSSCG00000010968, ENSSSCG00000010967, ENSSSCG00000010969 (GALT), ENSSSCG00000037000 (SIGMAR1), ENSSSCG00000010971 (ARID3C), ENSSSCG00000010972 (DCTN3), ENSSSCG00000010973 (RPP25L), ENSSSCG00000010974 (CNTFR), ENSSSCG00000027428, ENSSSCG00000039342 (DNAI1), ENSSSCG00000033196 (MYORG), ENSSSCG00000035455 (FAM219A), ENSSSCG00000010980 (C9orf24), ENSSSCG00000010981 (NUDT2), ENSSSCG00000010984 (KIF24), ENSSSCG00000021761 (UBAP1), ENSSSCG00000024384 (DCAF12), ENSSSCG00000010987 (UBAP2), ENSSSCG00000049962*	Heart weight, mean corpuscular volume, total number of piglets born alive
SSC1: 12,797,159-13,478,197	0.29(25)	*ENSSSCG00000041027, ENSSSCG00000050641, ENSSSCG00000004075 (RGS17), ENSSSCG00000004076(MTRF1L), ENSSSCG00000004077 (FBXO5)*	Drip loss
SSC9: 15,049,063-15,566,499	0.28(19)	*ENSSSCG00000045934, ENSSSCG00000034240, ENSSSCG00000043081, ENSSSCG00000047651, ENSSSCG00000047633, ENSSSCG00000048266*	Intramuscular fat content
SSC2: 73,651,236-76,656,637	0.26(22)	*ENSSSCG00000028612 (PTPRS), ENSSSCG00000024570 (KDM4B), ENSSSCG00000013517 (UHRF1), ENSSSCG00000037183 (ARRDC5), ENSSSCG00000044528, ENSSSCG00000038954 (PLIN3), ENSSSCG00000024771 (TICAM1), ENSSSCG00000044427, ENSSSCG00000037153 (FEM1A), ENSSSCG00000046891, ENSSSCG00000045032, ENSSSCG00000026063 (DPP9), ENSSSCG00000034898 (MYDGF), ENSSSCG00000034615 (TNFAIP8L1), ENSSSCG00000035176 (SEMA6B), ENSSSCG00000013513 (PLIN5), ENSSSCG00000013512 (PLIN4), ENSSSCG00000013511 (HDGFL2), ENSSSCG00000013510 (UBXN6), ENSSSCG00000013509 (CHAF1A), ENSSSCG00000013508 (SH3GL1), ENSSSCG00000013507 (MPND), ENSSSCG00000013506 (STAP2), ENSSSCG00000013505 (FSD1), ENSSSCG00000013504, ENSSSCG00000013503 (SHD), ENSSSCG00000013499 (YJU2), ENSSSCG00000013498 (EBI3), ENSSSCG00000013497 (ANKRD24), ENSSSCG00000013496, ENSSSCG00000013501 (CREB3L3), ENSSSCG00000027127,*	Mean corpuscular volume, spinal curvature, number of mummified piglets, pH 24 hours *post-mortem* (loin), marbling, thawing loss, mean corpuscular hemoglobin content

ENSSSCG00000044761, ENSSSCG00000029236 (ZBTB7A), ENSSSCG00000022066 (PIAS4), ENSSSCG00000050591, ENSSSCG00000026884, ENSSSCG00000025675 (EEF2), ENSSSCG00000029719 (SNORD37), ENSSSCG00000032170 (DAPK3), ENSSSCG00000040860 (NMRK2), ENSSSCG00000026754 (ATCAY), ENSSSCG00000013495 (ZFR2), ENSSSCG00000013494 (MATK), ENSSSCG00000032429 (RAX2), ENSSSCG00000024242 (MRPL54), ENSSSCG00000013492 (APBA3), ENSSSCG00000013491 (TJP3), ENSSSCG00000013490 (PIP5K1C), ENSSSCG00000050486, ENSSSCG00000032085 (CACTIN), ENSSSCG00000033759 (TBXA2R), ENSSSCG00000036190 (GIPC3), ENSSSCG00000048374, ENSSSCG00000040421 (HMG20B), ENSSSCG00000026169 (MFSD12), ENSSSCG00000013481 (FZR1), ENSSSCG00000044884 (C19orf71), ENSSSCG00000013478 (DOHH), ENSSSCG00000049629, ENSSSCG00000038067, ENSSSCG00000036537 (NFIC), ENSSSCG00000013476 (CELF5), ENSSSCG00000013475 (NCLN), ENSSSCG00000013474 (S1PR4), ENSSSCG00000013473 (GNA15), ENSSSCG00000029279, ENSSSCG00000022205, ENSSSCG00000032265 (TLE5), ENSSSCG00000028995 (TLE2), ENSSSCG00000024588, ENSSSCG00000041800, ENSSSCG00000036792, ENSSSCG00000038030, ENSSSCG00000034545, ENSSSCG00000013471, ENSSSCG00000039774, ENSSSCG00000013469 (ZNF555), ENSSSCG00000013468, ENSSSCG00000038635 (ZNF554), ENSSSCG00000013467 (SGTA), ENSSSCG00000038013 (DIRAS1), ENSSSCG00000033949, ENSSSCG00000049505, ENSSSCG00000043780, ENSSSCG00000022689 (GADD45B), ENSSSCG00000026784 (LMNB2), ENSSSCG00000022757, ENSSSCG00000044920, ENSSSCG00000028363 (TMPRSS9), ENSSSCG00000013464 (SPPL2B), ENSSSCG00000013463 (LSM7), ENSSSCG00000013462 (LINGO3), ENSSSCG00000013461 (PEAK3), ENSSSCG00000013460, ENSSSCG00000013457 (DOT1L), ENSSSCG00000013453, ENSSSCG00000035812 (AMH), ENSSSCG00000031899 (JSRP1), ENSSSCG00000040157 (AP3D1), ENSSSCG00000013455 (IZUMO4), ENSSSCG00000032366 (MOB3A),

		ENSSSCG00000013448 (MKNK2), ENSSSCG00000013444 (BTBD2), ENSSSCG00000042835, ENSSSCG00000013445 (CSNK1G2), ENSSSCG00000013446 (SCAMP4), ENSSSCG00000038810 (ADAT3), ENSSSCG00000025631, ENSSSCG00000013442 (ABHD17A)	
SSC4: 35,675,562-36,727,180	0.26(18)	*ENSSSCG00000045985, ENSSSCG00000006061, ENSSSCG00000046299, ENSSSCG00000006062 (YWHAZ), ENSSSCG00000006063 (PABPC1), ENSSSCG00000006064 (SNX31), ENSSSCG00000006065 (ANKRD46), ENSSSCG00000041192, ENSSSCG00000044093, ENSSSCG00000006066 (RNF19A)*	Drip loss, Oleic acid content, monounsaturated fatty acid content
SSC10: 30,140,466-31,750,116	0.26(32)	*ENSSSCG00000010959 (NTRK2), ENSSSCG00000043924, ENSSSCG00000023147, ENSSSCG00000042859, ENSSSCG00000041561, ENSSSCG00000010960 (SLC28A3), ENSSSCG00000041849, ENSSSCG00000039950 (RMI1), ENSSSCG00000022760, ENSSSCG00000028046, ENSSSCG00000034203 (C9orf64), ENSSSCG00000021571 (KIF27), ENSSSCG00000026087 (GKAP1), ENSSSCG00000049051, ENSSSCG00000021005 (UBQLN1), ENSSSCG00000035132 (IDNK), ENSSSCG00000010962 (FRMD3), ENSSSCG00000035626*	.
SSC14: 570,328-980,734	0.25(15)	*ENSSSCG00000009580 (S1PR3), ENSSSCG00000039841 (SHC3), ENSSSCG00000021161 (CKS2), ENSSSCG00000028033 (SECISBP2), ENSSSCG00000009584 (SEMA4D)*	.
SSC6: 148,609,748-149,229,369	0.25(15)	*ENSSSCG00000003811 (ROR1), ENSSSCG00000049707, ENSSSCG00000003812 (PGM1), ENSSSCG00000003814 (EFCAB7), ENSSSCG00000037421, ENSSSCG00000020427, ENSSSCG00000042211, ENSSSCG00000003815 (ALG6), ENSSSCG00000050660, ENSSSCG00000032215 (FOXD3), ENSSSCG00000049788, ENSSSCG00000051600*	Backfat between 3rd and 4th last ribs
SSC15: 141,355,395-141,896,354	0.24(23)	.	Immunocrit
SSC10: 68,582,444-69,051,399	0.23(18)	*ENSSSCG00000011162, ENSSSCG00000042778, ENSSSCG00000011163 (DIP2C), ENSSSCG00000045476*	.

SSC7: 44,026,616-44,974,706	0.23(19)	*ENSSSCG00000001743, ENSSSCG00000036460, ENSSSCG00000001744 (TFAP2D), ENSSSCG00000025535 (TFAP2B), ENSSSCG00000047825, ENSSSCG00000043682*	Gait score (hind), age at puberty, liver weight, heart weight, head weight, kidney weight, cervical vertebra length, carcass length, carcass weight, meat color L*, adipocyte area, adipocyte perimeter
SSC15: 142,546,880-142,937,514	0.23(19)	.	.
SSC5: 95,199,760-96,141,923	0.23(18)	*ENSSSCG00000044094, ENSSSCG00000045522, ENSSSCG00000050070, ENSSSCG00000051589, ENSSSCG00000051123, ENSSSCG00000042886, ENSSSCG00000048690, ENSSSCG00000043585, ENSSSCG00000051236, ENSSSCG00000043326, ENSSSCG00000042993, ENSSSCG00000048758*	Platelet count
SSC18: 58,699,852-59,164,560	0.23(16)	.	Litter size, Feed conversion ratio
SSC5: 12,819,065-14,161,325	0.23(27)	*ENSSSCG00000000162 (BTBD11), ENSSSCG00000036971, ENSSSCG00000048045, ENSSSCG00000000164 (CRY1), ENSSSCG00000039109 (MTERF2), ENSSSCG00000038968 (TMEM263), ENSSSCG00000000167 (RIC8B), ENSSSCG00000000169 (POLR3B), ENSSSCG00000024881 (TCP11L2), ENSSSCG00000045300, ENSSSCG00000000171, ENSSSCG00000045128, ENSSSCG00000041235, ENSSSCG00000047442, ENSSSCG00000043586, ENSSSCG00000032861 (NUAK1)*	White blood cell number, carcass length, total number of piglets born alive, small intestine length
SSC2: 131,124,958-131,778,505	0.22(15)	*ENSSSCG00000014256 (FBN2), ENSSSCG00000041980, ENSSSCG00000014257 (SLC27A6)*	.
SSC18: 60,471,276-61,093,438	0.21(18)	.	Testicular length, age at puberty
SSC13: 114,884,492-116,369,233	0.21(14)	*ENSSSCG00000043568, ENSSSCG00000019249, ENSSSCG00000011760 (TBL1XR1), ENSSSCG00000036414, ENSSSCG00000041368, ENSSSCG00000041195, ENSSSCG00000046692, ENSSSCG00000049497,*	Backfat between 3rd and 4th last ribs, melanoma susceptibility

		ENSSSCG00000050167, ENSSSCG00000047445, ENSSSCG00000044965, ENSSSCG00000048906, ENSSSCG00000049288, ENSSSCG00000042021, ENSSSCG00000051451, ENSSSCG00000020389	
SSC15: 131,823,405-132,590,789	0.21(23)	*ENSSSCG00000016272 (PSMD1), ENSSSCG00000016273 (HTR2B), ENSSSCG00000019320, ENSSSCG00000023994 (ARMC9), ENSSSCG00000033015 (B3GNT7), ENSSSCG00000016275 (NCL), ENSSSCG00000019707, ENSSSCG00000018293 (SNORD20), ENSSSCG00000027560, ENSSSCG00000016277 (NMUR1), ENSSSCG00000016278, ENSSSCG00000041092, ENSSSCG00000050388, ENSSSCG00000037274 (PTMA), ENSSSCG00000016279 (PDE6D), ENSSSCG00000033334 (COPS7B), ENSSSCG00000026852 (NPPC), ENSSSCG00000049901, ENSSSCG00000022177 (DIS3L2), ENSSSCG00000045363*	Splay leg, pH 24 hours *post-mortem* (loin), total number of piglets born alive
SSC3: 121,715,613-122,235,888	0.21(15)	*ENSSSCG00000042394, ENSSSCG00000008619 (DDX1), ENSSSCG00000008621 (NBAS), ENSSSCG00000045349*	LDL cholesterol, cholesterol level
SSC3: 49,578,962-50,726,908	0.20(14)	*ENSSSCG00000025772 (GPR45), ENSSSCG00000028020 (MRPS9), ENSSSCG00000050003, ENSSSCG00000022096 (POU3F3), ENSSSCG00000042134, ENSSSCG00000045191, ENSSSCG00000041960, ENSSSCG00000042443, ENSSSCG00000036710*	CD8-negative leukocyte percentage, CD8-positive leukocyte percentage, lactate level, body temperature, platelet count
SSC6: 14,620,144-15,144,746	0.20(20)	*ENSSSCG00000002734, ENSSSCG00000002739 (PHLPP2), ENSSSCG00000002740 (AP1G1), ENSSSCG00000019395 (SNORD71), ENSSSCG00000035474, ENSSSCG00000038917 (ATXN1L), ENSSSCG00000002743 (IST1), ENSSSCG00000002741 (ZNF821), ENSSSCG00000002746 (DHODH), ENSSSCG00000002749, ENSSSCG00000050689, ENSSSCG00000025218 (TXNL4B), ENSSSCG00000002748 (DHX38), ENSSSCG00000002747 (PMFBP1)*	Unsaturated fatty acid content
SSC1: 242,598-947,141	0.20(10)	*ENSSSCG00000043002, ENSSSCG00000050193, ENSSSCG00000046031, ENSSSCG00000042397, ENSSSCG00000035944, ENSSSCG00000047845, ENSSSCG00000004010 (ERMARD), ENSSSCG00000004009 (TCTE3), ENSSSCG00000030155 (PHF10), ENSSSCG00000038931 (C6orf120), ENSSSCG00000004008, ENSSSCG00000045488, ENSSSCG00000048735,*	Arachidonic acid to dihomo-gamma-linolenic acid ratio, arachidonic acid to eicosadienoic acid ratio, backfat at rump

		ENSSSCG00000049501, ENSSSCG00000004012 (THBS2), ENSSSCG00000035661	
SSC3: 126,974,179-127,967,288	0.20(17)	*ENSSSCG00000008642 (ASAP2), ENSSSCG00000047198, ENSSSCG00000045751, ENSSSCG00000023539 (MBOAT2), ENSSSCG00000008644 (KIDINS220), ENSSSCG00000008645, ENSSSCG00000048192, ENSSSCG00000045283, ENSSSCG00000049370, ENSSSCG00000043395, ENSSSCG00000045206, ENSSSCG00000049451, ENSSSCG00000050833, ENSSSCG00000043816, ENSSSCG00000041417, ENSSSCG00000045752, ENSSSCG00000045758, ENSSSCG00000031948*	Glucose level, body weight (birth), LDL cholesterol, cholesterol level, age at puberty
SSC15: 35,281,500-35,588,121	0.19(15)	*ENSSSCG00000049782, ENSSSCG00000046047, ENSSSCG00000049477, ENSSSCG00000046454, ENSSSCG00000044121*	.
SSC4: 78,946,182-79,413,481	0.19(13)	*ENSSSCG00000034495 (SNTG1), ENSSSCG00000035744, ENSSSCG00000039568 (SNAI2), ENSSSCG00000051418, ENSSSCG00000025391, ENSSSCG00000032052, ENSSSCG00000048282, ENSSSCG00000032887 (EFCAB1), ENSSSCG00000046405*	Backfat between 3rd and 4th last ribs, hemoglobin
SSC2: 156,302,473-157,412,049	0.19(25)	.	Body weight, age at puberty, Arachidonic acid to dihomo-gamma-linolenic acid ratio, Dihomo-gamma-linolenic acid to linoleic acid ratio, backfat at 10th rib, backfat at last rib, phosphate level, loin muscle area
SSC12: 46,377,660-46,855,900	0.19(15)	*ENSSSCG00000017796 (BLMH), ENSSSCG00000017798 (TMIGD1), ENSSSCG00000018963, ENSSSCG00000017799 (CPD), ENSSSCG00000029262 (GOSR1), ENSSSCG00000017801, ENSSSCG00000017802 (BHLHA9), ENSSSCG00000046847, ENSSSCG00000017804 (ABR)*	Arachidic acid content, cis-11-Eicosenoic acid content, intramuscular fat content, Linoleic acid content, Polyunsaturated fatty acid content, Omega-6 fatty acid content
SSC1: 91,489,854-92,478,641	0.19(17)	*ENSSSCG00000044069, ENSSSCG00000004485 (CD109), ENSSSCG00000050469, ENSSSCG00000004486 (SLC17A5), ENSSSCG00000004489 (EEF1A1), ENSSSCG00000037674, ENSSSCG00000004487 (MTO1)*	Mean corpuscular hemoglobin content

SSC10: 50,418,539-50,778,647	0.19(13)	*ENSSSCG00000028135 (PRTFDC1), ENSSSCG00000037690, ENSSSCG00000011074 (ARHGAP21), ENSSSCG00000011075 (KIAA1217)*	.
SSC11: 20,388,606-21,313,819	0.19(17)	*ENSSSCG00000043883, ENSSSCG00000047786, ENSSSCG00000051133, ENSSSCG00000037452 (HTR2A), ENSSSCG00000046304, ENSSSCG00000009407 (ESD), ENSSSCG00000045677, ENSSSCG00000009408 (LRCH1), ENSSSCG00000009410 (RUBCNL), ENSSSCG00000009411 (LRRC63), ENSSSCG00000009412 (LCP1), ENSSSCG00000034648, ENSSSCG00000009413 (CPB2), ENSSSCG00000009414 (ZC3H13)*	Front leg conformation, hind leg conformation, gait score (overall)
SSC18: 59,655,603-60,133,742	0.19(15)	.	.
SSC10: 33,859,597-34,781,511	0.19(12)	*ENSSSCG00000011003, ENSSSCG00000030408 (DDX58), ENSSSCG00000011004 (TOPORS), ENSSSCG00000023807 (ACO1), ENSSSCG00000043981, ENSSSCG00000039263, ENSSSCG00000046623, ENSSSCG00000051410, ENSSSCG00000044813, ENSSSCG00000051170*	Cervical vertebra length, immunocrit
SSC3: 14,772,381-15,010,975	0.18(12)	*ENSSSCG00000007727, ENSSSCG00000049007*	.
SSC7: 65,166,653-66,796,849	0.18(17)	*ENSSSCG00000040912, ENSSSCG00000032041, ENSSSCG00000034590, ENSSSCG00000037026, ENSSSCG00000039278, ENSSSCG00000039726, ENSSSCG00000001960 (EAPP), ENSSSCG00000048543, ENSSSCG00000042840, ENSSSCG00000044687, ENSSSCG00000045122, ENSSSCG00000047592, ENSSSCG00000001963 (EGLN3), ENSSSCG00000036652, ENSSSCG00000001964 (NPAS3), ENSSSCG00000047905*	Dressing percentage, Obesity index, age at puberty, Eicosadienoic acid content, conductivity 45 minutes *post-mortem*, meat color L*
SSC13: 215,235,674-215,787,385	0.18(12)	.	.
SSC4: 110,552,282-111,203,912	0.18(12)	*ENSSSCG00000006824 (GNAI3), ENSSSCG00000006825 (GPR61), ENSSSCG00000034883 (AMIGO1), ENSSSCG00000006827 (CYB561D1), ENSSSCG00000006828 (ATXN7L2), ENSSSCG00000006829 (SYPL2),*	Hind leg conformation, intramuscular fat content

		ENSSSCG00000006830 (PSMA5), ENSSSCG00000006831 (SORT1), ENSSSCG00000050471, ENSSSCG00000006834 (MYBPHL), ENSSSCG00000036975 (PSRC1), ENSSSCG00000034360 (CELSR2), ENSSSCG00000006835 (SARS), ENSSSCG00000006836 (KIAA1324), ENSSSCG00000033292 (C1orf194), ENSSSCG00000018682, ENSSSCG00000037816 (TMEM167B), ENSSSCG00000020842, ENSSSCG00000006840 (WDR47), ENSSSCG00000006841 (CLCC1), ENSSSCG00000021440 (GPSM2), ENSSSCG00000006842	
SSC8: 89,842,164-90,857,359	0.18(13)	*ENSSSCG00000051283, ENSSSCG00000050034*	.
SSC2: 160,085,066-160,809,717	0.18(14)	.	.
SSC17: 64,680,839-64,982,346	0.18(14)	.	.
SSC9: 28,523,192-28,925,416	0.18(14)	.	Adrenal gland weight, hind leg conformation
SSC3: 128,063,568-128,395,347	0.17(13)	*ENSSSCG00000048889, ENSSSCG00000046300*	Small intestine length
SSC10: 65,833,767-66,229,891	0.17(13)	*ENSSSCG00000044272, ENSSSCG00000042764, ENSSSCG00000042579, ENSSSCG00000041200, ENSSSCG00000043435, ENSSSCG00000041177, ENSSSCG00000047695, ENSSSCG00000043797, ENSSSCG00000047071*	Corpus luteum number
SSC16: 69,967,228-70,251,797	0.17(10)	*ENSSSCG00000019555*	.
SSC10: 8,509,342-8,735,370	0.17(12)	*ENSSSCG00000047944, ENSSSCG00000044167, ENSSSCG00000043065, ENSSSCG00000046752, ENSSSCG00000038539, ENSSSCG00000041804, ENSSSCG00000049292, ENSSSCG00000044961*	pH 24 hours *post-mortem* (ham)

SSC1: 107,075,251-108,648,750	0.17(13)	*ENSSSCG00000004545 (ANKDD1A), ENSSSCG00000037998, ENSSSCG00000044010, ENSSSCG00000004547, ENSSSCG00000004548 (PIF1), ENSSSCG00000004549 (RBPMS2), ENSSSCG00000004550 (OAZ2), ENSSSCG00000004551 (ZNF609), ENSSSCG00000041571, ENSSSCG00000004552 (TRIP4), ENSSSCG00000004554, ENSSSCG00000045500, ENSSSCG00000004558 (CSNK1G1), ENSSSCG00000034137 (PPIB), ENSSSCG00000037275 (SNX22), ENSSSCG00000042703, ENSSSCG00000048062, ENSSSCG00000047502, ENSSSCG00000034124 (CIAO2A), ENSSSCG00000021588 (DAPK2), ENSSSCG00000004561 (HERC1), ENSSSCG00000043545, ENSSSCG00000038786, ENSSSCG00000028737 (USP3), ENSSSCG00000040895, ENSSSCG00000047861, ENSSSCG00000032949 (APH1B), ENSSSCG00000004565 (CA12)*	Litter size, corpus luteum number, HDL/LDL ratio, drip loss
SSC13: 117,757,022-118,601,930	0.17(10)	*ENSSSCG00000011765 (USP13), ENSSSCG00000011766 (PEX5L), ENSSSCG00000046936, ENSSSCG00000042124*	.
SSC6: 151,680,316-152,263,991	0.17(12)	*ENSSSCG00000050509, ENSSSCG00000046052, ENSSSCG00000042411, ENSSSCG00000003823 (C1orf87)*	.
SSC4: 113,883,126-114,151,582	0.16(11)	.	Skin thickness
SSC1: 93,695,736-96,303,394	0.16(11)	*ENSSSCG00000008882, ENSSSCG00000040747, ENSSSCG00000044547, ENSSSCG00000041974, ENSSSCG00000004490 (SETBP1), ENSSSCG00000045948, ENSSSCG00000050379, ENSSSCG00000051783, ENSSSCG00000050905, ENSSSCG00000051009, ENSSSCG00000004491 (SLC14A2), ENSSSCG00000004492 (SLC14A1), ENSSSCG00000004493 (SIGLEC15), ENSSSCG00000021072 (EPG5), ENSSSCG00000004499, ENSSSCG00000004498, ENSSSCG00000004500 (C18orf25), ENSSSCG00000041792, ENSSSCG00000051447, ENSSSCG00000004502 (RNF165), ENSSSCG00000004503 (LOXHD1), ENSSSCG00000050304, ENSSSCG00000032053 (ST8SIA5)*	Feed conversion ratio, age at puberty, removal parity, mean corpuscular hemoglobin content, mean corpuscular volume

SSC3: 14,376,309-14,569,226	0.16(10)	*ENSSSCG00000007727, ENSSSCG00000018380*	pH 45 minutes *post-mortem*, 45 min-24-hour pH decline
SSC13: 32,626,695-32,906,051	0.16(11)	*ENSSSCG00000011398 (SEMA3F), ENSSSCG00000042718 (GNAT1), ENSSSCG00000011397 (SLC38A3), ENSSSCG00000011399 (GNAI2), ENSSSCG00000011400 (SEMA3B), ENSSSCG00000011401 (LSMEM2), ENSSSCG00000011402 (IFRD2), ENSSSCG00000041673, ENSSSCG00000024363 (HYAL3), ENSSSCG00000011404, ENSSSCG00000011405 (HYAL2), ENSSSCG00000011407 (RASSF1), ENSSSCG00000011408 (ZMYND10), ENSSSCG00000011410 (CYB561D2), ENSSSCG00000043195 (TMEM115), ENSSSCG00000011412 (CACNA2D2)*	.
SSC1: 1,994,718-2,627,242	0.16(13)	*ENSSSCG00000032403, ENSSSCG00000037137, ENSSSCG00000024562 (CCR6), ENSSSCG00000034196, ENSSSCG00000004023 (FGFR1OP), ENSSSCG00000043814, ENSSSCG00000004024, ENSSSCG00000048164, ENSSSCG00000043922, ENSSSCG00000004022 (RPS6KA2), ENSSSCG00000048885, ENSSSCG00000032916, ENSSSCG00000004020, ENSSSCG00000051382, ENSSSCG00000049363*	.
SSC16: 438,022-842,912	0.16(13)	*ENSSSCG00000016780 (CTNND2), ENSSSCG00000018664*	Coping behavior
SSC14: 141,967,057-142,089,249	0.16(10)	.	.
SSC1: 25,927,201-26,506,878	0.16(10)	*ENSSSCG00000004149 (NHSL1), ENSSSCG00000020384, ENSSSCG00000004150, ENSSSCG00000048346 (SMIM28), ENSSSCG00000037315, ENSSSCG00000004151, ENSSSCG00000022968 (ARFGEF3), ENSSSCG00000046952, ENSSSCG00000045653, ENSSSCG00000042538, ENSSSCG00000038420, ENSSSCG00000004154 (TNFAIP3)*	Dressing percentage
SSC1: 251,551,053-252,189,140	0.16(10)	*ENSSSCG00000005455 (SVEP1), ENSSSCG00000050248, ENSSSCG00000005456 (MUSK), ENSSSCG00000005457 (LPAR1), ENSSSCG00000044924*	.

SSC2: 139,196,714-139,571,078	0.16(11)	*ENSSSCG00000032423 (SPOCK1)*	Intramuscular fat content
SSC18: 56,474,257-57,050,998	0.16(10)	.	Testicular width
SSC2: 4,145,839-4,614,073	0.16(11)	*ENSSSCG00000042011, ENSSSCG00000043204, ENSSSCG00000012878, ENSSSCG00000012879, ENSSSCG00000012880, ENSSSCG00000012882 (TESMIN), ENSSSCG00000012883, ENSSSCG00000012884 (PPP6R3), ENSSSCG00000012885*	Dressing percentage, shear force at first peak, meat color a*, teat number
SSC1: 27,306,383-27,672,943	0.15(11)	*ENSSSCG00000004160 (PEX7), ENSSSCG00000020379, ENSSSCG00000050757, ENSSSCG00000026041 (MAP3K5), ENSSSCG00000046709, ENSSSCG00000039049 (MAP7)*	.
SSC15: 32,017,591-32,539,459	0.15(13)	*ENSSSCG00000027105 (TUBGCP5), ENSSSCG00000019095, ENSSSCG00000031951, ENSSSCG00000050316, ENSSSCG00000048648, ENSSSCG00000045888, ENSSSCG00000036898 (NIPA2), ENSSSCG00000040778 (NIPA1), ENSSSCG00000023665 (FBXO25), ENSSSCG00000044544, ENSSSCG00000039562 (TDRP), ENSSSCG00000028195*	Monocyte percentage, tibia length
SSC3: 16,403,039-16,652,462	0.15(10)	*ENSSSCG00000007737 (TPST1), ENSSSCG00000018775, ENSSSCG00000032119 (CRCP), ENSSSCG00000019976, ENSSSCG00000007738, ENSSSCG00000047936*	45 min-24-hour pH decline, pH 24 hours *post-mortem* (ham)
SSC2: 12,082,068-12,605,988	0.15(18)	*ENSSSCG00000045087, ENSSSCG00000036715, ENSSSCG00000040765, ENSSSCG00000037942, ENSSSCG00000031303, ENSSSCG00000050458, ENSSSCG00000013144, ENSSSCG00000042706, ENSSSCG00000013145 (DTX4), ENSSSCG00000033703 (FAM111A), ENSSSCG00000013147 (FAM111B), ENSSSCG00000013148 (GLYATL2), ENSSSCG00000040088, ENSSSCG00000042844, ENSSSCG00000013151, ENSSSCG00000013155, ENSSSCG00000021176 (LPXN), ENSSSCG00000013157 (OR5B21)*	Obesity index, biceps brachii weight, feed conversion ratio, average daily gain, average backfat thickness, mean corpuscular hemoglobin content
SSC15: 22,584,606-23,011,779	0.15(12)	*ENSSSCG00000046326, ENSSSCG00000041988, ENSSSCG00000046908*	.

SSC15: 137,159,347-137,449,752	0.15(13)	*ENSSSCG00000032849 (MLPH), ENSSSCG00000036981 (PRLH), ENSSSCG00000016328 (RAB17), ENSSSCG00000050312, ENSSSCG00000016330 (LRRFIP1), ENSSSCG00000016332 (RBM44)*	White blood cell number, feed intake per feeding, head weight
SSC11: 67,401,150-67,859,534	0.15(12)	*ENSSSCG00000026863 (FARP1), ENSSSCG00000009511 (STK24), ENSSSCG00000009513, ENSSSCG00000022112 (DOCK9), ENSSSCG00000033940*	Lean meat percentage
SSC13: 106,130,701-107,338,079	0.15(10)	*ENSSSCG00000023393 (ZBBX), ENSSSCG00000044118, ENSSSCG00000050873, ENSSSCG00000045530, ENSSSCG00000011737 (SERPINI2), ENSSSCG00000011738 (WDR49), ENSSSCG00000011739 (PDCD10), ENSSSCG00000011740 (SERPINI1), ENSSSCG00000045704, ENSSSCG00000040950, ENSSSCG00000044612, ENSSSCG00000011741 (GOLIM4)*	.
SSC16: 34,938,863-35,212,148	0.15(10)	*ENSSSCG00000016913 (DDX4), ENSSSCG00000016915 (IL31RA), ENSSSCG00000016916 (IL6ST)*	Feed conversion ratio, Arachidic acid to stearic acid ratio
SSC9: 44,822,332-45,073,381	0.15(10)	*ENSSSCG00000015082 (DSCAML1)*	.
SSC5: 81,555,186-82,123,688	0.15(11)	*ENSSSCG00000045039, ENSSSCG00000046391, ENSSSCG00000048211, ENSSSCG00000042336, ENSSSCG00000000857 (IGF1), ENSSSCG00000000859 (PARPBP), ENSSSCG00000048040, ENSSSCG00000000858, ENSSSCG00000018311, ENSSSCG00000000860 (NUP37)*	Triglyceride level
SSC3: 132,380,008-132,623,706	0.15(10)	*ENSSSCG00000033193 (TPO), ENSSSCG00000036880, ENSSSCG00000042966, ENSSSCG00000047203*	.
SSC10: 14,795,101-15,127,469	0.15(11)	*ENSSSCG00000010862 (CDC42BPA), ENSSSCG00000042709, ENSSSCG00000042375, ENSSSCG00000010863 (AHCTF1)*	.
SSC3: 141,647,496-142,275,000	0.15(11)	.	.

SSC17: 11,112,492-11,436,064	0.15(10)	*ENSSSCG00000007033 (AP3M2), ENSSSCG00000007032, ENSSSCG00000007030 (IKBKB), ENSSSCG00000007031 (POLB), ENSSSCG00000007029 (DKK4), ENSSSCG00000007026 (VDAC3), ENSSSCG00000050884, ENSSSCG00000007027 (SLC20A2)*	Hematocrit
SSC2: 23,647,170-24,122,841	0.14(10)	*ENSSSCG00000045070*	.
SSC13: 58,542,282-58,873,140	0.14(11)	*ENSSSCG00000043264, ENSSSCG00000045742, ENSSSCG00000046940*	.
SSC5: 11,864,200-12,193,129	0.14(10)	*ENSSSCG00000000152 (RBFOX2), ENSSSCG00000046062, ENSSSCG00000000151, ENSSSCG00000040453 (SYN3), ENSSSCG00000047089, ENSSSCG00000031866 (TIMP3)*	.
SSC9: 6,393,773-6,721,992	0.14(10)	*ENSSSCG00000014794 (NUP98), ENSSSCG00000014796 (CHRNA10), ENSSSCG00000038255 (ART1), ENSSSCG00000014799, ENSSSCG00000048448, ENSSSCG00000048507, ENSSSCG00000014800 (RNF121), ENSSSCG00000023796 (IL18BP), ENSSSCG00000014802 (NUMA1), ENSSSCG00000014803, ENSSSCG00000014804 (LAMTOR1), ENSSSCG00000014805, ENSSSCG00000014806 (ANAPC15), ENSSSCG00000050958*	Red blood cell count, hematocrit
SSC3: 95,385,808-96,071,798	0.14(10)	*ENSSSCG00000043255, ENSSSCG00000008446 (SIX2), ENSSSCG00000039283 (SIX3), ENSSSCG00000041282, ENSSSCG00000008447 (CAMKMT), ENSSSCG00000040635*	CD3-negative

Location: indicates the chromosome number (SSC1 to SSC18), followed by the starting and end position of the genomic window(s) in base pairs; **Var(%)**: the percental variance explained by the most relevant 10-SNP window within the region of interest; **N SNP:** number of SNP marker included in the genomic region identified; **Positional genes:** Ensembl gene stable ID (with official gene symbol when known) mapped to the genomic regions identified; **QTL traits**: indicates which traits have known QTLs mapped within the genomic region of interest, based on QTLs reported in the Pig QTL database (https://www.animalgenome.org/cgi-bin/QTLdb/SS/index).
