# Supplementary material for: Genomics of Heat Tolerance in Reproductive Performance Investigated in Four Independent Maternal Lines of Pigs
Source: Front Genet. 2020 Jun 30;11:629. doi: 10.3389/fgene.2020.00629 (PMC7338773; doi:10.3389/fgene.2020.00629)
Supplement: Figure S1a — Additive genetic variance estimates (posterior means with 95% empirical confidence intervals) for total number of piglets born (TNB) in the Smithfield Premium Genetics Landrace population (SPG_LR) over the range of Average Relative Humidity 14 to 7 days before conception. [file Data_Sheet_1.zip › Supplementary files/SupplementaryTableS2b.pdf]

Table S2b: Significant genomic windows for the slope term of the reaction norm on total number of piglets born (TNB) as regressed on the Average Relative Humidity 14 to 7 days before conception in the Smithfield Premium Genetics (SPG) Landrace population (SPG_LR).

Location	Var(%) and N SNPs in brackets	Positional genes	QTL traits
SSC3: 35,273,575-36,463,191	0.32(23)	*ENSSSCG00000007917 (RBFOX1), ENSSSCG00000051042, ENSSSCG00000046506, ENSSSCG00000020433, ENSSSCG00000051435*	CD8-negative leukocyte percentage, CD8-positive leukocyte percentage, CD3-negative
SSC3: 60,835,887-62,535,813	0.31(18)	*ENSSSCG00000035540*	Feed conversion ratio, backfat thickness between 3rd and 4th rib, ham weight, CD8-positive leukocyte percentage, CD3-negative, CD8-negative leukocyte percentage, average daily gain, Japanese color scale, meat color L*
SSC11: 80,629,582-81,387,249	0.31(19)	.	Conductivity 45 minutes *post-mortem*, hind leg conformation
SSC9: 28,682,617-29,350,733	0.28(17)	*ENSSSCG00000039906, ENSSSCG00000020347*	Litter weight, adrenal gland weight, hind leg conformation, gestation length
SSC10: 17,948,352-18,287,728	0.26(10)	*ENSSSCG00000010879 (KIF26B), ENSSSCG00000022034*	Intramuscular fat content, CD4-positive, total number of piglets born alive, melanoma susceptibility
SSC14: 37,500,868-39,015,629	0.26(19)	*ENSSSCG00000049452, ENSSSCG00000043926, ENSSSCG00000009865 (TBX3), ENSSSCG00000009866, ENSSSCG00000009867 (TBX5), ENSSSCG00000019229, ENSSSCG00000009868 (RBM19), ENSSSCG00000042669, ENSSSCG00000009869 (LHX5), ENSSSCG00000009870 (SDSL), ENSSSCG00000036865 (SDS), ENSSSCG00000009872 (PLBD2), ENSSSCG00000047984, ENSSSCG00000009873 (DTX1),*	Obesity index, saturated fatty acid content, pH 45 minutes *post-mortem*

		ENSSSCG00000009874 (RASAL1), ENSSSCG00000009875 (CFAP73), ENSSSCG00000009876 (DDX54), ENSSSCG00000009879 (TPCN1), ENSSSCG00000034479 (RITA1), ENSSSCG00000009877 (IQCD), ENSSSCG00000009880 (SLC8B1), ENSSSCG00000009881 (OAS2), ENSSSCG00000046640 (OAS1), ENSSSCG00000009883 (RPH3A)	
SSC14: 96,986,147-98,057,493	0.24(22)	*ENSSSCG00000010427, ENSSSCG00000010428, ENSSSCG00000050078, ENSSSCG00000010429 (PRKG1), ENSSSCG00000050287*	Feed conversion ratio, average daily gain
SSC18: 40,268,397-40,910,375	0.23(16)	*ENSSSCG00000024754 (BBS9), ENSSSCG00000042909, ENSSSCG00000038919 (RP9), ENSSSCG00000049414, ENSSSCG00000022912 (NT5C3A), ENSSSCG00000021250, ENSSSCG00000022464 (KBTBD2), ENSSSCG00000023445 (AVL9), ENSSSCG00000043449, ENSSSCG00000047223, ENSSSCG00000026064, ENSSSCG00000048227, ENSSSCG00000048859, ENSSSCG00000021095, ENSSSCG00000016668 (PDE1C)*	.
SSC3: 99,313,315-100,285,031	0.22(21)	*ENSSSCG00000048362, ENSSSCG00000043111, ENSSSCG00000048793, ENSSSCG00000045020, ENSSSCG00000050296, ENSSSCG00000036418 (SLC8A1), ENSSSCG00000050402, ENSSSCG00000043827*	CD3-negative, corpus luteum number, feed conversion ratio, backfat between 3rd and 4th last ribs
SSC13: 14,054,487-14,296,693	0.22(13)	*ENSSSCG00000046221, ENSSSCG00000049580, ENSSSCG00000040057 (EOMES), ENSSSCG00000041500*	.
SSC3: 36,562,403-37,226,080	0.21(12)	*ENSSSCG00000007917 (RBFOX1)*	CD8-negative leukocyte percentage, CD8-positive leukocyte percentage, maternal infanticide
SSC10: 46,555,218-47,088,725	0.20(14)	*ENSSSCG00000011047 (FAM171A1), ENSSSCG00000026499 (NMT2), ENSSSCG00000032014, ENSSSCG00000027110 (RPP38), ENSSSCG00000045562, ENSSSCG00000038184 (ACBD7), ENSSSCG00000025759, ENSSSCG00000034119, ENSSSCG00000011049 (DCLRE1C), ENSSSCG00000011048 (SUV39H2), ENSSSCG00000011051 (HSPA14), ENSSSCG00000039658*	Mean platelet volume, skatole, functional teat number

SSC14: 144,236,610-144,483,865	0.20(14)	.	Coping behavior
SSC14: 136,565,684-136,939,847	0.20(13)	*ENSSSCG00000035045 (DOCK1), ENSSSCG00000010751 (NPS)*	Total number of piglets born alive, litter size, intramuscular fat content
SSC1: 208,516,673-209,787,256	0.19(12)	*ENSSSCG00000051701, ENSSSCG00000005191 (MPDZ), ENSSSCG00000051645, ENSSSCG00000034928, ENSSSCG00000036360 (LURAP1L), ENSSSCG00000005193 (TYRP1)*	.
SSC18: 45,539,953-45,766,672	0.19(11)	*ENSSSCG00000016708 (SKAP2), ENSSSCG00000041996*	Red blood cell count
SSC5: 5,515,322-5,693,454	0.18(12)	*ENSSSCG00000051106, ENSSSCG00000000029 (SCUBE1)*	Hind feet conformation
SSC11: 17,102,723-17,987,136	0.18(10)	*ENSSSCG00000049891, ENSSSCG00000049814, ENSSSCG00000045895, ENSSSCG00000048645, ENSSSCG00000049693, ENSSSCG00000049008, ENSSSCG00000041668, ENSSSCG00000046542, ENSSSCG00000051275, ENSSSCG00000048598, ENSSSCG00000049854, ENSSSCG00000051290, ENSSSCG00000046277, ENSSSCG00000051086, ENSSSCG00000041698, ENSSSCG00000019092, ENSSSCG00000019808, ENSSSCG00000041341, ENSSSCG00000046352, ENSSSCG00000045213, ENSSSCG00000009388 (KCNRG), ENSSSCG00000009389 (TRIM13), ENSSSCG00000009390, ENSSSCG00000024125 (KPNA3)*	.
SSC14: 121,766,701-123,117,472	0.18(14)	*ENSSSCG00000042781, ENSSSCG00000041222, ENSSSCG00000046091, ENSSSCG00000050661, ENSSSCG00000010631 (GPAM), ENSSSCG00000010632 (TECTB), ENSSSCG00000050321, ENSSSCG00000031789 (ACSL5), ENSSSCG00000010635 (ZDHHC6), ENSSSCG00000032968 (VTI1A)*	Monounsaturated fatty acid content, Stearic acid content, Oleic acid content, Saturated fatty acid content, Palmitic acid content, Palmitoleic acid content, Oleic acid to stearic acid ratio, Monounsaturated fatty acid to saturated

			fatty acid ratio, unsaturated to saturated fatty acid ratio, Palmitoleic acid to palmitic acid ratio, Stearic acid to palmitic acid ratio, pH 24 hour post-mortem (loin), feed conversion ratio, fatty acid atherogenic index, fatty acid thrombogenic index, double-bond index, unsaturated index, thoracic vertebra number, thoracolumbar vertebra number, average backfat thickness
SSC3: 65,784,424-66,506,595	0.18(16)	*ENSSSCG00000042171, ENSSSCG00000008259 (LRRTM4)*	CD3-negative, CD8-negative leukocyte percentage, CD8-positive leukocyte percentage
SSC7: 129,582,907-130,165,353	0.17(13)	.	.
SSC18: 35,731,229-36,469,920	0.16(17)	*ENSSSCG00000042796, ENSSSCG00000041482, ENSSSCG00000044944, ENSSSCG00000043837, ENSSSCG00000048016, ENSSSCG00000049426, ENSSSCG00000034626*	.
SSC8: 49,111,962-50,537,893	0.15(10)	*ENSSSCG00000020037, ENSSSCG00000027183 (FSTL5)*	Teat number
SSC12: 21,590,780-21,790,209	0.15(10)	*ENSSSCG00000017459 (KRT20), ENSSSCG00000017460 (KRT12), ENSSSCG00000028522 (KRT10), ENSSSCG00000017461 (KRT28), ENSSSCG00000017462, ENSSSCG00000043259, ENSSSCG00000017463 (KRT26), ENSSSCG00000017464 (KRT25), ENSSSCG00000017465 (KRT24), ENSSSCG00000034208, ENSSSCG00000036089, ENSSSCG00000039137 (SMARCE1)*	.
SSC9: 141,951,516-142,198,779	0.15(12)	.	.

SSC6: 126,692,828-127,194,692	0.15(11)	.	Backfat at 10th rib, LDL cholesterol, basophil percentage, intramuscular fat content
SSC14: 118,988,272-119,983,210	0.15(12)	*ENSSSCG00000045123, ENSSSCG00000050155, ENSSSCG00000046631, ENSSSCG00000050431, ENSSSCG00000047504, ENSSSCG00000006978*	Stearic acid content, Palmitic acid content, Saturated fatty acid content, Monounsaturated fatty acid content, Palmitoleic acid content, Oleic acid content, thoracolumbar vertebra number, Oleic acid to stearic acid ratio, Monounsaturated fatty acid to saturated fatty acid ratio, Unsaturated to saturated fatty acid ratio, Palmitoleic acid to palmitic acid ratio, Stearic acid to palmitic acid ratio
SSC2: 139,272,108-139,653,453	0.14(11)	*ENSSSCG00000032423 (SPOCK1), ENSSSCG00000014321 (KLHL3)*	Intramuscular fat content
SSC2: 136,398,371-136,554,308	0.14(10)	*ENSSSCG00000014296 (VDAC1), ENSSSCG00000030680 (TCF7), ENSSSCG00000038628 (SKP1), ENSSSCG00000014298*	.
SSC3: 62,800,988-63,130,898	0.14(10)	.	CD3-negative, CD8-negative leukocyte percentage, CD8-positive leukocyte percentage
SSC17: 6,521,746-6,946,270	0.14(10)	*ENSSSCG00000048723*	.
SSC7: 10,695,721-10,903,372	0.13(10)	*ENSSSCG00000045903, ENSSSCG00000039116, ENSSSCG00000044834*	Triglyceride level

Location: indicates the chromosome number (SSC1 to SSC18), followed by the starting and end position of the genomic window(s) in base pairs; **Var(%)**: the percental variance explained by the most relevant 10-SNP window within the region of interest; **N SNP:** number of SNP marker included in the genomic region identified; **Positional genes:** Ensembl gene stable ID (with official gene symbol when known) mapped to the genomic regions

identified; **QTL traits**: indicates which traits have known QTLs mapped within the genomic region of interest, based on QTLs reported in the Pig QTL database (https://www.animalgenome.org/cgi-bin/QTLdb/SS/index).
