# Supplementary figures and images for: Genomics of Heat Tolerance in Reproductive Performance Investigated in Four Independent Maternal Lines of Pigs
Source: Front Genet. 2020 Jun 30;11:629. doi: 10.3389/fgene.2020.00629 (PMC7338773; doi:10.3389/fgene.2020.00629)

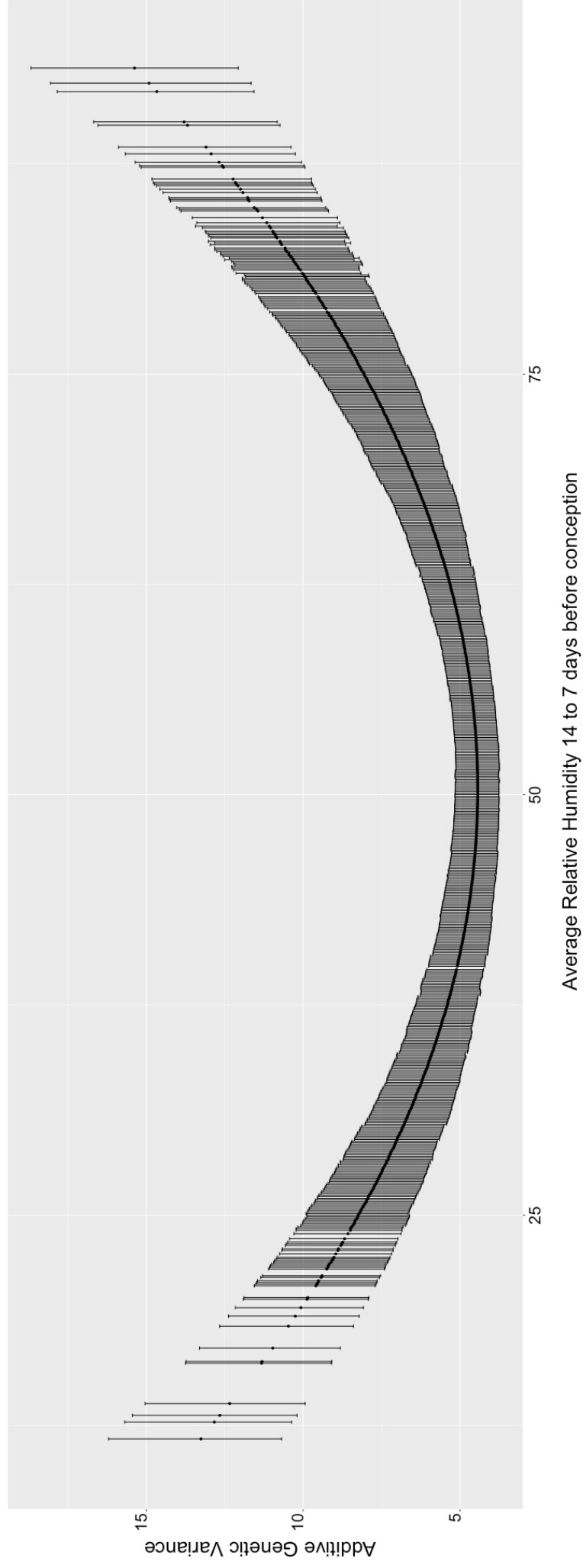

Supplement: Figure S1a — Additive genetic variance estimates (posterior means with 95% empirical confidence intervals) for total number of piglets born (TNB) in the Smithfield Premium Genetics Landrace population (SPG_LR) over the range of Average Relative Humidity 14 to 7 days before conception. [file Data_Sheet_1.zip › Supplementary files/figureS1a.pdf]

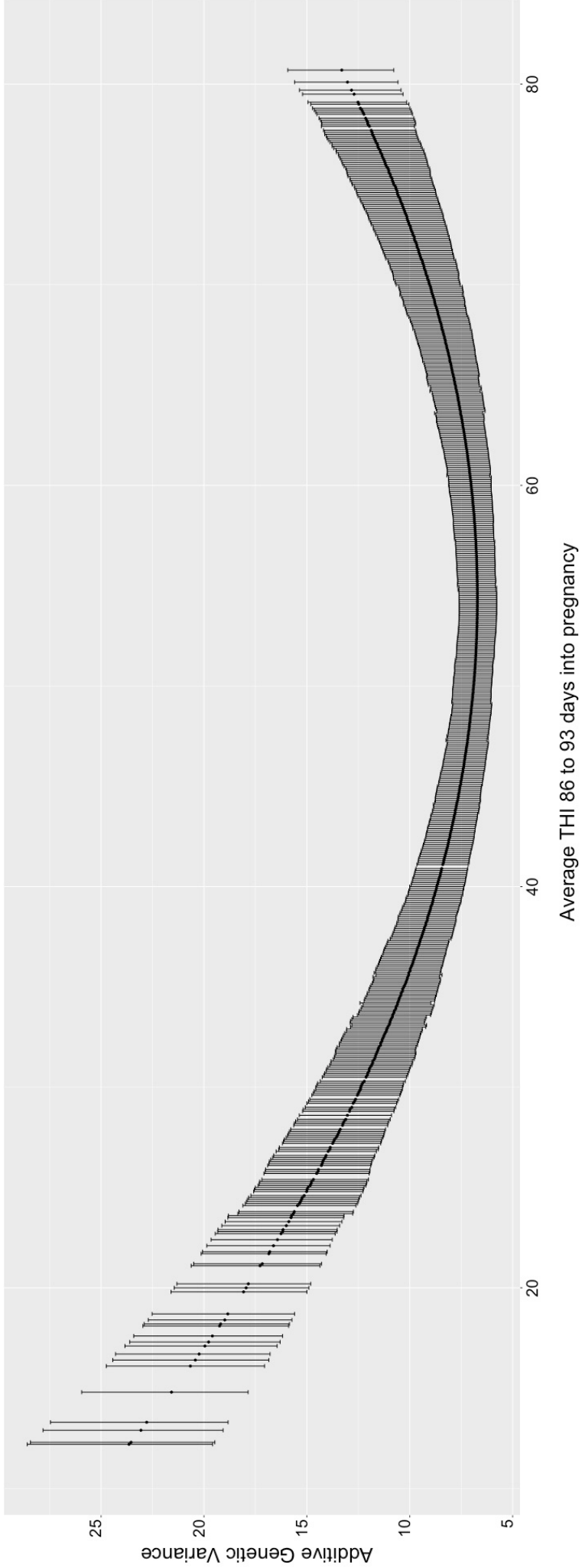

Supplement: Figure S1a — Additive genetic variance estimates (posterior means with 95% empirical confidence intervals) for total number of piglets born (TNB) in the Smithfield Premium Genetics Landrace population (SPG_LR) over the range of Average Relative Humidity 14 to 7 days before conception. [file Data_Sheet_1.zip › Supplementary files/figureS1b.pdf]

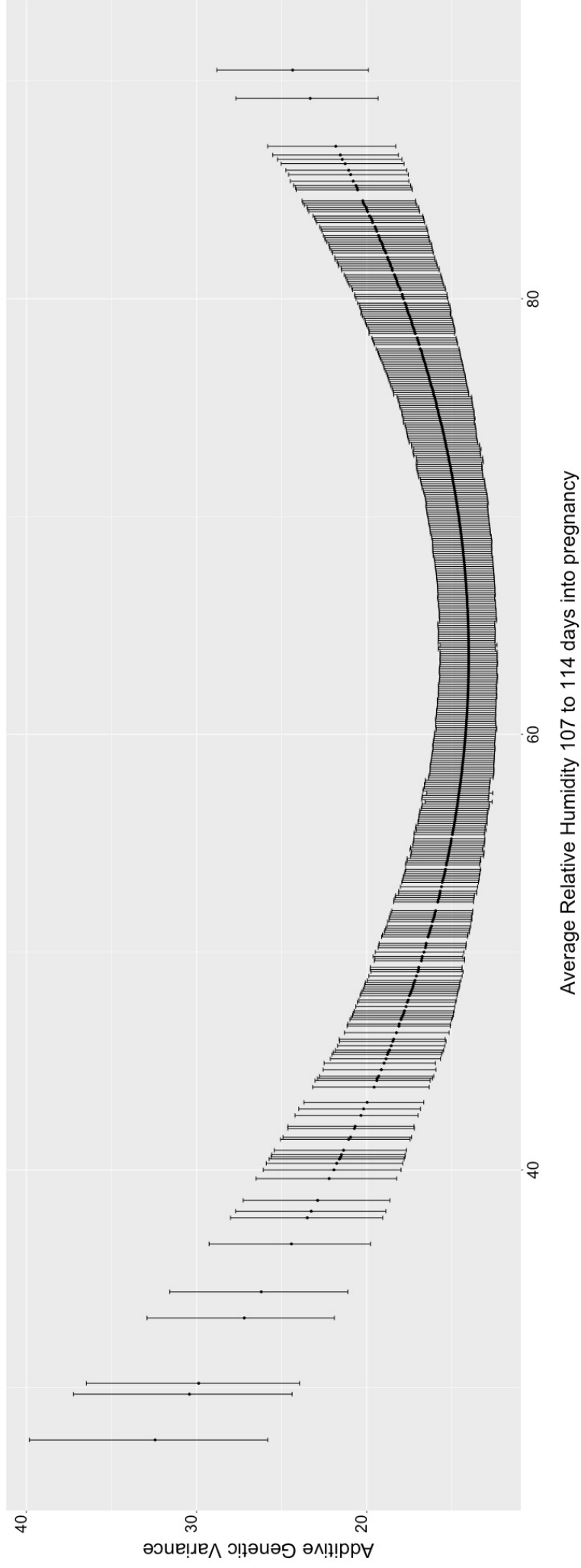

Supplement: Figure S1a — Additive genetic variance estimates (posterior means with 95% empirical confidence intervals) for total number of piglets born (TNB) in the Smithfield Premium Genetics Landrace population (SPG_LR) over the range of Average Relative Humidity 14 to 7 days before conception. [file Data_Sheet_1.zip › Supplementary files/figureS1c.pdf]

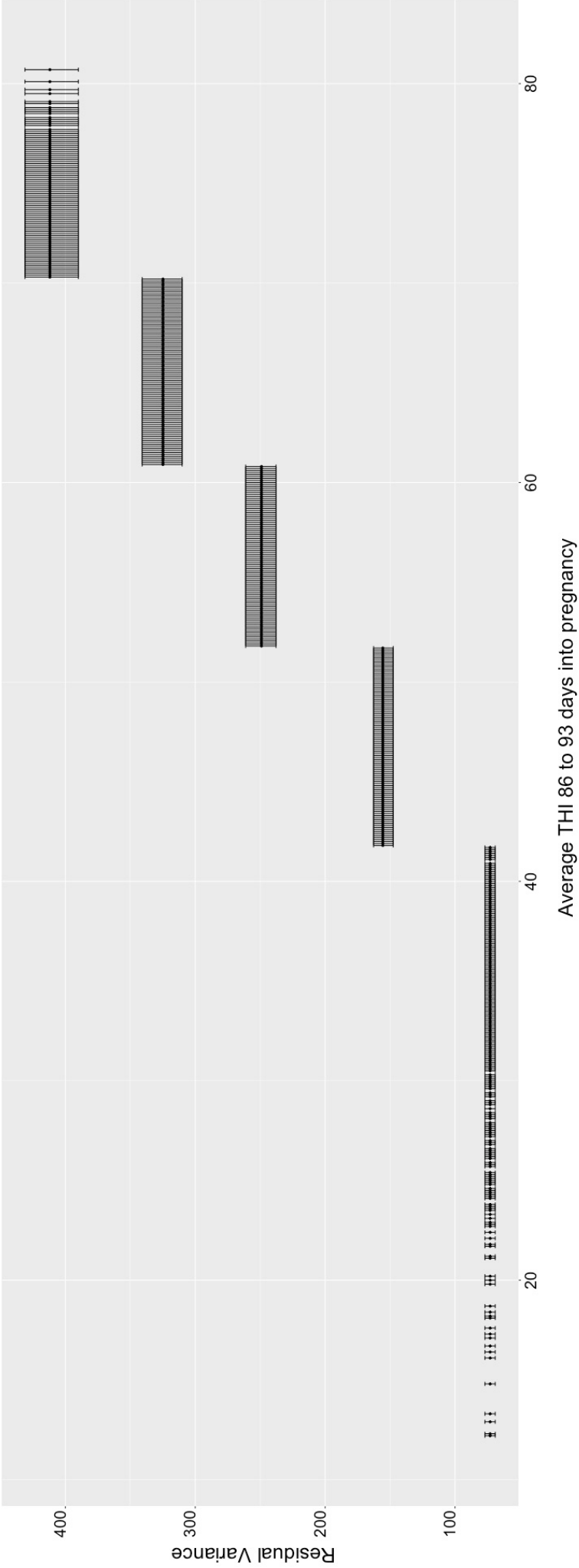

Supplement: Figure S1a — Additive genetic variance estimates (posterior means with 95% empirical confidence intervals) for total number of piglets born (TNB) in the Smithfield Premium Genetics Landrace population (SPG_LR) over the range of Average Relative Humidity 14 to 7 days before conception. [file Data_Sheet_1.zip › Supplementary files/figureS2b.pdf]

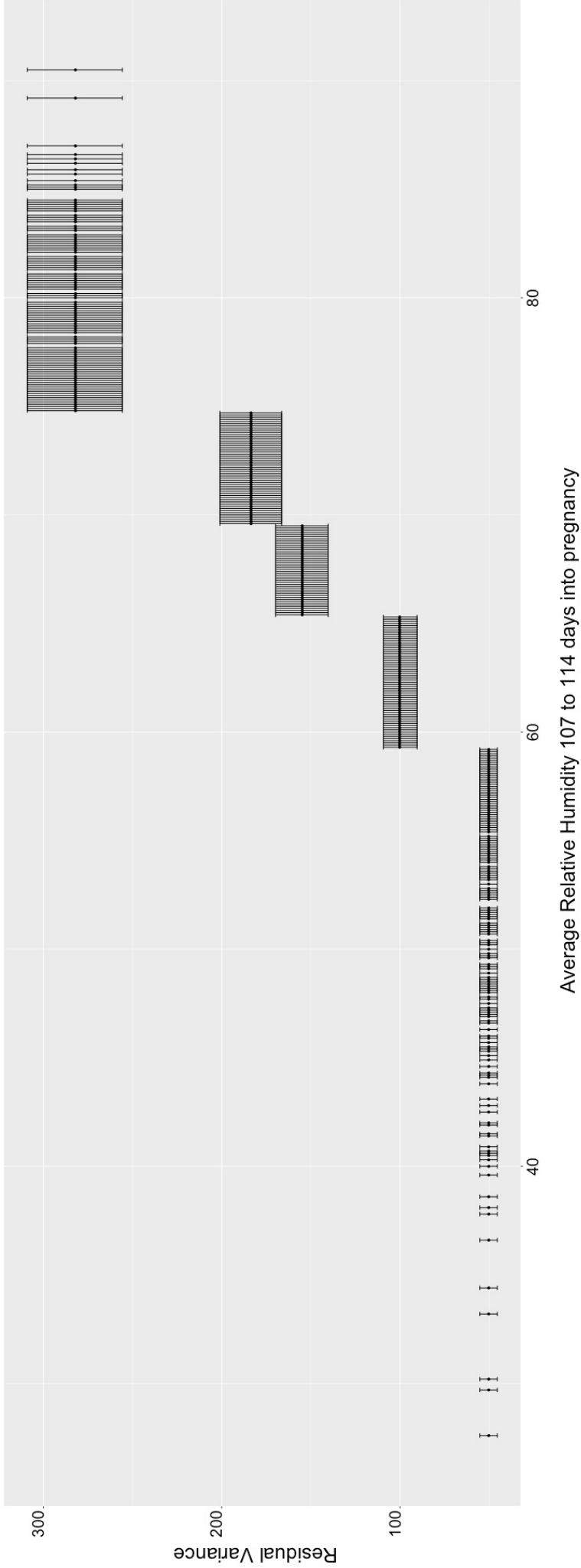

Supplement: Figure S1a — Additive genetic variance estimates (posterior means with 95% empirical confidence intervals) for total number of piglets born (TNB) in the Smithfield Premium Genetics Landrace population (SPG_LR) over the range of Average Relative Humidity 14 to 7 days before conception. [file Data_Sheet_1.zip › Supplementary files/figureS2c.pdf]

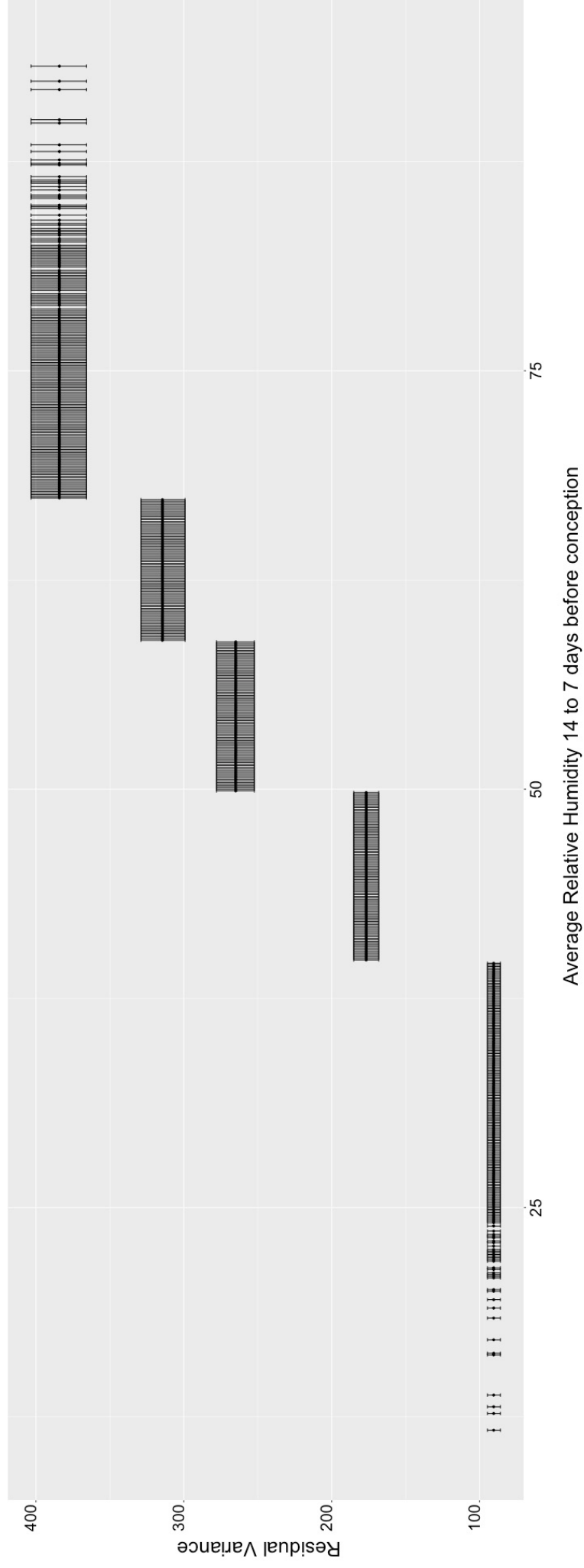

Supplement: Figure S1a — Additive genetic variance estimates (posterior means with 95% empirical confidence intervals) for total number of piglets born (TNB) in the Smithfield Premium Genetics Landrace population (SPG_LR) over the range of Average Relative Humidity 14 to 7 days before conception. [file Data_Sheet_1.zip › Supplementary files/figureS2a.pdf]
